# Global trends and insights in ethical statements regarding the utilization of human cadaveric tissues for biomechanical research from 2017 to 2022: a bibliometric analysis

**DOI:** 10.1097/JS9.0000000000001986

**Published:** 2024-07-24

**Authors:** Lenian Zhou, Yi Qiao, Qianying Cai, Hanqiang Jin, Junqing Lin, Jie Shen, Hongyi Zhu

**Affiliations:** a Department of Orthopaedics, Shanghai Sixth People’s Hospital Affiliated to Shanghai Jiao Tong University School of Medicine; b Institute of Clinical Research, National Center for Orthopaedics, Shanghai Sixth People’s Hospital Affiliated to Shanghai Jiao Tong University School of Medicine; c Medical Records and Statistics Office, Shanghai Sixth People’s Hospital Affiliated to Shanghai Jiao Tong University School of Medicine, Shanghai, People’s Republic of China

**Keywords:** cadaver, ethics, guidelines, helsinki declaration, PubMed

## Abstract

Human cadaveric tissues are essential for biomechanical research; however, omitting statements regarding the ethical implications remains very common in many recent articles of biomechanics. The authors systematically searched PubMed for research articles published from 2017 to 2022 to identify all relevant studies (*n*=1850). Information on the characteristics of the cadaveric specimens and the acquisition of ethical approval was collected. The included articles were categorized as grade A (*n*=757), grade B (*n*=308), and grade C (*n*=785) according to the authors’ predetermined criteria. Additionally, the authors analyzed whether the reporting status of ethical approval varied based on publishing year, journals, regions, and research settings. No significant improvement was observed over the study period. Disparities in ethical reporting standards were identified among different regions and journals. This is the first bibliometric analysis that offers an overview of publications in biomechanical research that use human cadaveric samples. It serves as a reference for interested researchers and journal readers to discuss the associated ethical issues.

## Introduction

Highlights

- The utilization of cadaveric tissue constitutes a profound subject, and ethical considerations are pertinent to its utilization.
- No substantial amelioration manifested throughout the duration of the study.
- Discrepancies in ethical reporting criteria were discerned among various geographical regions and academic publications.
- Further endeavors are paramount to elevate ethical awareness and augment reporting protocols.

Human cadavers are a valuable but scarce resource in medical education, particularly for biomechanical investigations in musculoskeletal and sport-related fields^1^. The main challenge in using cadaveric samples in biomechanical studies and medical teaching is their limited availability^2^. Meanwhile, the World Medical Association (WMA)‘s Declaration of Taipei explicitly recommends obtaining informed consent for any human samples used in medical research, including those from deceased individuals, and broad consent for biobanks^3^.

In the field of medical education and anatomy research, ethical considerations regarding cadaveric samples have been well-recognized in past decades. In 2012, the International Federation of Associations of Anatomists recommended that only donated bodies should be used for anatomy teaching and research^4^. Additionally, reporting guidelines for anatomy research involving cadaveric samples have also been established by editors of anatomical journals, recommending researchers to report various ethical aspects of their studies, such as Institutional Review Board (IRB) approval^5^. Despite the above efforts, however, tissue/body misuse or unconsented activities, has remained frequently documented in recent years^6–8^.

For biomechanical studies, a previous review of articles published on four leading musculoskeletal journals from 2009 to 2012 found that only 17% of articles obtained formal approval and 23% reported explicit source for the cadaveric specimens^9^. Given the updated ethical principles we mentioned above, it prompted us to conduct the present bibliometric study, which comprehensively reviewed all available biomechanical articles published from 2017 to 2022.

## Methods

**Figure 1:**
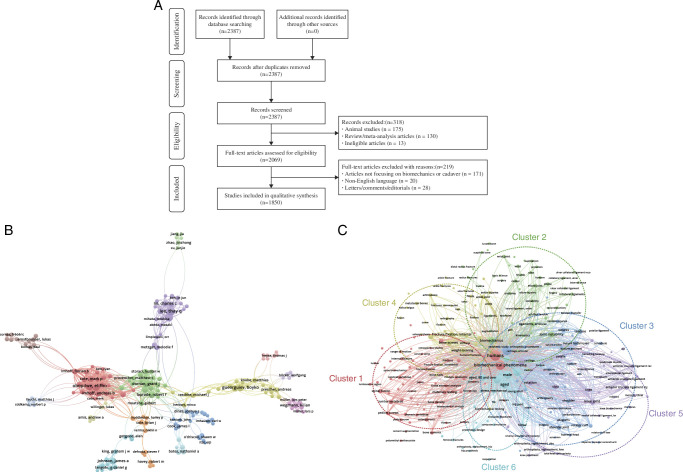
(A) Flow diagram summarizing article selection. (B) Network map of cooperation between authors (with over 5 publications). (C) Overlay visualization map of co-occurrence keywords (reported at least ten times).

We searched PubMed for all original articles published from 1 January 2017 to 31 December 2022. Searches were performed using the following terms: “biomechanical”, or “biomechanics” with either “cadaver” or “cadaveric” under the “Title/Abstract” option. After searching with the key words, we found a total of 2387 articles to assess study eligibility based on the predefined criteria. Finally, we extracted data from 1850 original articles that were eligible for inclusion (Fig. 1A).

### Grading of reporting quality on ethical information

We categorized all articles into three different grades: (A) ethical approval obtained (with and without approval number); (B) ethical approval exempted/not mentioned with explicit information on the sample sources; (C) ethical approval exempted/not mentioned with no explicit information on the sample sources. Ambiguous information on sample source (e.g. “a tissue bank”; “a biobank”, “a company”) was not considered as explicit information while targeted description (e.g. “the institutional tissue bank; “the university biobank”; “the state willed body program”) was considered as explicit information.

### Categories of countries and regions and additional data sources

The included countries and regions were divided into the following continents or subcontinents: America (North), Asia, Europe (west and central), and others based on their geographical locations as defined by the United Nations^10^. The Human Development Index (HDI) is a region-specific variable that includes three key dimensions of human development including life expectancy, education and socioeconomic status^11^. Country/region income, measured by Gross Domestic Product (GDP) per capita in US dollar, from the World Bank^12^. Total population and death estimates of each country/region from the United Nations (UN) Population Division^13^.

### Software tools and statistical analysis

Bibliometrics referred to the quantitative analysis of research publications, relying on software tools for effective analysis. Specifically, GraphPad Prism software was used to create essential statistical charts, while VOS viewer software enabled co-authorship and co-occurrence keywords analyses. Categorical variables were, respectively, presented as counts (percentage), unless otherwise indicated. The results of the survey were weighted to allow for variations in the sample. χ^2^ tests were performed in a two-sided fashion and at a *P* less than 0.05 level of significance. We used multiple linear regression to find potential associations between the grades of ethical approval and factors related to countries and regions. All statistical analysis was conducted using IBM SPSS 26.0 (Chicago, IL, USA) with any necessary extensions in this study.

## Results

### Co-authorship and co-occurrence keywords analyses

A total of 7510 authors were involved in biomechanical research utilizing of human cadaveric tissues. Figure 1B illustrates a collaborative network of 297 authors, each with over 5 publications, divided into 15 clusters. In total, the research encompassed 4885 keywords. Figure 1C depicts the network visualization of keywords that were reported at least ten times, divided into 6 clusters consisting of 276 keywords. Terms that were closely correlated were grouped together in the same cluster during co-occurrence analysis. Notably, these clusters appeared to be categorized based on the anatomical sites studied.

### Trends of reporting quality on ethical information from 2017 to 2022

**Figure 2:**
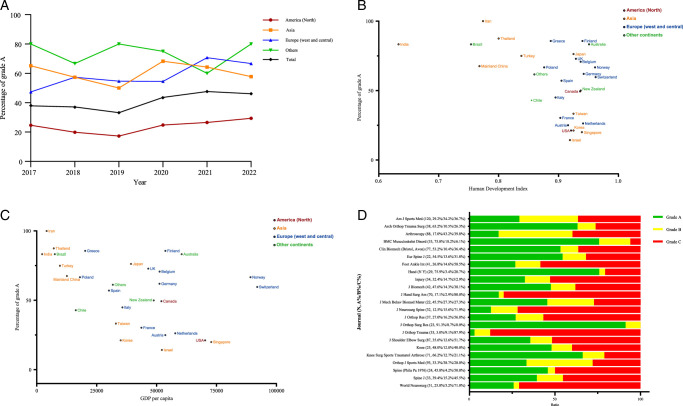
(A) Trends in percentage of grade A at the regional level between 2017 and 2022. (B) Plot of percentage of grade A of individual country or regions (published more than 5 articles) by Human Development Index (HDI). (C) Plot of percentage of grade A of individual country or regions (published more than 5 articles) by Gross Domestic Product (GDP) per capita in US$. (D) Distribution of article grades for journals (published more than 20 articles) from 2017 to 2022.

The annual number of biomechanical articles that relied on cadavers has been consistent at ~300 (Supplementary Table, Supplemental Digital Content 1, http://links.lww.com/JS9/D186). Furthermore, the quality of reported ethical data demonstrates no significant improvement over time from 2017 to 2022, as depicted in Figure 2A and Supplementary Figure 1,2, Supplemental Digital Content 2, http://links.lww.com/JS9/D187.

### Regional differences of reporting quality on ethical information

Our findings indicate that over half of the studies were conducted in North America, totaling to 979 (52.9%), followed by Western and Central Europe with 492 studies (26.6%), and Asia with 316 studies (17.1%). Figure 2B and C and Supplementary Figures 3-6, Supplemental Digital Content 2, http://links.lww.com/JS9/D187 present the distribution of articles published in grade A, B, and C in relation to the HDI and GDP per capita of the countries and regions with more than 5 articles during the study period (n=29). In multivariable analysis, we discovered that the grades of ethical approval were not correlated with country and region-related factors, including continent, HDI, GDP per capita, total population, and mortality (Table 1). Supplementary Figure 7, Supplemental Digital Content 2, http://links.lww.com/JS9/D187 provides a summary of the percentage of articles receiving grade A across the United States for states that have published more than five articles during the study period (*n*=30).

**Table 1:** Coefficient estimates in the multivariable analyses.

	Percentage of grade A		Percentage of grade C	
	β (95% CI)	*P*	β (95% CI)	*P*
Continent				
America (North)	Reference		Reference	
Europe (west and central)	18.6 (−12.3, 49.4)	0.24	−18.4 (−43.9, 7.1)	0.16
Asia	4.5 (−28.9, 37.8)	0.79	−5.7 (−33.2, 21.9)	0.69
Others	13.8 (−22.2, 49.8)	0.45	−15.0 (−44.7, 14.7)	0.32
HDI	−170.9 (−439.0, 97.2)	0.21	37.2 (−184.1, 258.4)	0.74
GDP (thousand US$)	−0.13 (−0.74, 0.48)	0.67	0.34 (−0.16, 0.85)	0.18
Total population (millions)	0.009 (−0.152, 0.170)	0.91	−0.021 (−0.154, 0.113)	0.76
Mortality (thousands)	−0.002 (−0.022, 0.018)	0.84	0.002 (−0.014, 0.018)	0.80

GDP, Gross Domestic Product; HDI, Human Development Index.

### Variation of reporting quality on ethical information by journals

Among all journals publishing biomechanical articles, *the American Journal of Sports Medicine* contributed 120 articles (6.5%), followed by *the Orthopaedic Journal of Sports Medicine* with 93 articles (5.0%), and *Arthroscopy: The Journal of Arthroscopic and Related Surgery* with 88 articles (4.8%). Figure 2D illustrates the distribution of article grades for journals that published more than 20 articles during the study period (*n*=23).

## Discussion

The Helsinki Declaration by World Medical Association is the most widely accepted ethical guidelines to protect human participants in medical research^14^. Recently, Declaration of Taipei on ethical considerations regarding health databases and biobanks also made it clear that Helsinki Declaration should be applicable in all medical research involving human samples^3^. In Taipei Declaration, the human samples were clearly defined as all samples obtained from an individual human being, living or deceased. The requirement is reasonable because the post-death fate of a body is also a part of the person’s biography. In general, we believe that studies involving cadaveric samples should follow identical ethical requirements on those involving samples from living human. Nonetheless, the proportions of Grade A had no obvious increase since the WMA Declaration of Taipei issued on October 2016 (Fig. 2).

The ethical statement regarding the use of cadaveric tissues did not surpass our expectations in high HDI countries or regions, and the current condition of ethical reporting on the use of cadaveric tissues might be concerning as it suggests that some countries with high development levels may not be following proper ethical guidelines when conducting research on cadaveric tissues. In the United States, for instance, ~150 studies on biomechanical research are conducted each year. Notably, a significant portion of specimens are obtained from for-profit body donation companies according to our study. Although commercial sources of cadaveric samples remain ethically controversial to date^7,15^, the radical notion that cadaveric specimens should be free of charge must be avoided. In our opinion, for-profit companies are ethically acceptable because accelerating biomechanical studies by providing more cadaveric samples could benefit more living patients. However, there is a basic prerequisite that the for-profit companies should be strictly regulated and supervised to ensure their loved one’s body has been treated with respect and dignity.

According to our results, the reporting quality of ethical approval varies across journals, with certain journals lacking requirements for ethical approval review. The editors-in-chief of anatomical journals have recommended a standardized statement for the ethical use of human cadaveric tissues in anatomy research papers^10^. However, this statement seems to have gone unnoticed by editors of orthopedic journals, particularly those focused on sports medicine. Given the considerable variations in legal and ethical frameworks, as well as differences in cultural and religious beliefs among countries, it would be beneficial to establish a standard set of author guidelines for orthopedic journals on biomechanical research. We hope journals, particularly those with high impact factors, to facilitate the standardization of statements regarding the ethical approval of cadaveric research.

## Conclusion

This study synthesized the publication data of biomechanical research employing cadaveric samples. We descriptively illustrated the transparency of cadaver sourcing and the acquisition of ethical approval in related articles published within the past six years. This offers interested researchers and journal readers a point of reference for discussing the ethics surrounding the utilization of cadaveric tissues in biomechanical studies.

## Ethical approval

Not applicable. No ethical approval was required as this research study didn’t involve patients.

## Consent

Not applicable.

## Source of funding

Not applicable.

## Author contribution

L.Z.: conceptualization, investigation, methodology, writing—original draft, writing—review and editing. Y.Q.: conceptualization, investigation, methodology, writing—original draft, writing—review and editing. Q.C.: resources, project administration. H.J.: formal analysis, supervision, writing—original draft, writing—review and editing. J.L.: supervision, methodology, resources, writing—review and editing. J.S.: conceptualization, methodology, project administration, resources. H.Z.: formal analysis, supervision, writing—original draft, writing—review and editing.

## Conflicts of interest disclosure

All authors declare no competing interests and confirm that authors or their institutions have not received any payments or services in the past 36 months from a third party that could be perceived to influence, or give the appearance of potentially influencing, the submitted work.

## Research registration unique identifying number (UIN)

Not applicable.

## Guarantor

Hongyi Zhu.

## Data availability statement

All relevant anonymized patient-level data are available on reasonable request to the corresponding author.

## Provenance and peer review

Not commissioned, externally peer-reviewed.

## Supplementary Material
