# Supplementary material for: Global trends and insights in ethical statements regarding the utilization of human cadaveric tissues for biomechanical research from 2017 to 2022: a bibliometric analysis
Source: Int J Surg. 2024 Jul 24;111(1):1503–7. doi: 10.1097/JS9.0000000000001986 (PMC11745705; doi:10.1097/JS9.0000000000001986)
Supplement: Supplementary file 1 [file js9-111-1503-s001.pdf]

**Supplementary Table. Comprehensive list of the included articles**

| Year | PMID | Title | Journal (Titles are abbreviated per PubMed format | Country and region | Grade |
|---|---|---|---|---|---|
| 2017 | 25091262 | Flexibility of thoracic spines under simultaneous multi-planar loading | Eur Spine J | USA | C |
| 2017 | 25813011 | Effect of augmentation techniques on the failure of pedicle screws under cranio-caudal cyclic loading | Eur Spine J | Germany | C |
| 2017 | 25894751 | Distal femoral varus osteotomy for unloading valgus knee malalignment: a biomechanical analysis | Knee Surg Sports Traumatol Arthrosc | USA | C |
| 2017 | 26231147 | Calcium phosphate cement enhances the torsional strength and stiffness of high tibial osteotomies | Knee Surg Sports Traumatol Arthrosc | USA | B |
| 2017 | 26239862 | Knee joint kinematics after dynamic intraligamentary stabilization: cadaveric study on a novel anterior cruciate ligament repair technique | Knee Surg Sports Traumatol Arthrosc | Germany | A |
| 2017 | 26377096 | Sectioning the anterolateral ligament did not increase tibiofemoral translation or rotation in an ACL-deficient cadaveric model | Knee Surg Sports Traumatol Arthrosc | Germany | A |
| 2017 | 26572634 | Evaluation of the sealing function of the acetabular labrum: an in vitro biomechanical study | Knee Surg Sports Traumatol Arthrosc | Italy | B |
| 2017 | 26820966 | Biomechanical evaluation of MPFL reconstructions: differences in dynamic contact pressure between gracilis and fascia lata graft | Knee Surg Sports Traumatol Arthrosc | Germany | A |
| 2017 | 27003836 | Biomechanical Characteristics of Pedicle Screws in Osteoporotic Vertebrae—Comparing a New Cadaver Corpectomy Model and Pure Pull-Out Testing | J Orthop Res | Germany | A |
| 2017 | 27114184 | A novel approach for custom three-dimensional printing of a zirconia root analogue implant by digital light processing | Clin Oral Implants Res | The Netherlands | C |
| 2017 | 27123747 | Effect of Annular Defects on Intradiscal Pressures in the Lumbar Spine: An in Vitro Biomechanical Study of Diskectomy and Annular Repair | J Neurol Surg A Cent Eur Neurosurg | Germany | B |
| 2017 | 27221268 | Confocal microscopy evaluation of stromal fluorescence intensity after standard and accelerated iontophoresisassisted corneal cross-linking | Int Ophthalmol | Italy | A |
| 2017 | 27279527 | Biomechanical Consequences of Adding Plantar Fascia Release to Metatarsal Osteotomies: Changes in Forefoot Plantar Pressures | J Orthop Res | USA | C |
| 2017 | 27337240 | Biomechanical Changes in Disc Pressure and Facet Strain after Lumbar Spinal Arthroplasty with Charité™ in the Human Cadaveric Spine under Physiologic Compressive Follower Preload | Turk Neurosurg | Korea | A |
| 2017 | 27356009 | Biomechanical Study of Distal Radioulnar Joint Ballottement Test | J Orthop Res | Japan | C |
| 2017 | 27369298 | Are We Subluxating Knees in Total Knee Arthroplasty? A Cadaveric Investigation | J Arthroplasty | Germany | C |
| 2017 | 27436194 | Balancing mobile-bearing unicondylar knee arthroplasty in vitro | Knee Surg Sports Traumatol Arthrosc | Belgium | A |
| 2017 | 27499518 | Biomechanical evaluation of two arthroscopic techniques for biceps tenodesis: triple loop suture versus simple suture | J Shoulder Elbow Surg | Israel | B |
| 2017 | 27509454 | Contribution of Neural Elements to Thoracic Stability | Turk Neurosurg | Turkey | B |
| 2017 | 27514943 | Knotless Suture Anchor With Suture Tape Quadriceps Tendon Repair Is Biomechanically Superior to Transosseous and Traditional Suture AnchoreBased Repairs in a Cadaveric Model | Arthroscopy | USA | B |
| 2017 | 27554673 | Dynamic Response and Residual Helmet Liner Crush Using Cadaver Heads and Standard Headforms | Ann Biomed Eng | Canada | A |
| 2017 | 27564231 | Biomechanical Comparison of Augmented versus Non-Augmented Sacroiliac Screws in a new Hemi-Pelvis Test Model | J Orthop Res | Germany | C |
| 2017 | 27572633 | Quantification of change in vocal fold tissue stiffness relative to depth of artificial damage | Logoped Phoniatr Vocol | Germany | A |
| 2017 | 27587740 | Biomechanical Analysis of Intra-articular Pressure After Coracoclavicular Reconstruction | Am J Sports Med | Germany | C |
| 2017 | 27592329 | Tibial component with and without stem extension in a trabecular metal cone construct | Knee Surg Sports Traumatol Arthrosc | The Netherlands | B |
| 2017 | 27599822 | The Hip-Spine Effect: A Biomechanical Study of Ischiofemoral Impingement Effect on Lumbar Facet Joints | Arthroscopy | USA | C |
| 2017 | 27604514 | Flexor Hallucis Longus Tendon Transfer Fixation | Foot Ankle Spec | USA | A |
| 2017 | 27637854 | Biomechanical comparison of graft structures in anterior cruciate ligament reconstruction | Knee Surg Sports Traumatol Arthrosc | UK | A |
| 2017 | 27659939 | Hip Capsular Closure A Biomechanical Analysis of Failure Torque | Am J Sports Med | USA | C |
| 2017 | 27661732 | Biomechanical Comparison of Superior Versus Anterior Plate Position for Fixation of Distal Clavicular Fractures: A New Model | J Orthop Trauma | USA | C |
| 2017 | 27671280 | In vitro analysis of circumferential joint replacement, including bilateral facet joint replacement with lateral lumber disc prosthesis: a parametric investigation of disc sizing | Eur Spine J | USA | A |
| 2017 | 27720302 | Contribution of the Pubofemoral Ligament to Hip Stability: A Biomechanical Study | Arthroscopy | USA | C |
| 2017 | 27720412 | Suture spanning augmentation of single-row rotator cuff repair: a biomechanical analysis | J Shoulder Elbow Surg | USA | C |
| 2017 | 27720559 | Humeral head osteotomy in shoulder arthroplasty: a comparison between anterosuperior and inferoanterior resection techniques | J Shoulder Elbow Surg | USA | B |
| 2017 | 27722769 | Cadaveric study of the secondary medial patellar restraints: patellotibial and patellomeniscal ligaments | Knee Surg Sports Traumatol Arthrosc | Brazil | A |
| 2017 | 27727059 | Radiocapitellar contact characteristics during prosthetic radial head subluxation | J Shoulder Elbow Surg | USA | A |
| 2017 | 27733556 | A 3-D CT Analysis of Screw and Suture-Button Fixation of the Syndesmosis | Foot Ankle Int | USA | C |
| 2017 | 27739941 | Biomechanical evaluation of the craniovertebral junction after unilateral joint-sparing condylectomy: implications for the far lateral approach revisited | J Neurosurg | USA | C |
| 2017 | 27755333 | Biomechanical Evaluation of the Femoral Neck System in Unstable Pauwels III Femoral Neck Fractures: A Comparison with the Dynamic Hip Screw and Cannulated Screws | J Orthop Trauma | Switzerland | C |
| 2017 | 27761624 | Comparison of the efficiency of an extra-articular absorber system and high tibial osteotomy for unloading the medial knee compartment: an in vitro study | Knee Surg Sports Traumatol Arthrosc | Germany | A |
| 2017 | 27780285 | Biomechanical Comparison of Five Posterior Cruciate Ligament Reconstruction Techniques | J Knee Surg | USA | C |
| 2017 | 27830891 | Morphometric multislice computed tomography examination of the craniovertebral junction in neck flexion and extension | Folia Morphol (Warsz) | Serbia | A |
| 2017 | 27837037 | Comparison of Locking Plate with Interfragmentary Screw Versus Plantarly Applied Anatomic Locking Plate for Lapidus Arthrodesis A Biomechanical Cadaveric Study | Foot Ankle Spec | USA | C |
| 2017 | 27859544 | Lateral soft-tissue structures contribute to cruciate-retaining total knee arthroplasty stability | J Orthop Res | UK | A |
| 2017 | 27881831 | The Effect of Suture Anchor Insertion Angle on Calcaneus Pullout Strength Challenging the Deadman's Angle | Foot Ankle Spec | USA | C |
| 2017 | 27886018 | Radiofrequency-activated PMMA-augmentation through cannulated pedicle screws: A cadaver study to determine the biomechanical benefits in the osteoporotic spine | Technol Health Care | Germany | B |
| 2017 | 27887872 | Optimizing the rehabilitation of elbow lateral collateral ligament injuries: a biomechanical study | J Shoulder Elbow Surg | Canada | B |
| 2017 | 27925638 | Biomechanical Analysis of Intervertebral Cement Extravasation in Vertebral Motion Segments | Orthopedics | USA | B |
| 2017 | 27932332 | The Anterolateral Capsule of the Knee Behaves Like a Sheet of Fibrous Tissue | Am J Sports Med | USA | A |
| 2017 | 27939953 | Delineation of the mechanisms of tendon gliding resistance within the carpal tunnel | Clin Biomech (Bristol, Avon) | USA | A |
| 2017 | 27955813 | Gap formation following primary repair of the anterior cruciate ligament: A biomechanical evaluation | Knee | USA | C |
| 2017 | 27956021 | Prediction of the drilling path to surgically pin the femoral neck from the spatial location of pelvic and femoral anatomical landmarks: A cadaver validation study | Med Eng Phys | Belgium | A |
| 2017 | 27984448 | Novel Spiked-Washer Repair Is Biomechanically Superior to Suture and Bone Tunnels for Arcuate Fracture Repair | J Orthop Trauma | USA | C |
| 2017 | 27993094 | Talonavicular ligament: prevalence of injury in ankle sprains, histological analysis and hypothesis of its biomechanical function. | Br J Radiol | UK | A |
| 2017 | 27997465 | Does Lumbopelvic Fixation Add Stability? A Cadaveric Biomechanical Analysis of an Unstable Pelvic Fracture Model | J Orthop Trauma | USA | C |
| 2017 | 28002219 | Do Transcortical Screws in a Locking Plate Construct Improve the Stiffness in the Fixation of Vancouver B1 Periprosthetic Femur Fractures? A Biomechanical Analysis of 2 Different Plating Constructs | J Orthop Trauma | Canada | C |
| 2017 | 28005166 | Anatomic dissection of the anterolateral ligament (ALL) in paired fresh-frozen cadaveric knee joints | Arch Orthop Trauma Surg | Germany | A |
| 2017 | 28005168 | PEEK versus titanium locking plates for proximal humerus fracture fixation: a comparative biomechanical study in twoand three-part fractures | Arch Orthop Trauma Surg | Germany | A |
| 2017 | 28011034 | Interosseous Ligament and Transverse Forearm Stability: A Biomechanical Cadaver Study | J Hand Surg Am | USA | A |
| 2017 | 28027384 | An Anatomical Study on the Safe Placement of Orthopedic Hardware for Syndesmosis Fixation | Orthopedics | USA | B |
| 2017 | 28027653 | Biomechanical Comparison of Anterolateral Procedures Combined With Anterior Cruciate Ligament Reconstruction | Am J Sports Med | UK | A |
| 2017 | 28029798 | Should the Ipsilateral Hamstrings Be Used for Anterior Cruciate Ligament Reconstruction in the Case of Medial Collateral Ligament Insufficiency | Am J Sports Med | Germany | B |

| | | | | | |
|---|---|---|---|---|---|
| 2017 | 28034486 | Biomechanical Comparison of Open and Arthroscopic Transosseous Repair of Triangular Fibrocartilage Complex Foveal Tears: A Cadaveric Study | Arthroscopy | Taiwan | B |
| 2017 | 28035443 | Effect of mesh width on apical support after sacrocolpopexy | Int Urogynecol J | USA | B |
| 2017 | 28038688 | Biomechanical Evaluation of a Growth-Friendly Rod Construct | Spine Deform | USA | C |
| 2017 | 28052832 | Fixation of Regan-Morrey Type II Coronoid Fractures: A Comparison of Screws and Suture Lasso Technique for Resistance to Displacement | J Hand Surg Am | USA | C |
| 2017 | 28052834 | Efficacy of Dorsoradial Capsulodesis for Trapeziometacarpal Joint Instability: A Cadaver Study | J Hand Surg Am | USA | C |
| 2017 | 28057631 | Ultimate fracture load of cadaver proximal humeri correlates more strongly with mean combined cortical thickness than with areal cortical index, DEXA density, or canal-to-calcar ratio | Bone Joint Res | USA | A |
| 2017 | 28057632 | Are two retrograde 3.5 mm screws superior to one 7.3 mm screw for anterior pelvic ring fixation in bones with low bone mineral density? | Bone Joint Res | Switzerland | C |
| 2017 | 28062120 | Robotic simulation of identical athletic-task kinematics on cadaveric limbs exhibits a lack of differences in knee mechanics between contralateral pairs | J Biomech | USA | B |
| 2017 | 28065623 | An anatomic investigation into the relationship between posterior condylar offset and posterior tibial slope of 1,138 cadaveric knees | J Arthroplasty | USA | B |
| 2017 | 28078394 | Kinematics of a bicruciate-retaining total knee arthroplasty | Knee Surg Sports Traumatol Arthrosc | Belgium | A |
| 2017 | 28079730 | Biomechanical Comparison of Cadaveric and Commercially Available Synthetic Osteoporotic Bone Analogues in a Locked Plate Fracture Model Under Torsional Loading | J Orthop Trauma | USA | C |
| 2017 | 28086149 | Shear waves elastography for assessment of human Achilles tendon’s biomechanical properties: an experimental study | J Mech Behav Biomed Mater | France | B |
| 2017 | 28089497 | Biomechanical Comparison of Standard and Linked Single-Row Rotator Cuff Repairs in a Human Cadaver Model | Arthroscopy | USA | B |
| 2017 | 28091968 | Load-Based Lower Neck Injury Criteria for Females from Rear Impact from Cadaver Experiments | Ann Biomed Eng | USA | C |
| 2017 | 28107242 | An In-Vitro Biomechanical Study Evaluating Cervical Extension Plates for Stabilizing Degenerated Adjacent Levels | Clin Spine Surg | USA | C |
| 2017 | 28108780 | Cement-augmented screws in a cervical two-level corpectomy with anterior titanium mesh cage reconstruction: a biomechanical study | Eur Spine J | Austria | A |
| 2017 | 28116949 | The effect of in-situ decompression on ulnar nerve stability: a cadaveric study | J Hand Surg Eur Vol | USA | C |
| 2017 | 28117253 | Comparison of Four Methods for Percutaneous Achilles Tendon Lengthening: A Cadaveric Study | J Foot Ankle Surg | China | B |
| 2017 | 28124108 | The infra-meniscal fibers of the anterolateral ligament are stronger and stiffer than the supra-meniscal fibers despite similar histological characteristics | Knee Surg Sports Traumatol Arthrosc | Canada | A |
| 2017 | 28128700 | Biomechanical evaluation of an integrated fixation cage during fatigue loading: a human cadaver study | J Neurosurg Spine | USA | B |
| 2017 | 28129269 | The Effect of Varying Tension of a Suture Button Construct in Fixation of the Tibiofibular Syndesmosis—Evaluation Using Stress Computed Tomography | J Orthop Trauma | USA | C |
| 2017 | 28129271 | Fatigue Failure in Extra-Articular Proximal Tibia Fractures: Locking Intramedullary Nail Versus Double Locking Plates— A Biomechanical Study | J Orthop Trauma | USA | B |
| 2017 | 28131690 | Biomechanical comparison of acute Hill-Sachs reduction with remplissage to treat complex anterior instability | J Shoulder Elbow Surg | USA | C |
| 2017 | 28131691 | Scapula fracture incidence in reverse total shoulder arthroplasty using screws above or below metaglene central cage: clinical and biomechanical outcomes | J Shoulder Elbow Surg | USA | A |
| 2017 | 28146029 | Kinematics of the cervical spine following unilateral facet fracture: an in-vitro cadaver study | Spine (Phila Pa 1976) | Italy | C |
| 2017 | 28159312 | Effects of Anterior Offsetting of Humeral Head Component in Posteriorly Unstable Total Shoulder Arthroplasty: Finite Element Modeling of Cadaver Specimens | J Biomech | USA | C |
| 2017 | 28165760 | Comparison of ACL Strain in the MCL-Deficient and MCL-Reconstructed Knee During Simulated Landing in a Cadaveric Model | Am J Sports Med | USA | B |
| 2017 | 28166805 | Weight-bearing recommendations after first metatarsophalangeal joint arthrodesis fixation: a biomechanical comparison | J Orthop Surg Res | USA | A |
| 2017 | 28167249 | How are adjacent spinal levels affected by vertebral fracture and by vertebroplasty? A biomechanical study on cadaveric spines | Spine J | UK | C |
| 2017 | 28185776 | Tibial tubercle osteotomy: A biomechanical comparison of two techniques | Knee | USA | B |
| 2017 | 28185981 | Analysis of the s2 alar-iliac screw as compared with the traditional iliac screw: does it increase stability with sacroiliac fixation of the spine? | Spine J | USA | B |
| 2017 | 28188484 | Estimation of attachment regions of hip muscles in CT image using muscle attachment probabilistic atlas constructed from measurements in eight cadavers | Int J Comput Assist Radiol Surg | Japan | A |
| 2017 | 28193295 | Biomechanical comparison of single- and double-bundle medial patellofemoral ligament reconstruction | J Orthop Surg Res | China | A |
| 2017 | 28194498 | Lateral clavicle fracture with coracoclavicular ligament injury: a biomechanical study of 4 different repair techniques | Knee Surg Sports Traumatol Arthrosc | USA | C |
| 2017 | 28195745 | Anatomic and Biomechanical Comparison of the Classic and Congruent-Arc Techniques of the Latarjet Procedure | Am J Sports Med | USA | C |
| 2017 | 28197694 | Relaxation of the MCL after an Open-Wedge High Tibial Osteotomy results in decreasing contact pressures of the knee over time | Knee Surg Sports Traumatol Arthrosc | The Netherlands | A |
| 2017 | 28199931 | A personalized 3D-printed prosthetic joint replacement for the human temporomandibular joint: from implant design to implantation | J Mech Behav Biomed Mater | Australia | C |
| 2017 | 28204962 | The clinical anatomy of the insertion of the rotator cuff tendons | Eur J Orthop Surg Traumatol | South Africa | A |
| 2017 | 28205465 | Biomechanics of Internal Fixation Modalities for Middle Phalangeal Base Fracture Dislocation | J Hand Surg Asian Pac Vol | Singapore | A |
| 2017 | 28205540 | Anatomical Characteristics and Biomechanical Properties of the Oblique Popliteal Ligament | Sci Rep | China | A |
| 2017 | 28209401 | The Effect of Prophylactic Cerclage Wires in Primary Total Hip Arthroplasty: A Biomechanical Study | J Arthroplasty | USA | C |
| 2017 | 28222170 | Biomechanical investigation of two plating systems for medial column fusion in foot | PLoS One | Germany | C |
| 2017 | 28235698 | The effect of anterior longitudinal ligament resection on lordosis correction during minimally invasive lateral lumbar interbody fusion: Biomechanical and radiographic feasibility of an integrated spacer/plate | Clin Biomech (Bristol, Avon) | USA | C |
| 2017 | 28239827 | Quantitative difference of acute intraoperative expansion in various body regions | Eur Rev Med Pharmacol Sci | Italy | C |
| 2017 | 28241752 | Distal radius plate of CFR-PEEK has minimal effect compared to titanium plates on bone parameters in high-resolution peripheral quantitative computed tomography: a pilot study | BMC Med Imaging | The Netherlands | B |
| 2017 | 28243363 | Novel Patient-Specific Drill Guide Template for Pedicle Screw Insertion into the Subaxial Cervical Spine Utilizing Stereolithographic Modelling: An In Vitro Study | Asian Spine J | Philippines | B |
| 2017 | 28247079 | Two-piece ALIF cage optimizes the bone-implant interface in a 360° setting | Eur Spine J | Germany | A |
| 2017 | 28256384 | Osteosynthesis using cannulated headless Herbert screws in mandibular angle fracture treatment: A new approach | J Craniomaxillofac Surg | Austria | A |
| 2017 | 28258329 | Function and strain of the anterolateral ligament part I: biomechanical analysis | Knee Surg Sports Traumatol Arthrosc | Germany | A |
| 2017 | 28259271 | Influence of Sequential Ponte Osteotomies on the Human Thoracic Spine | Spine Deform | USA | C |
| 2017 | 28259283 | Impact of Distal Ulnar Fracture Malunion on Distal Radioulnar Joint Instability: A Biomechanical Study of the Distal Interosseous Membrane Using a Cadaver Model | J Hand Surg Am | Japan | C |
| 2017 | 28259613 | Biplane fluoroscopy for hindfoot motion analysis during gait: A model-based evaluation | Med Eng Phys | USA | A |
| 2017 | 28260125 | Pull-out strength of patient-specific template-guided vs. free-hand fluoroscopically controlled thoracolumbar pedicle screws: a biomechanical analysis of a randomized cadaveric study | Eur Spine J | Switzerland | A |
| 2017 | 28262985 | Smith's fracture generally occurs after falling on the palm of the hand | J Orthop Res | Japan | A |
| 2017 | 28278314 | Biomechanic Factors Associated With Orbital Floor Fractures | JAMA Facial Plast Surg | USA | B |
| 2017 | 28284491 | Hexagonal grafts in mosaicplasty: Biomechanical comparison of standard cylindrical and novel hexagonal grafts in calf cadaver model | Acta Orthop Traumatol Turc | Turkey | C |
| 2017 | 28296717 | Abdominal Wall Allograft Preclinical Biomechanical Investigation of a Novel Reconstructive Adjunct | Ann Plast Surg | USA | A |
| 2017 | 28297604 | Biomechanical Evaluation of Classic Solid and All-Soft Suture Anchors for Medial Patellofemoral Ligament Reconstruction | Am J Sports Med | USA | B |
| 2017 | 28298142 | Effect of Complete Syndesmotic Disruption and Deltoid Injuries and Different Reduction Methods on Ankle Joint Contact Mechanics | Foot Ankle Int | Canada | A |
| 2017 | 28314887 | Mechanical strains passing through the acetabular labrum modify its shape during hip motion: an anatomical study | Knee Surg Sports Traumatol Arthrosc | France | B |
| 2017 | 28314890 | Diagnostic findings caused by cutting of the iliotibial tract and anterolateral ligament in an ACL intact knee using a standardized and automated clinical knee examination | Knee Surg Sports Traumatol Arthrosc | USA | B |
| 2017 | 28319734 | Novel mechanical impact simulator designed to generate clinically relevant anterior cruciate ligament ruptures | Clin Biomech (Bristol, Avon) | USA | A |
| 2017 | 28321426 | Quantitative Anatomic Analysis of the Native Ligamentum Teres | Orthop J Sports Med | USA | B |
| 2017 | 28321432 | A Novel Implant System for Unloading the Medial Compartment of the Knee by Lateral Displacement of the Iliotibial Band | Orthop J Sports Med | USA | A |

| | | | | | |
|---|---|---|---|---|---|
| 2017 | 28321479 | Novel anatomical reconstruction of distal tibiofibular ligaments restores syndesmotic biomechanics | Knee Surg Sports Traumatol Arthrosc | China | C |
| 2017 | 28323239 | Biomechanical analysis of lateral interbody fusion strategies for adjacent segment degeneration in the lumbar spine | Spine J | USA | C |
| 2017 | 28323703 | Biomechanical Effects of Human Lumbar Discography: In-vitro experiments and their finite element validation | Clin Spine Surg | USA | C |
| 2017 | 28323708 | Fenestrated screws augmented with PMMA increase the pull-out strength of sacral pedicle screws | Clin Spine Surg | USA | C |
| 2017 | 28323715 | The safety and strength of a novel medial, partial non-threaded pedicle screw: A cadaveric and biomechanical investigation | Clin Spine Surg | Malaysia | A |
| 2017 | 28330756 | Femoral articular geometry and patellofemoral stability | Knee | Malaysia | A |
| 2017 | 28332149 | Biomechanical cadaveric comparison of patellar ligament suture protected by a steel cable versus a synthetic cable | J Exp Orthop | France | A |
| 2017 | 28334406 | Male and female cervical spine biomechanics and anatomy: Implication for scaling injury criteria | J Biomech Eng | USA | C |
| 2017 | 28335824 | The biomechanical and histological effects of posterior cruciate ligament rupture on the medial tibial plateau | J Orthop Surg Res | China | A |
| 2017 | 28338579 | Correlative analysis of vertebral trabecular bone microarchitecture and mechanical properties: a combined ultra-high field (7 Tesla) MRI and biomechanical investigation | Spine (Phila Pa 1976) | France | A |
| 2017 | 28342975 | Biomechanical testing of a PEEK-based dynamic instrumentation device in a lumbar spine model | Clin Biomech (Bristol, Avon) | Germany | A |
| 2017 | 28359696 | Partial-thickness tears involving the rotator cable lead to abnormal glenohumeral kinematics | J Shoulder Elbow Surg | USA | B |
| 2017 | 28359697 | A comparison of onlay versus inlay glenoid component loosening in total shoulder arthroplasty | J Shoulder Elbow Surg | USA | B |
| 2017 | 28361277 | The effect of a dynamic PCL brace on patellofemoral compartment pressures in PCL-and PCL/PLC-deficient knees | J Exp Orthop | USA | B |
| 2017 | 28362894 | Occipitocervical Instability After Far-Lateral Transcondylar Surgery: A Biomechanical Analysis | Neurosurgery | USA | A |
| 2017 | 28367688 | First Metatarsophalangeal Contact Properties Following Proximal Opening Wedge and Scarf Osteotomies for Hallux Valgus Correction: A Biomechanical Study | Foot Ankle Int | USA | C |
| 2017 | 28371596 | Biomechanical Assessment of Hip Capsular Repair and Reconstruction Procedures Using a 6 Degrees of Freedom Robotic System | Am J Sports Med | USA | B |
| 2017 | 28374092 | A cadaveric biomechanical study comparing the ease of femoral nail insertion: 1.0- vs 1.5-m bow designs | Arch Orthop Trauma Surg | Australia | A |
| 2017 | 28377865 | Regional Variations in Shear Strength and Density of the Human Thoracic Vertebral Endplate and Trabecular Bone | Int J Spine Surg | USA | A |
| 2017 | 28384527 | Anterolateral ligament reconstruction with autologous grafting: a biomechanical study | Clin Biomech (Bristol, Avon) | Italy | A |
| 2017 | 28386725 | Biomechanical properties of posterior transpedicular–transdiscal oblique lumbar screw fixation with novel trapezoidal lateral interbody spacer: an in vitro human cadaveric model | Eur Spine J | USA | A |
| 2017 | 28387161 | Biomechanical Assessment of the Dorsal Spanning Bridge Plate in Distal Radius Fracture Fixation: Implications for Immediate Weight-Bearing | Hand (N Y) | USA | A |
| 2017 | 28390687 | Overtightening of the Syndesmosis Revisited and the Effect of Syndesmotic Malreduction on Ankle Dorsiflexion | Injury | USA | C |
| 2017 | 28399553 | Rectus Capitis Posterior Minor Histological and Biomechanical Links to the Spinal Dura Mater | Spine (Phila Pa 1976) | Canada | A |
| 2017 | 28402759 | Medial Posterior Capsular Plication Reduces Anterior Shoulder Instability Similar to Remplissage Without Restricting Motion in the Setting of an Engaging Hill-Sachs Defect | Am J Sports Med | USA | B |
| 2017 | 28405219 | Braided tape is equivalent to modified Mason-Allen multi-strand #2 suture in subscapularis muscle repair: results of a biomechanical study | Shoulder Elbow | Canada | A |
| 2017 | 28411549 | Stability of knee ligament complex of Thiel-embalmed cadaver compared to in vivo knee | J Mech Behav Biomed Mater | Germany | A |
| 2017 | 28422889 | An in vitro biomechanical evaluation of an expansive double-threaded bi-directional compression screw for fixation of type II odontoid process fractures A SQUIRE-compliant article | Medicine (Baltimore) | China | A |
| 2017 | 28423932 | Comparing Biomechanical Properties, Repair Times, and Value of Common Core Flexor Tendon Repairs | Hand (N Y) | USA | A |
| 2017 | 28424860 | Influence of tibial hybrid fixation on graft tension and stability in ACL double-bundle reconstruction | Arch Orthop Trauma Surg | Germany | B |
| 2017 | 28428912 | Biomechanical Evaluation of Carpal Kinematics during Simulated Wrist Motion | J Wrist Surg | Canada | C |
| 2017 | 28433348 | The influence of polyethylene bearing thickness on the tibiofemoral kinematics of a bicruciate retaining total knee arthroplasty | Knee | Belgium | A |
| 2017 | 28444241 | Cervical Spine Muscle-Tendon Unit Length Differences Between Neutral and Forward Head Postures: Biomechanical Study Using Human Cadaveric Specimens | Phys Ther | USA | C |
| 2017 | 28451509 | Hybrid Instrumentation in Lumbar Spinal Fusion: A Biomechanical Evaluation of Three Different Instrumentation Techniques | Global Spine J | Germany | C |
| 2017 | 28451858 | Biomechanical in vitro comparison of radiofrequency kyphoplasty and balloon kyphoplasty | Eur Spine J | Germany | C |
| 2017 | 28453347 | Drill Penetration Injury to Extensor Tendons: A Biomechanical Analysis | Hand (N Y) | USA | A |
| 2017 | 28455730 | Knee Abduction Affects Greater Magnitude of Change in ACL and MCL Strains Than Matched Internal Tibial Rotation In Vitro | Clin Orthop Relat Res | USA | A |
| 2017 | 28458187 | Torsional stiffness after subtalar arthrodesis using second generation headless compression screws: Biomechanical comparison of 2-screw and 3screw fixation | Clin Biomech (Bristol, Avon) | Austria | C |
| 2017 | 28458472 | Plating of pilon fractures based on the orientation of the fibular shaft component: A biomechanical study evaluating plate stiffness in a cadaveric fracture model | J Orthop | USA | C |
| 2017 | 28460573 | Biomechanical Analysis of Suture Anchor vs Tenodesis Screw for FHL Transfer | Foot Ankle Int | USA | C |
| 2017 | 28472018 | Does Spanning a Lateral Lumbar Interbody Cage Across the Vertebral Ring Apophysis Increase Loads Required for Failure and Mitigate Endplate Violation | Spine (Phila Pa 1976) | USA | C |
| 2017 | 28476383 | Evaluation of a New Knotless Suture Anchor Repair in Acute Achilles Tendon Ruptures: A Biomechanical Comparison of Three Techniques | J Foot Ankle Surg | USA | C |
| 2017 | 28478734 | Development and validation of a subject-specific finite element model of the functional spinal unit to predict vertebral strength | Proc Inst Mech Eng H | UK | A |
| 2017 | 28482680 | Optimal Starting Point for Fifth Metatarsal Zone II Fractures: A Cadaveric Study | Foot Ankle Int | USA | A |
| 2017 | 28497049 | Biomechanical Analysis of Cuboid Osteotomy Lateral Column Lengthening for Stage II B Adult-Acquired Flatfoot Deformity: A Cadaveric Study | Biomed Res Int | China | A |
| 2017 | 28497166 | Biomechanical evaluation of straight antegrade nailing in proximal humeral fractures: the rationale of the Bproximal anchoring point^ | Int Orthop | USA | B |
| 2017 | 28498072 | Paravertebral foramen screw fixation for posterior cervical spine fusion: biomechanical study and description of a novel technique | J Neurosurg Spine | Japan | B |
| 2017 | 28499921 | Anterior Cruciate Ligament Soft Tissue Graft Fixation in the Elderly: Is There a Reason to Use Interference Screws? A Human Cadaver Study | Arthroscopy | Germany | C |
| 2017 | 28501956 | An in vitro evaluation of sagittal alignment in the cervical spine after insertion of supraphysiologic lordotic implants | Eur Spine J | USA | C |
| 2017 | 28520458 | Anchored Transosseous-Equivalent Versus Anchorless Transosseous Rotator Cuff Repair A Biomechanical Analysis in a Cadaveric Model | Am J Sports Med | USA | B |
| 2017 | 28525468 | Cortical Bone Trajectory for Thoracic Pedicle Screws: A Technical Note | Clin Spine Surg | Japan | A |
| 2017 | 28525470 | Factors Affecting the Non-Linear Force Versus Distraction Height Curves in an In Vitro C5–6 Anterior Cervical Distraction Model | Clin Spine Surg | China | C |
| 2017 | 28525483 | Development of a Remodeled Caspar Retractor and Its Application in the Measurement of Distractive Resistance in an In Vitro Anterior Cervical Distraction Model | Clin Spine Surg | China | A |
| 2017 | 28536855 | Rotational Laxity Control by the Anterolateral Ligament and the Lateral Meniscus Is Dependent on Knee Flexion Angle: A Cadaveric Biomechanical Study | Clin Orthop Relat Res | Canada | B |
| 2017 | 28545494 | No correlation between radiolucency and biomechanical stability of keeled and pegged glenoid components | BMC Musculoskelet Disord | USA | C |
| 2017 | 28549397 | Assessment of Distal Radioulnar Joint Stability After Reconstruction With the Brachioradialis Wrap | Hand (N Y) | USA | A |
| 2017 | 28554053 | Radiographic evaluation of acute distal radius fracture stability: A comparative cadaveric study between a thermo-formable bracing system and traditional fiberglass casting | Clin Biomech (Bristol, Avon) | USA | B |
| 2017 | 28554845 | A novel anatomical patellar plate for transverse patellar fracture e A biomechanical in-vitro study | Acta Orthop Traumatol Turc | Turkey | A |
| 2017 | 28559102 | Biomechanical characteristics of the anatomic rectangular tunnel anterior cruciate ligament reconstruction with a bone-patellar tendon-bone graft | J Orthop Sci | Japan | A |
| 2017 | 28585023 | Dynamic radiostereometric analysis for evaluation of hip joint pathomechanics | J Exp Orthop | Denmark | A |
| 2017 | 28591075 | Comparison of the Vacuum Mattress versus the Spine Board Alone for Immobilization of the Cervical Spine Injured Patient: A Biomechanical Cadaveric Study | Spine (Phila Pa 1976) | USA | C |
| 2017 | 28597401 | Biomechanical evaluation of the risk of secondary fracture around short versus long cephalomedullary nails | Eur J Orthop Surg Traumatol | USA | C |
| 2017 | 28601513 | A Biomechanical Comparison of Distal Fixation for Bridge Plating in a Distal Radius Fracture Model | J Hand Surg Am | USA | C |
| 2017 | 28604653 | Effects of Titanium Mesh Cage End Structures on the Compressive Load at the Endplate Interface: A Cadaveric Biomechanical Study | Med Sci Monit | China | A |

| | | | | | |
|---|---|---|---|---|---|
| 2017 | 28607938 | Biomechanical Head-to-Head Comparison of 2 Sutures and the Giftbox Versus Bunnell Techniques for Midsubstance Achilles Tendon Ruptures | Orthop J Sports Med | USA | A |
| 2017 | 28607939 | The Role of the Peripheral Passive Rotation Stabilizers of the Knee With Intact Collateral and Cruciate Ligaments | Orthop J Sports Med | USA | B |
| 2017 | 28607942 | A Comparative Biomechanical Analysis of 2 Double-Row, Distal Triceps Tendon Repairs | Orthop J Sports Med | USA | A |
| 2017 | 28608580 | Pedicle screw augmentation with bone cement enforced Vicryl mesh | J Orthop Res | Switzerland | A |
| 2017 | 28609131 | A Comprehensive Reanalysis of the Distal Iliotibial Band Quantitative Anatomy, Radiographic Markers, and Biomechanical Properties | Am J Sports Med | USA | C |
| 2017 | 28616758 | Does Humeral Component Lateralization in Reverse Shoulder Arthroplasty Affect Rotator Cuff Torque? Evaluation in a Cadaver Model | Clin Orthop Relat Res | Canada | C |
| 2017 | 28616759 | Is Dual Semitendinosus Allograft Stronger Than Turndown for Achilles Tendon Reconstruction? An In Vitro Analysis | Clin Orthop Relat Res | USA | C |
| 2017 | 28622409 | Ultrasound-guided hydrodissection decreases gliding resistance of the median nerve within the carpal tunnel | Muscle Nerve | USA | A |
| 2017 | 28622833 | Biomechanical evaluation of the tension band wiring principle. A comparison between two different techniques for transverse patella fracture fixation | Injury | Switzerland | C |
| 2017 | 28628800 | Increased internal femoral torsion can be regarded as a risk factor for patellar instability — A biomechanical study | Clin Biomech (Bristol, Avon) | Austria | C |
| 2017 | 28632549 | Biomechanical Investigation of a Novel Revision Device in an Osteoporotic Model | Clin Spine Surg | USA | C |
| 2017 | 28633783 | A Biomechanical Analysis of Interference Screw Versus Bone Tunnel Fixation of Flexor Hallucis Longus Tendon Transfers to the Calcaneus | J Foot Ankle Surg | USA | B |
| 2017 | 28634743 | Comminuted olecranon fractures: biomechanical testing of locked versus minifragment non-locked plate fixation | Arch Orthop Trauma Surg | USA | C |
| 2017 | 28645703 | Mapping of the anterior tibial profile to identify accurate reference points for sagittal alignment of tibial component in total knee arthroplasty | Orthop Traumatol Surg Res | Italy | C |
| 2017 | 28660106 | Biomechanical Evaluation of Unilateral Versus Bilateral C1 Lateral Mass-C2 Intralaminar Fixation | Global Spine J | USA | B |
| 2017 | 28660229 | Lateral Meniscus Posterior Root and Meniscofemoral Ligaments as Stabilizing Structures in the ACL-Deficient Knee A Biomechanical Study | Orthop J Sports Med | USA | B |
| 2017 | 28662894 | Medial Patellofemoral Ligament, Medial Patellotibial Ligament, and Medial Patellomeniscal Ligament: Anatomic, Histologic, Radiographic, and Biomechanical Study | Arthroscopy | Brazil | A |
| 2017 | 28669547 | The effects of wrist motion and hand orientation on muscle forces: A physiologic wrist simulator study | J Biomech | UK | A |
| 2017 | 28671877 | Single-screw Fixation of Adolescent Salter-II Proximal Humeral Fractures: Biomechanical Analysis of the “One Pass Door Lock” Technique | J Pediatr Orthop | USA | C |
| 2017 | 28684171 | Biomechanical tensile strength analysis for medial patellofemoral ligament reconstruction | Knee | USA | C |
| 2017 | 28688826 | Biomechanical Analysis of Latarjet Screw Fixation: Comparison of Screw Types and Fixation Methods | Arthroscopy | USA | A |
| 2017 | 28689825 | Biomechanical analysis of anterior bone graft augmentation with reversed shoulder arthroplasty in large combined glenoid defects compared with total bony joint line reconstruction (modified bony-increase | J Shoulder Elbow Surg | Germany | A |
| 2017 | 28707122 | Segond's fracture: a biomechanical cadaveric study using navigation | J Orthop Traumatol | Italy | B |
| 2017 | 28707505 | Biomechanical analysis of the thoracolumbar spine under physiological loadings: Experimental motion data corridors for validation of finite element models | Proc Inst Mech Eng H | France | C |
| 2017 | 28709795 | Biomechanical Comparison of Extensor Carpi Ulnaris Subsheath Reconstruction Techniques | J Hand Surg Am | USA | C |
| 2017 | 28711411 | Efficacy of a radial-based thumb metacarpophalangeal-stabilizing orthosis for protecting the thumb metacarpophalangeal joint ulnar collateral ligament | J Hand Ther | USA | C |
| 2017 | 28717613 | Morphometric evaluation of human tendocalcaneus: a cadaveric study of south indian male population | Muscles Ligaments Tendons J | India | A |
| 2017 | 28725508 | The Anatomy of the Dorsal CapsuloScapholunate Septum: A Cadaveric Study | J Wrist Surg | France | C |
| 2017 | 28734719 | Coronoid reconstruction using osteochondral grafts: a biomechanical study | J Shoulder Elbow Surg | USA | A |
| 2017 | 28735427 | Biomechanical evaluation of distal biceps tendon repair using tension slide technique and knotless fixation technique | Int Orthop | USA | B |
| 2017 | 28739023 | Suture bridge transosseous equivalent repair is stronger than transosseous tied braided-tape | J Orthop Sci | Canada | A |
| 2017 | 28749741 | A Biomechanical Analysis of Modern Repair Configurations Versus Traditional Repair Configuration | Am J Sports Med | USA | C |
| 2017 | 28752245 | Which salvage fixation technique is best for the failed initial screw fixation at the cervicothoracic junction? A biomechanical comparison study | Eur Spine J | Korea | A |
| 2017 | 28754246 | A Contact Pressure Analysis Comparing an All-Inside and Inside-Out Surgical Repair Technique for Bucket-Handle Medial Meniscus Tears | Arthroscopy | USA | C |
| 2017 | 28763623 | The Effects of Anterolateral Tenodesis on Tibiofemoral Contact Pressures and Kinematics | Am J Sports Med | UK | A |
| 2017 | 28770357 | Effect of Chestbands on the Global and Local Response of the Human Thorax to Frontal Impact | Ann Biomed Eng | USA | A |
| 2017 | 28770402 | Kinematic efficacy of supplemental anterior lumbar interbody fusion at lumbosacral levels in thoracolumbosacral deformity correction with and without pedicle subtraction osteotomy at L3: an in vitro cadav | Eur Spine J | USA | A |
| 2017 | 28770510 | The posterior bundle of the elbow medial collateral ligament: biomechanical study and proposal for a new reconstruction surgical technique | Musculoskelet Surg | Italy | B |
| 2017 | 28771373 | Biomechanical Evaluation of Glenoid Reconstruction With an Implant-Free J-Bone Graft for Anterior Glenoid Bone Loss | Am J Sports Med | Austria | C |
| 2017 | 28777063 | Comprehensive biomechanical analysis of three reconstruction techniques following total sacrectomy: an in vitro human cadaveric model | J Neurosurg Spine | USA | C |
| 2017 | 28777665 | An Anatomic and Biomechanical Comparison of Bankart Repair Configurations | Am J Sports Med | USA | B |
| 2017 | 28780253 | Biomechanics of human parietal pleura in uniaxial extension | J Mech Behav Biomed Mater | USA | B |
| 2017 | 28795073 | Biomechanical Comparison of a First- and a Second-Generation All-Soft Suture Glenoid Anchor | Orthop J Sports Med | USA | B |
| 2017 | 28801202 | A biomechanical study comparing proximal femur nail and proximal femur locking compression plate in fixation of reverse oblique proximal femur fractures | Injury | India | A |
| 2017 | 28803170 | Anatomy of the alar ligament Part II: Variations of its attachment onto the dens | World Neurosurg | USA | B |
| 2017 | 28807347 | Dorsoradial Instability of the Thumb Metacarpophalangeal Joint: A Biomechanical Investigation | J Hand Surg Am | Korea | C |
| 2017 | 28812035 | Bioabsorbable Versus Metal Screw in the Fixation of Tibial Tubercle Transfer A Cadaveric Biomechanical Study | Orthop J Sports Med | Finland | B |
| 2017 | 28822316 | Improved Middle-Ear Soft-Tissue Visualization Using Synchrotron Radiation Phase-Contrast Imaging | Hear Res | Canada | A |
| 2017 | 28826866 | Anatomy of the alar ligament Part III: Biomechanical study | World Neurosurg | USA | B |
| 2017 | 28831343 | Biomechanical Properties of First Dorsal Extensor Compartment Regarding Adequacy as a BoneLigament-Bone Graft | Plast Reconstr Surg Glob Open | Germany | B |
| 2017 | 28836449 | Adjacent Joint Kinematics After Ankle Arthrodesis During Cadaveric Gait Simulation | Foot Ankle Int | USA | A |
| 2017 | 28844344 | Patellofemoral Contact Pressures After Patellar Distalization: A Biomechanical Study | Arthroscopy | USA | B |
| 2017 | 28852330 | FEMORAL IATROGENIC SUBTROCHANTERIC FATIGUE FRACTURE RISK IS NOT INCREASED BY PLACING DRILL HOLES BELOW THE LEVEL OF THE LESSER TROCHANTER | Iowa Orthop J | USA | C |
| 2017 | 28852840 | Biomechanical study of novel unilateral C1 posterior arch screws and C2 laminar screws combined with an ipsilateral crossed C1–C2 pedicle screw–rod fixation for atlantoaxial instability | Arch Orthop Trauma Surg | China | A |
| 2017 | 28864862 | Is the novel olecranon tension plate a valid alternative to tension band wiring of olecranon fractures? A biomechanical study on cadaver bones | Arch Orthop Trauma Surg | Germany | A |
| 2017 | 28865300 | Technical feasibility of personalized articulating knee joint distraction for treatment of tibiofemoral osteoarthritis | Clin Biomech (Bristol, Avon) | The Netherlands | C |
| 2017 | 28877307 | A Reconfigurable Multiplanar In Vitro Simulator for Real-Time Absolute Motion With External and Musculotendon Forces | J Biomech Eng | USA | C |
| 2017 | 28886667 | The effect of screw trajectory for the reduction and association of the scaphoid and lunate (RASL) procedure: a biomechanical analysis | J Hand Surg Eur Vol | USA | B |
| 2017 | 28888229 | Changes in ankle joint motion after Supramalleolar osteotomy: a cadaveric model | BMC Musculoskelet Disord | Korea | A |
| 2017 | 28888569 | A Biomechanical Analysis of 2 Constructs for Metacarpal Spiral Fracture Fixation in a Cadaver Model: 2 Large Screws Versus 3 Small Screws | J Hand Surg Am | USA | C |
| 2017 | 28894757 | A Biomechanical and Clinical Comparison of Midshaft Clavicle Plate Fixation Are 2 Screws as Good as 3 on Each Side of the Fracture | Orthop J Sports Med | USA | A |
| 2017 | 28898106 | Anterolateral Tenodesis or Anterolateral Ligament Complex Reconstruction: Effect of Flexion Angle at Graft Fixation When Combined With ACL Reconstruction | Am J Sports Med | UK | A |
| 2017 | 28898815 | Biomechanical evaluation of different surgical procedures in single-level transforaminal lumbar interbody fusion in vitro | Clin Biomech (Bristol, Avon) | China | B |
| 2017 | 28899558 | Arthroscopically measured syndesmotic stability after screw vs. suture button fixation in a cadaveric model | Injury | USA | A |

| | | | | | |
|---|---|---|---|---|---|
| 2017 | 28934883 | Contrast-Enhanced Computed Tomography Enables Quantitative Evaluation of Tissue Properties at Intrajoint Regions in Cadaveric Knee Cartilage | Cartilage | Finland | A |
| 2017 | 28935431 | Evaluation of Risk to the Suprascapular Nerve During Arthroscopic SLAP Repair: Is a Posterior Portal Safer? | Arthroscopy | USA | B |
| 2017 | 28936684 | Kinematics of mobile-bearing unicompartmental knee arthroplasty compared to native: results from an in vitro study | Arch Orthop Trauma Surg | Belgium | A |
| 2017 | 28937784 | Lacertus Fibrosus Versus Achilles Allograft Reconstruction for Distal Biceps Tears A Biomechanical Study | Am J Sports Med | USA | B |
| 2017 | 28940110 | Are DXA/aBMD and QCT/FEA Stiffness and Strength Estimates Sensitive to Sex and Age? | Ann Biomed Eng | USA | A |
| 2017 | 28942683 | A Comparison of Plate Versus Screw Fixation for Segmental Scaphoid Fractures: A Biomechanical Study | Hand (N Y) | USA | C |
| 2017 | 28946836 | Biomechanical evaluation of native acromioclavicular joint ligaments and two reconstruction techniques in the presence of the sternoclavicular joint: A cadaver study | J Orthop Surg (Hong Kong) | Lithuania | C |
| 2017 | 28951101 | Arthrokinematics of the Distal Radioulnar Joint Measured Using Intercartilage Distance in an In Vitro Model | J Hand Surg Am | Canada | C |
| 2017 | 28960818 | Biomechanical Stability Analysis of a Stand-alone Cage, Static and Rotational-dynamic Plate in a Twolevel Cervical Fusion Construct | Orthop Surg | USA | C |
| 2017 | 28968479 | Biceps Tenodesis: Biomechanical Assessment of 3 Arthroscopic Suprapectoral Techniques | Orthopedics | USA | C |
| 2017 | 28971694 | Biomechanical Comparison of Intramedullary Fibular Nail Versus Plate and Screw Fixation | Foot Ankle Int | UK | C |
| 2017 | 28985490 | Bone grafting in oblique versus prepared rectangular uncontained glenoid defects in reversed shoulder arthroplasty. A biomechanical compariso | Clin Biomech (Bristol, Avon) | Germany | A |
| 2017 | 28987286 | Determination of sex differences of human cadaveric mandibular condyles using statistical shape and trait modeling | Bone | USA | B |
| 2017 | 28989849 | Can Posterior Lumbar Instrumentation and Fusion Be Overpowered by Anterior Lumbar Fusion With Hyperlordotic Cages? | Global Spine J | USA | B |
| 2017 | 29019902 | In vitro biomechanical comparison after fixed- and mobile-core artificial cervical disc replacement versus fusion | Medicine (Baltimore) | China | A |
| 2017 | 29032860 | Biomechanical comparison of two different locking plates for open wedge high tibial osteotomy | J Orthop Sci | The Netherlands | C |
| 2017 | 29033199 | Biomechanical characteristics of subscapularissparing approach for anatomic total shoulder arthroplasty | J Shoulder Elbow Surg | USA | C |
| 2017 | 29047294 | Deep Transverse Metatarsal Ligament Transection in Morton's Neuroma Excision A Cadaveric Study Examining Effects on Metatarsal Alignment | Foot Ankle Spec | USA | C |
| 2017 | 29048929 | The Effect of Hamstring Tendon Autograft Harvest on the Restoration of Knee Stability in the Setting of Concurrent Anterior Cruciate Ligament and Medial Collateral Ligament Injuries | Am J Sports Med | USA | B |
| 2017 | 29049224 | Primary stability in total hip replacement A biomechanical investigation | Medicine (Baltimore) | Germany | C |
| 2017 | 29053542 | Biomechanical Comparison of Intrapelvic and Extrapelvic Fixation for Acetabular Fractures Involving the Quadrilateral Plate | J Orthop Trauma | USA | C |
| 2017 | 29074465 | Comparison of three-dimensional helical axes of the cervical spine between in vitro and in vivo testing | Spine J | Germany | A |
| 2017 | 29078961 | New parameters describing how knee ligaments carry force in situ predict interspecimen variations in laxity during simulated clinical exams | J Biomech | USA | A |
| 2017 | 29085847 | The Effect of Capsulectomy on Hip Joint Biomechanics | Orthop J Sports Med | USA | B |
| 2017 | 29088590 | Mechanical characterization of digital collateral nerves: a cadaver study | Comput Methods Biomech Biomed Engin | France | C |
| 2017 | 29102266 | Intersegmental Kinetics Significantly Impact Mapping from Finger Musculotendon Forces to Fingertip Forces | J Biomech | USA | C |
| 2017 | 29103848 | The Posterior Bundle's Effect on Posteromedial Elbow Instability After a Transverse Coronoid Fracture: A Biomechanical Study | J Hand Surg Am | USA | C |
| 2017 | 29107220 | Do longus capitis and colli really stabilise the cervical spine? A study of their fascicular anatomy and peak force capabilities | Musculoskelet Sci Pract | New Zealand | A |
| 2017 | 29122424 | Scaphoid Healing Necessary for Unrestricted Activity: A Biomechanical Cadaver Model | J Hand Surg Am | USA | C |
| 2017 | 29128876 | Internal femoral component malrotation in TKA significantly alters tibiofemoral kinematics | Knee Surg Sports Traumatol Arthrosc | Belgium | B |
| 2017 | 29131660 | Biomechanical and Morphometric Properties of Long Flexor Tendons of the Toes: A Cadaver Study | J Am Podiatr Med Assoc | Turkey | A |
| 2017 | 29132789 | Scaphoid Fracture Fixation in a Nonunion Model: A Biomechanical Study Comparing 3 Types of Fixation | J Hand Surg Am | Australia | A |
| 2017 | 29133006 | Anterior Transdiscal Axial Screw Fixation for Subaxial Cervical Spine: A Biomechanical Study | World Neurosurg | China | C |
| 2017 | 29146012 | A biomechanical cadaveric study comparing superior capsule reconstruction using fascia lata allograft with human dermal allograft for irreparable rotator cuff tear | J Shoulder Elbow Surg | USA | C |
| 2017 | 29146508 | Biomechanical Analysis of Palmar Midcarpal Instability and Treatment by Partial Wrist Arthrodesis | J Hand Surg Am | USA | C |
| 2017 | 29146510 | Relative Contributions of the Midcarpal and Radiocarpal Joints to Dart-Thrower's Motion at the Wrist | J Hand Surg Am | USA | C |
| 2017 | 29151008 | Suture Anchor Repair of Complete Proximal Hamstring Ruptures A Cadaveric Biomechanical Evaluation | Bull Hosp Jt Dis (2013) | USA | C |
| 2017 | 29155842 | Posterior column acetabular fracture fixation using a W-shaped angular plate: A biomechanical analysis | PLoS One | China | A |
| 2017 | 29159731 | Effects of Population Variability on Knee Loading During Simulated Human Gait | Ann Biomed Eng | USA | B |
| 2017 | 29162245 | Ultrasound-guided supra-acetabular pin placement in pelvic external fixation: description of a surgical technique and results | Injury | Spain | B |
| 2017 | 29174458 | Anterior cement augmentation of adjacent levels after vertebral body replacement leads to superior stability of the corpectomy cage under cyclic loading-a biomechanical investigation | Spine J | Germany | C |
| 2017 | 29178849 | Goldmann applanation tonometry error relative to true intracameral intraocular pressure in vitro and in vivo | BMC Ophthalmol | USA | A |
| 2017 | 29183087 | Biomechanical Implications of an Oblique Knee Joint Line | J Knee Surg | USA | C |
| 2017 | 29186600 | An in Vitro Biomechanical Model of Differing Pedicle Screw Configurations for Long Construct Segmental Thoracic Fixation | Oper Neurosurg (Hagerstown) | USA | C |
| 2017 | 29188333 | Dynamically tensioned ACL functional knee braces reduce ACL and meniscal strain | Knee Surg Sports Traumatol Arthrosc | Canada | A |
| 2017 | 29205227 | Digital image correlation techniques for strain measurement in a variety of biomechanical test models | Acta Bioeng Biomech | USA | C |
| 2017 | 29214465 | The peripheral soft tissues should not be ignored in the finite element models of the human knee joint | Med Biol Eng Comput | The Netherlands | B |
| 2017 | 29226718 | Contribution to FE modeling for intraoperative pedicle screw strength prediction | Comput Methods Biomech Biomed Engin | France | C |
| 2017 | 29241816 | Triple-bundle anatomical reconstruction using the coracoacromialligament and the short head of biceps tendon to stabilize chronicacromioclavicular joint dislocations: A cadaver feasibility study | Orthop Traumatol Surg Res | France | C |
| 2017 | 29248396 | Biomechanical study comparing Pulvertaft suture to step-cut suture E | Hand Surg Rehabil | France | C |
| 2017 | 29258751 | Intraoperative and Biomechanical Studies of Human Vastus Lateralis and Vastus Medialis Sarcomere Length Operating Range | J Biomech | USA | A |
| 2017 | 29262722 | Syndesmotic Injury Assessment With Lateral Imaging During Stress Testing in a Cadaveric Model | Foot Ankle Int | USA | C |
| 2017 | 29268024 | Anterolateral Knee Extra-articular Stabilizers A Robotic Study Comparing Anterolateral Ligament Reconstruction and Modified Lemaire Lateral Extra-articular Tenodesis | Am J Sports Med | USA | A |
| 2017 | 29269311 | Biomechanical evaluation of C1 lateral mass and C2 translaminar bicortical screws in atlantoaxial fixation: an in vitro human cadaveric study | Spine J | China | C |
| 2017 | 29275548 | Optical spectroscopic characterization of human meniscus biomechanical properties | J Biomed Opt | Finland | A |
| 2017 | 29276534 | The effect of humeral polyethylene insert constraint on reverse shoulder arthroplasty biomechanics | Shoulder Elbow | Canada | B |
| 2017 | 29278079 | Regional and age-dependent residual strains, curvature, and dimensions of the human ureter | Proc Inst Mech Eng H | Greece | A |
| 2017 | 29281797 | Biomechanical Comparison of 3 Glenoid-Side Fixation Techniques for Superior Capsular Reconstruction | Am J Sports Med | USA | B |
| 2017 | 29282138 | The medial femoral wall can play a more important role in unstable intertrochanteric fractures compared with lateral femoral wall: a biomechanical study | J Orthop Surg Res | China | A |
| 2017 | 29287950 | Subpectoral Biceps Tenodesis With PEEK Interference Screw: A Biomechanical Analysis of Humeral Fracture Risk | Arthroscopy | USA | A |
| 2017 | 29309465 | Biomechanical Evaluation of a Novel Suture Augment in Patella Fixation | Am J Orthop (Belle Mead NJ) | USA | C |
| 2017 | 29353123 | A Locking Compression Plate versus the Gold-standard Non-locking Plate with Lag Screw for First Metatarsophalangeal Fusion: a Biomechanical Comparison | Foot (Edinb) | USA | C |
| 2017 | 29353150 | BIODEGRADABLE SPACER REDUCES THE SUBACROMIAL PRESSURE: A BIOMECHANICAL CADAVER STUDY | Clin Biomech (Bristol, Avon) | Germany | A |
| 2017 | 29403249 | "Soft that molds the hard:" Geometric morphometry of lateral atlantoaxial joints focusing on the role of cartilage in changing the contour of bony articular surfaces | J Craniovertebr Junction Spine | India | A |

| | | | | | |
|---|---|---|---|---|---|
| 2017 | 29409214 | Posterolateral plate fixation with Talarlock1 is more stable than screw fixation in ankle arthrodesis in a biomechanical cadaver study | Foot Ankle Surg | Germany | B |
| 2017 | 29409241 | Biomechanical analysis of polyaxial locking vs. non-locking plate fixation of unstable fractures of the distal fibula: A cadaver study with a bone only model | Foot Ankle Surg | Germany | C |
| 2017 | 29409256 | BIOMECHANICAL COMPARISON OF FIXATION STABILITY USING A LISFRANC PLATE VERSUS TRANSARTICULAR SCREWS | Foot Ankle Surg | USA | C |
| 2017 | 29426604 | Damage in a Distal Radius Fracture Model Treated With Locked Volar Plating After Simulated Postoperative Loading | J Hand Surg Am | USA | C |
| 2017 | 29576914 | Biomechanics of an Articulated Screw in Acute Scapholunate Ligament Disruption | J Wrist Surg | USA | B |
| 2017 | 29922496 | Comparison of Locking and Frag-Loc Screws for Fixation of Die-Punch Fragments | J Wrist Surg | Japan | A |
| 2017 | 29983651 | Effects of Abduction Pillows on Rotator Cuff Repair: A Biomechanical Analysis | HSS J | Australia | A |
| 2017 | 30035092 | Biomechanical comparison of pure magnesium interference screw and polylactic acid polymer interference screw in anterior cruciate ligament reconstruction-A cadaveric experimental study | J Orthop Translat | China | A |
| 2017 | 30276113 | Biomechanics of an Expandable Lumbar Interbody Fusion Cage Deployed Through Transforaminal Approach | Int J Spine Surg | USA | C |
| 2017 | 30542230 | Biomechanical Comparison of Fifth Carpometacarpal Fusion Methods: Kirschner Wires Versus Plate and Screws | Tech Orthop | USA | C |
| 2017 | 27029993 | Biomechanical evaluation of CIBOR spine interbody fusion device | J Biomed Mater Res B Appl Biomater | USA | C |
| 2017 | 27570170 | Biomechanical Performance of Medial Row Suture Placement Relative to the Musculotendinous Junction in Transosseous Equivalent Suture Bridge Double-Row Rotator Cuff Repair | Arthroscopy | USA | C |
| 2017 | 27760695 | Effect of lavage and brush preparation on cement penetration and primary stability in tibial unicompartmental total knee arthroplasty: An experimental cadaver study | Knee | Germany | A |
| 2017 | 27838561 | Combined maceration procedure permits advanced microsurgical dissection of Thiel-embalmed specimens | Ann Anat | Switzerland | A |
| 2017 | 27891670 | Biomechanical behavior of novel composite PMMA-CaP bone cements in an anatomically accurate cadaveric vertebroplasty model | J Orthop Res | USA | B |
| 2017 | 28013577 | Plate fixation of periprosthetic femur fractures:What happens to the cement mantle? | Proc Inst Mech Eng H | Germany | B |
| 2017 | 28203592 | Suture-Only Repair Versus Suture Anchor– Augmented Repair for Achilles Tendon Ruptures With a Short Distal Stump | Orthop J Sports Med | USA | B |
| 2017 | 28558915 | Distal skeletal tibia assessed by HR-pQCT is highly correlated with femoral and lumbar vertebra failure loads | J Biomech | Canada | A |
| 2017 | 28567428 | ACL Roof Impingement Revisited Does the Independent Femoral Drilling Technique Avoid Roof Impingement With Anteriorly Placed Tibial Tunn | Orthop J Sports Med | USA | B |
| 2017 | 28752791 | Effect of tibial coronal inclination on hindfoot kinematics: A biomechanical simulation study | Proc Inst Mech Eng H | Japan | A |
| 2017 | 28823940 | Impact of bone quality on the performance of integrated fixation cage screws | Spine J | USA | B |
| 2017 | 28938147 | DEVELOPMENT OF AN EXPERIMENTAL MODEL OF BURST FRACTURE WITH DAMAGE CHARACTERIZATION OF THE VERTEBRAL BODIES UNDER DYNAMIC CONDITIONS | Clin Biomech (Bristol, Avon) | France | C |
| 2017 | 28989848 | Biomechanical Stability of a Stand-Alone Interbody Spacer in Two-Level and Hybrid Cervical Fusion Constructs | Global Spine J | USA | C |
| 2017 | 29157249 | Biomechanical study of isolated radial head dislocation | BMC Musculoskelet Disord | Japan | A |
| 2017 | 29202983 | Influence of the tibialis posterior muscle actuation on the foot anatomy under axial loading; a CT human cadaveric model study | Foot Ankle Surg | Switzerland | C |
| 2017 | 29239252 | Single-Bundle vs Double-Bundle (Anatomical) Reconstruction of the Thumb Ulnar Collateral Ligament: Biomechanical Study | Hand (N Y) | USA | A |
| 2017 | 29279739 | Evaluation of Two Novel Integrated Stand-Alone Spacer Designs Compared with Anterior and Anterior-Posterior Single-Level Lumbar Fusion Techniques: An In Vitro Biomechanical Investigation | Asian Spine J | USA | C |
| 2017 | 29309925 | The quantity of bone cement influences the anchorage of augmented pedicle screws in the osteoporotic spine: A biomechanical human cadaveric study | Clin Biomech (Bristol, Avon) | Germany | A |
| 2017 | 29409299 | Distal fibular malrotation and lateral ankle contact characteristics | Foot Ankle Surg | USA | C |
| 2017 | 29662764 | In vitro biomechanical evaluation of tri-condylar total knee arthroplasty with posterior release for restoration of full extension | J Orthop Translat | Japan | B |
| 2018 | 25037064 | A Cadaveric Biomechanical Analysis of Three Lateral Ulnar Collateral Ligament Reconstructions | J Shoulder Elbow Surg | USA | B |
| 2018 | 28267379 | An injectable, biodegradable calcium phosphatecement containing poly lactic-co-glycolic acidas a bone substitute in ex vivo human vertebralcompression fracture and rabbit bone defectmodels | Connect Tissue Res | China | A |
| 2018 | 28347196 | Distribution of Subchondral Bone Puncture Strength in the Talus and Tibial PlafondA Biomechanical Study | Foot Ankle Spec | USA | C |
| 2018 | 28355680 | Function of the Anterior Intermeniscal Ligament | J Knee Surg | USA | A |
| 2018 | 28431136 | C1 Lateral Mass Displacement and TransverseAtlantal Ligament Failure in Jefferson's Fracture:A Biomechanical Study of the "Rule of Spence" | Neurosurgery | USA | C |
| 2018 | 28448270 | A new in vitro spine test rig to track multiple vertebral motions under physiological conditions | Biomed Tech (Berl) | Germany | B |
| 2018 | 28513885 | Biomechanical function of a balloon nucleus pulposus replacement system: a human cadaveric spine study | J Orthop Res | Korea | C |
| 2018 | 28590060 | Structure-Function of the Disc-Vertebra Interface | J Orthop Res | USA | B |
| 2018 | 28678108 | The Role of Minimally Invasive Vertebral Body Stent on Reduction of the Deflation Effect after Kyphoplasty: a biomechanical study | Spine (Phila Pa 1976) | China | C |
| 2018 | 28759478 | The effects of orientation of lumbar facet joints on the facet joint contact forces: an in vitro biomechanical study | Spine (Phila Pa 1976) | China | C |
| 2018 | 28823938 | The mechanism of thoracolumbar burst fracture may be related to the basivertebral foramen | Spine J | China | A |
| 2018 | 28823940 | Impact of bone quality on the performance of integrated fixation cage screws | Spine J | USA | B |
| 2018 | 28834823 | Biomechanical Analysis Of The Proximal Femoral Locking Compression Plate: Do Quality Of Reduction And Screw Orientation Influence Construct Stability? | J Orthop Trauma | Korea | C |
| 2018 | 28888567 | Biomechanics of the Acute BoutonniereDeformity | J Hand Surg Am | USA | C |
| 2018 | 28922690 | Wound tension and 'closability' with keystone flaps, V-Y flapsand primary closure: a study in fresh-frozen cadavers | ANZ J Surg | Australia | A |
| 2018 | 29268910 | Biomechanical Comparison of Fixation With a Single Screw VersusTwo Kirschner Wires in Distal Chevron Osteotomies of the FirstMetatarsal: A Cadaver Study | J Foot Ankle Surg | Germany | A |
| 2018 | 29290535 | Novel ultrasound assisted suture anchor system using theBoneWelding® technology yields a comparable primary stability inosteopenic and healthy human humeri as a benchmark anchor | Acta Orthop Traumatol Turc | Germany | B |
| 2018 | 29305291 | Medial Patellofemoral Ligament Reconstruction:Impact of Knee Flexion Angle During Graft Fixationon Dynamic Patellofemoral Contact PressuredABiomechanical Study | Arthroscopy | Germany | C |
| 2018 | 29305457 | The role of a small posterior malleolar fragment in trimalleolar fractures | Bone Joint J | Germany | A |
| 2018 | 29309714 | Biomechanical Comparison of Fully Threaded Solid Cortical Versus Partially Threaded Cannulated Cancellous Screw Fixation for Lisfranc Injuries | Orthopedics | USA | C |
| 2018 | 29309925 | The quantity of bone cement influences the anchorage of augmented pediclescrews in the osteoporotic spine: A biomechanical human cadaveric study | Clin Biomech (Bristol, Avon) | Germany | A |
| 2018 | 29319350 | Biomechanical Evaluation of Standard Versus Extended Proximal Fixation Olecranon Plates for Fixation of Olecranon Fractures | Hand (N Y) | USA | B |
| 2018 | 29322875 | Biomechanical Analysis of Capsular Repair Versus Arthrex TFCC Ulnar Tunnel Repair for Triangular Fibrocartilage Complex Tears | Hand (N Y) | USA | B |
| 2018 | 29333900 | Stability of two anteriorfixations for three-columninjury in the lower cervicalspine: biomechanicalevaluation of anterior pediclescrew-plate fixation | Int Med Res | China | A |
| 2018 | 29356940 | The effect of humeral-fenestration diameter in Outerbridge–Kashiwagi arthroplasty on failure load of the distal humerus: a cadaveric biomechanical study | Arch Orthop Trauma Surg | Taiwan | B |
| 2018 | 29370965 | Preventing brachial plexus injury during shouldersurgery: a real-time cadaveric study | J Shoulder Elbow Surg | Australia | C |
| 2018 | 29372167 | A Biomechanical Assessmentof Biceps Femoris Repair Techniques | Orthop J Sports Med | USA | B |
| 2018 | 29373447 | Does Posterior Fixation of Partially Unstable Open-BookPelvic Ring Injuries Decrease Symphyseal Plate Failure? ABiomechanical Study | J Orthop Trauma | USA | C |
| 2018 | 29396585 | Dynamic augmentation restores anterior tibial translation in ACL suture repair: a biomechanical comparison of non-, static and dynamic augmentation techniques | Knee Surg Sports Traumatol Arthrosc | The Netherlands | A |
| 2018 | 29397286 | Arthroscopic Fixation of Tibial Eminence Fractures: ABiomechanical Comparative Study of Screw, Suture,and Suture Anchor | Arthroscopy | China | C |
| 2018 | 29401403 | Patellar Tendon Repair AugmentationWith a Knotless Suture Anchor Internal Brace | Am J Sports Med | USA | B |
| 2018 | 29401410 | Lateral Augmentation Proceduresin Anterior Cruciate LigamentReconstruction | Am J Sports Med | USA | C |
| 2018 | 29407351 | Superior cortical screw in osteoporotic lumbar vertebrae: A biomechanicsand microstructure-based study | Clin Biomech (Bristol, Avon) | China | B |
| 2018 | 29407863 | Biomechanics of a cemented short stem: Standard vs. line-to-linecementation techniques. A biomechanical in-vitro study involving sixosteoporotic pairs of human cadaver femurs | Clin Biomech (Bristol, Avon) | Germany | A |

| | | | | | |
|---|---|---|---|---|---|
| 2018 | 29408275 | A Novel C2 Screw Trajectory: Preliminary Anatomic Feasibility and BiomechanicalComparison | World Neurosurg | USA | C |
| 2018 | 29409390 | Survival Analysis-Based Human Head Injury Risk Curves: Focus on Skull Fracture | J Neurotrauma | USA | A |
| 2018 | 29415564 | Plate Alone Versus Plate and Lag Screw for Lapidus ArthrodesisA Biomechanical Comparison of Compression | Foot Ankle Spec | USA | C |
| 2018 | 29425835 | The influence of internal and external tibial rotation offsets on knee joint and ligament biomechanics during simulated athletic tasks | Clin Biomech (Bristol, Avon) | USA | B |
| 2018 | 29438625 | Biomechanical Evaluation of aSingle- Versus Double-TunnelCoracoclavicular Ligament ReconstructionWith Acromioclavicular Stabilizationfor Acromioclavicular Joint Injuries | Am J Sports Med | USA | B |
| 2018 | 29443845 | Raising the Joint Line in TKA is Associated With Mid-flexionLaxity: A Study in Cadaver Knees | Clin Orthop Relat Res | Belgium | A |
| 2018 | 29445875 | Interference screw insertion angle has no effect on graft fixation strength for insertional Achilles tendon reconstruction | Knee Surg Sports Traumatol Arthrosc | China | A |
| 2018 | 29450567 | Coronal tibial anteromedial tunnel location has minimal effect on knee biomechanics | Knee Surg Sports Traumatol Arthrosc | USA | A |
| 2018 | 29451436 | Novel posterior artificial atlanto-odontoid joint for atlantoaxial instability: a biomechanical study | J Neurosurg Spine | China | A |
| 2018 | 29451935 | Economic Analysis of Anatomic Plating Versus Tubular Plating for the Treatmentof Fibula Fractures | Orthopedics | USA | A |
| 2018 | 29452734 | Calcar screw position in proximal humerus fracture fixation: Don't miss high! | Injury | USA | B |
| 2018 | 29455101 | Does pedicle screw fixation of the subaxial cervical spine provide adequatestabilization in a multilevel vertebral body fracture model? An in vitrobiomechanical study | Clin Biomech (Bristol, Avon) | USA | C |
| 2018 | 29456064 | Triple-Loaded Suture Anchors Versus a Knotless RipStop Construct in a Single-Row Rotator CuffRepair Model | Arthroscopy | USA | B |
| 2018 | 29456066 | Slight Reduction in the Insertion Depth for anAll-Suture Anchor Decreases Cyclic Displacement inthe Shoulder Glenoid | Arthroscopy | USA | C |
| 2018 | 29462730 | Biomechanical stability afforded by unilateral vs. bilateral pedicle screw fixation withand without interbody support using lateral lumbar interbody fusion | World Neurosurg | USA | C |
| 2018 | 29466679 | A Biomechanical Comparisonof Fifth Metatarsal Jones FractureFixation Methods | Am J Sports Med | USA | B |
| 2018 | 29470278 | Pullout strength of pedicle screws following redirection after lateral or medial wall breach | Spine (Phila Pa 1976) | Japan | A |
| 2018 | 29479544 | Ulnar Collateral Ligament ReconstructionVersus Repair With Internal Bracing | Orthop J Sports Med | USA | B |
| 2018 | 29481980 | Biomechanical Evaluation of Sacroiliac Joint Fixation with Decortication | Spine J | USA | C |
| 2018 | 29482611 | Biomechanical evaluation of cervical discreplacement with a novel prosthesis basedon the physiological curvature of endplate | J Orthop Surg Res | China | A |
| 2018 | 29482858 | Biomechanical Evaluation of a TranstendinousAll-Suture Anchor Technique versus InterferenceScrew Technique for Suprapectoral BicepsTenodesis in a Cadaveric Model | Arthroscopy | Taiwan | B |
| 2018 | 29484857 | Biomechanical Analysis of a Novel IntercalaryProsthesis for Humeral Diaphyseal Segmental DefectReconstruction | Orthop Surg | China | C |
| 2018 | 29494715 | Use of a Novel Magnesium-Based Resorbable BoneCement for Augmenting Anchor and Tendon Fixation | Am J Orthop (Belle Mead NJ) | USA | A |
| 2018 | 29497852 | Pedicle screw anchorage of carbon fber-reinforced PEEK screws under cyclic loading | Eur Spine J | Austria | C |
| 2018 | 29500047 | Intracompartmental Versus ExtracompartmentalTransposition of the Extensor Pollicis Longus for TreatingThumb-in-Palm Deformity: A Biomechanical Comparison | J Hand Surg Am | USA | C |
| 2018 | 29505731 | Does Capsular Laxity Leadto Microinstability of the Native Hip? | Am J Sports Med | USA | C |
| 2018 | 29519147 | Biomechanical Cadaveric Evaluation of Partial Acute Peroneal Tendon Tears | Foot Ankle Int | Chile | A |
| 2018 | 29526639 | The effect of inter-body fusion cage design on the stability of the instrumented spine in response to cyclic loading: an experimental study. | Spine J | USA | C |
| 2018 | 29528722 | Biomechanical Evaluation of Circumtibial and Transmembranous Routes for Posterior Tibial Tendon Transfer for Dropfoot | Foot Ankle Int | Chile | C |
| 2018 | 29548956 | Biomechanical Analysis of an Expandable Lumbar Interbody Spacer | World Neurosurg | USA | C |
| 2018 | 29551339 | Suture Tape Augmentation of theThumb Ulnar Collateral Ligament Repair:A Biomechanical Study | J Hand Surg Am | USA | C |
| 2018 | 29554436 | Biomechanical Evaluation of theMedial Stabilizers of the Patella | Am J Sports Med | USA | B |
| 2018 | 29554437 | Does Greater Trochanter DecorticationAffect Suture Anchor Pullout Strengthin Abductor Tendon Repairs? | Am J Sports Med | USA | B |
| 2018 | 29557868 | Articular Contact Area and Pressure in PosteromedialRotatory Instability of the Elbow | J Bone Joint Surg Am | USA | C |
| 2018 | 29558162 | The Integrity of the AcromioclavicularCapsule Ensures PhysiologicalCentering of the AcromioclavicularJoint Under Rotational Loading | Am J Sports Med | Germany | B |
| 2018 | 29559244 | Role of disc area and trabecular bone density on lumbar spinal column fracturerisk curves under vertical impact | J Biomech | USA | A |
| 2018 | 29567517 | Biomechanical comparative study on stability of injectable pedicle screw with 1 different lateral holes augmented with different volumes of polymethylmethacrylate in osteoporotic lumbar vertebrae | Spine J | China | A |
| 2018 | 29568787 | Sex-Based Differences in Knee KineticsWith Anterior Cruciate Ligament Strainon Cadaveric Impact Simulations | Orthop J Sports Med | USA | A |
| 2018 | 29570751 | Applying a Hybrid Experimental  Computational Technique toStudy Elbow Joint LigamentousStabilizers | J Biomech Eng | Canada | C |
| 2018 | 29573895 | The Effect of Dorsally Angulated DistalRadius Deformities on Carpal Kinematics:An In Vitro Biomechanical Study | J Hand Surg Am | Canada | C |
| 2018 | 29579720 | Biomechanical analysis of the durability of a modified S1 vertebraetranspedicular screws insertion technique | Clin Biomech (Bristol, Avon) | Poland | A |
| 2018 | 29580744 | Anthropometric Study of the Radiocapitellar Joint | J Hand Surg Am | USA | A |
| 2018 | 29581069 | Capitellar Erosion after Radial Head Arthroplasty: AComparative Biomechanical Study of Operated Radial HeadFractures on Cadaveric Specimens | Orthop Traumatol Surg Res | Greece | A |
| 2018 | 29600262 | The Stabilising Effect of the Anterior Oblique Ligament to Prevent Directional Subluxation at the Trapeziometacarpal Joint of the Thumb: A Biomechanical Cadaveric Study | Arch Bone Jt Surg | UK | C |
| 2018 | 29600720 | Effect of Ankle Position on Tibiotalar Motion With Screw Fixation of the Distal Tibiofibular Syndesmosis in a Fracture Model | Foot Ankle Int | USA | C |
| 2018 | 29611163 | Cadaveric-biomechanical study on medialretinaculum: its stabilising role for the patella against lateral dislocation | Folia Morphol (Warsz) | Greece | A |
| 2018 | 29616587 | Biomechanical Strength of Retrograde Fixation in Proximal Third Scaphoid Fractures | Hand (N Y) | USA | A |
| 2018 | 29627133 | Quantitative evaluation of facet deflection, stiffness, strain and failure load during simulated cervical spine trauma | J Biomech | Australia | C |
| 2018 | 29627641 | Occiput-axis crossing translaminar screw fixation technique using offsetconnectors: An in vitro biomechanical study | Clin Neurol Neurosurg | China | A |
| 2018 | 29627930 | Flexor digitorum longus tendon transfer to the navicular: tendon-totendon repair is stronger compared with interference screw fixation | Knee Surg Sports Traumatol Arthrosc | UK | A |
| 2018 | 29628315 | A bigger suture diameter for anterior cruciate ligament all-inside graft linkpreparation leads to better graft stability: An anatomical specimen study | Knee | Austria | C |
| 2018 | 29635141 | Posterior cervical spine crisscross fixation: Biomechanical evaluation | Clin Biomech (Bristol, Avon) | USA | C |
| 2018 | 29635561 | Biomechanical Measurement and Modeling of Human EardrumInjury in Relation to Blast Wave Direction | Mil Med | USA | A |
| 2018 | 29637082 | Quantitative Anatomic Analysisof the Medial Ulnar CollateralLigament Complex of the Elbow | Orthop J Sports Med | USA | A |
| 2018 | 29649670 | The influence of the twin peg design on femoral Interface temperature andmaximum load to failure in cemented Oxford unicompartmental kneearthroplasty | Clin Biomech (Bristol, Avon) | Germany | A |
| 2018 | 29652192 | Achilles Pullout Strength After Open Calcaneoplasty for Haglund Syndrome | Foot Ankle Int | USA | C |
| 2018 | 29655486 | Comprehensive simulation on morphological and mechanical properties of trig  ger finger – A cadaveric model | J Biomech | Taiwan | A |
| 2018 | 29657449 | Analysis of glenoid inter-anchor distance with an all-suture anchor system | J Orthop | USA | C |
| 2018 | 29662914 | Ligament Strain Response BetweenLower Extremity Contralateral PairsDuring In Vitro Landing Simulation | Orthop J Sports Med | USA | B |
| 2018 | 29664884 | Biomechanical Testing of a 3-Hole versus a 4-Hole Sliding Hip Screw in the presence of a Retrograde Intramedullary Nail for Ipsilateral Intertrochanteric and Femur Shaft Fractures | J Orthop Trauma | Canada | C |
| 2018 | 29671348 | Four-Strand Versus 2-Strand Suture-Button Constructs in First Carpometacarpal Arthroplasty: A Biomechanical Study | Hand (N Y) | USA | A |
| 2018 | 29678191 | A biomechanical analysis of triangularmedial knee reconstruction | BMC Musculoskelet Disord | China | A |
| 2018 | 29678397 | Varus posteromedial rotatory instability: abiomechanical analysis of posterior bundle of themedial ulnar collateral ligament reconstruction | J Shoulder Elbow Surg | USA | C |
| 2018 | 29678714 | Transmuscle ultrasonography in the placement of thoracolumbar pedicle screws: Acadaveric study | World Neurosurg | China | A |

| | | | | | |
|---|---|---|---|---|---|
| 2018 | 29682310 | Rehabilitation following meniscal repair: a systematic review | BMJ Open Sport Exerc Med | USA | C |
| 2018 | 29684924 | Fixation Strength of Pedicle and Cortical LumbarVertebral Screws after Laminectomy:A Cadaver Study | J Neurol Surg A Cent Eur Neurosurg | Korea | C |
| 2018 | 29685388 | The contribution of the posterolateral capsuleto elbow joint stability: a cadavericbiomechanical investigation | J Shoulder Elbow Surg | UK | B |
| 2018 | 29708573 | Medial Compartment Decompression by Proximal Fibular Osteotomy: A Biomechanical Cadaver Study | Orthopedics | USA | B |
| 2018 | 29716591 | The primary stability of diferent implants for intra-articular calcaneal fractures: an in vitro study | Biomed Eng Online | China | A |
| 2018 | 29740675 | Biomechanical advantages of supplemental accessory and satellite rods with and without interbody cages implantation for the stabilization of pedicle subtraction osteotomy | Eur Spine J | Italy | B |
| 2018 | 29741391 | Relationship Between Deltoid andRotator Cuff Muscles DuringDynamic Shoulder Abduction | Am J Sports Med | USA | A |
| 2018 | 29741957 | Effects of Proud Large Osteochondral Plugs on Contact Forces and Knee Kinematics: A Robotic Study | Am J Sports Med | USA | B |
| 2018 | 29746150 | Biomechanical Comparison of Anatomicand Extra-Anatomic ReconstructionTechniques Using Local Grafts for ChronicInstability of the Acromioclavicular Joint | Am J Sports Med | France | A |
| 2018 | 29748121 | The biomechanical evaluation of a novel 3-stranddocking technique for ulnar collateral ligamentreconstruction in the elbow | J Shoulder Elbow Surg | USA | A |
| 2018 | 29752053 | Anterior Laxity, Lateral Tibial Slope, and In Situ ACL Force Differentiate KneesExhibiting Distinct Patterns of Motion during a Pivoting Event: A Human Ca daveric Study | J Biomech | USA | A |
| 2018 | 29753560 | Application of resampling techniques to improve the quality of survival analysis risk curves for human frontal bone fracture | Clin Biomech (Bristol, Avon) | USA | C |
| 2018 | 29763339 | The V-Shaped Distal Triceps Tendon RepairA Comparative Biomechanical Analysis | Am J Sports Med | Germany | C |
| 2018 | 29766741 | Simultaneous Intraoperative Measurement of Cadaver Ankle and Subtalar Joint Compression During Arthrodesis With Intramedullary Nail, Screws, and Tibiotalocalcaneal Plate | Foot Ankle Int | USA | C |
| 2018 | 29767346 | Biomechanical properties of suprapectoral biceps tenodesis: double knotless screw fixation is superior to single knotless screw fixation | Arch Orthop Trauma Surg | Taiwan | B |
| 2018 | 29774947 | Passive engineering mechanism enhancement of a flexor digitorum longus tendon transfer procedure | J Orthop Res | USA | B |
| 2018 | 29780840 | Radial Tears of the Lateral Meniscus—Two Novel Repair Techniques | Orthop J Sports Med | USA | C |
| 2018 | 29783086 | Biomechanical comparison of cemented vs. non-cemented anterior screw fixation in type II odontoid fractures in the elderly: A cadaveric study | Spine J | UK | A |
| 2018 | 29784247 | Evaluation of the head-helmet sliding properties in an impact test | J Biomech | Ireland | A |
| 2018 | 29787981 | Autologous bridging of rotator cuff tears with a hamstring tendon patch. Acadaver feasibility study and biomechanical testing | Clin Biomech (Bristol, Avon) | Germany | A |
| 2018 | 29789250 | Sectioning of the Anterior Intermeniscal LigamentChanges Knee Loading Mechanics | Arthroscopy | France | B |
| 2018 | 29797166 | Strength of suture-button fixation versus ligament reconstructionin syndesmotic injury: a biomechanical study | Int Orthop | China | A |
| 2018 | 29801934 | Washer and Post Augmentation of 90/90Wiring for Proximal Interphalangeal JointArthrodesis: A Biomechanical Study | J Hand Surg Am | USA | C |
| 2018 | 29846753 | Increased valgus laxity in flexion with greater tibial resection depth following total knee arthroplasty | Knee Surg Sports Traumatol Arthrosc | France | A |
| 2018 | 29847143 | Interference Screw Versus Suture Endobutton Fixation of a Fiber-Reinforced Meniscus Replacement Device in a Human Cadaveric Knee Model | Am J Sports Med | USA | B |
| 2018 | 29847148 | Effect of Meniscocapsular and Meniscotibial Lesions in ACL-Deficient and ACL-Reconstructed Knees: A Biomechanical Study | Am J Sports Med | USA | B |
| 2018 | 29851867 | What Are the Effects of Capsular Plication on Translational Laxityof the Glenohumeral Joint: A Study in Cadaveric Shoulders | Clin Orthop Relat Res | USA | A |
| 2018 | 29852313 | The Rib Cage Stiffens the Thoracic Spine in aCadaveric Model with Body Weight Load underDynamic Moments | J Mech Behav Biomed Mater | USA | C |
| 2018 | 29852314 | Time-elapsed synchrotron-light microstructural imaging of femoral neck fracture | J Mech Behav Biomed Mater | Australia | A |
| 2018 | 29858655 | Implant preloading in extension reduces spring length change in dynamic intraligamentary stabilization: a biomechanical study on passive kinematics of the knee | Knee Surg Sports Traumatol Arthrosc | Switzerland | A |
| 2018 | 29864374 | Validation of Noncontact Anterior Cruciate Ligament Tears Produced by a Mechanical Impact Simulator Against the Clinical Presentation of Injury | Am J Sports Med | USA | B |
| 2018 | 29879483 | The mechanical properties of fresh versus fresh-frozenand preserved (Thiel and formalin) long head of bicepstendons: a cadaveric investigation | Ann Anat | UAE | B |
| 2018 | 29881231 | Biomechanical comparison of a novel C1 posterior U-construct with fourother techniques in a C1–C2 fixation model | J Orthop | USA | A |
| 2018 | 29891266 | Bennett Fractures: A Biomechanical Modeland Relevant Ligamentous Anatomy | J Hand Surg Am | USA | A |
| 2018 | 29914671 | Cement-augmented sacroiliac screw fixation withcannulated versus perforated screws – a biomechanical studyin an osteoporotic hemipelvis model | Injury | Germany | A |
| 2018 | 29916933 | A Locked Intraosseous Nail for TransversePatellar Fractures | J Bone Joint Surg Am | Germany | A |
| 2018 | 29932749 | A Cadaveric Study of Metatarsal Length and Its Function in the Metatarsal Formula and Forefoot Pathology | J Am Podiatr Med Assoc | USA | B |
| 2018 | 29933084 | Assessment of Biomechanical Changes After Sacroiliac Joint Fusion by Applicationof the 3-Dimensional Motion Analysis Technique | World Neurosurg | USA | C |
| 2018 | 29947839 | Bicruciate lesion biomechanics, Part 1—Diagnosis: translations over 15 mm at 90° of knee flexion are indicative of a complete tear | Knee Surg Sports Traumatol Arthrosc | Brazil | A |
| 2018 | 29950134 | A comparison of dart thrower's rangeof motion following radioscapholunatefusion, four-corner fusion andproximal row carpectomy | J Hand Surg Eur Vol | UK | C |
| 2018 | 29953291 | The Influence of Graft TensioningSequence on Tibiofemoral OrientationDuring Bicruciate and PosterolateralCorner Knee Ligament Reconstruction | Am J Sports Med | USA | C |
| 2018 | 29956015 | Impact of five different medialpatellofemoral ligament-reconstructionstrategies and three different graft pre tensioning states on the meanpatellofemoral contact pressure: abiomechanical study on human cad | J Exp Orthop | Germany | A |
| 2018 | 29960627 | Biomechanical Comparison of Inflatable Penile Implants: A CadavericPilot Study | J Sex Med | USA | C |
| 2018 | 29977937 | Biomechanical Comparison of EpiphysealAnterior Cruciate Ligament Fixation Usinga Cortical Button Construct Versusan Interference Screw and Sheath Constructin Skeletally Immature Cadaveric Specime | Orthop J Sports Med | USA | B |
| 2018 | 29977940 | Biomechanical Comparison of Screw FixationVersus a Cortical Button and Self-tensioningSuture for the Latarjet Procedure | Orthop J Sports Med | USA | B |
| 2018 | 29984195 | Comparison of a simplified skin pointer device compared with a skeletal marker for knee rotation laxity: A cadaveric study using a rotation-meter | World J Orthop | Singapore | C |
| 2018 | 29987700 | Comparison of HR-pQCT- and microCT-based finite element modelsfor the estimation of the mechanical properties of the calcaneustrabecular bone | Biomech Model Mechanobiol | UK | A |
| 2018 | 30005173 | Biomechanical Comparison of Syndesmotic Repair Techniques During External Rotation Stress | Foot Ankle Int | USA | C |
| 2018 | 30007821 | Effect of radiocapitellar Achilles disc arthroplasty on coronoid and capitellar contact pressures after radial head excision | J Shoulder Elbow Surg | USA | C |
| 2018 | 30011055 | A Biomechanical Study of an Alternative Internal Fixation Method for Transverse Patella Fractures | Orthopedics | China | A |
| 2018 | 30029268 | The Size of Simulated Lytic Metastases Affects the Strain Distribution on the Anterior Surface of the Vertebra | J Biomech Eng | Italy | A |
| 2018 | 30034430 | The biomechanical study of rupture of Achilles Tendon and repair by different suture techniques | Pak J Med Sci | China | B |
| 2018 | 30035119 | Nonlinear Biomechanical Characteristics of the SchneiderianMembrane: Experimental Study and Numerical Modeling | Biomed Res Int | China | A |
| 2018 | 30038920 | Superior Capsular Reconstruction Witha Long Head of the Biceps Tendon AutograftA Cadaveric Study | Orthop J Sports Med | USA | B |
| 2018 | 30046634 | A Biomechanical and Structural Comparisonof Articular Cartilage and Subchondral Boneof the Glenoid and Humeral Head | Orthop J Sports Med | USA | C |
| 2018 | 30060367 | In Vitro Biomechanical and Fluoroscopic Study of a Continuously Expandable Interbody Spacer Concerning Its Role in Insertion Force and Segmental Kinematics | Asian Spine J | USA | C |
| 2018 | 30062459 | Retaining or excising the supraspinatus tendon in complex proximal humeral fractures treated with reverse prosthesis: a biomechanical analysis in two different designs | Arch Orthop Trauma Surg | Spain | C |
| 2018 | 30062643 | Isolated medial patellofemoral ligament reconstruction for patella instability is insufficient for higher degrees of internal femoral torsion | Knee Surg Sports Traumatol Arthrosc | Austria | B |
| 2018 | 30062936 | The role of the posterior bundle of the medial collateral ligament in posteromedial rotatory instability of the elbow | Bone Joint J | USA | A |
| 2018 | 30067131 | Early shape change behaviour of an uncemented contemporary hip cup: A cadaveric experiment replicating host bone behaviour through temperature control | Proc Inst Mech Eng H | UK | A |
| 2018 | 30069505 | Biomechanical evaluation of interbody fixation with secondary augmentation: lateral lumbar interbody fusion versus posterior lumbar interbody fusion | J Spine Surg | USA | A |
| 2018 | 30070599 | Comparison of Anatomic Structures at Risk With 2 Lateral Lengthening Calcaneal Osteotomies | Foot Ankle Int | Germany | B |
| 2018 | 30075035 | Biomechanical Analysis of a Novel Buried FixationTechnique Using Headless Compression Screws for theTreatment of Patella Fractures | Am J Orthop (Belle Mead NJ) | USA | B |
| 2018 | 30078687 | Anterior Cable Reconstruction Using the ProximalBiceps Tendon for Large Rotator Cuff Defects LimitsSuperior Migration and Subacromial ContactWithout Inhibiting Range of Motion:A Biomechanical An | Arthroscopy | USA | C |

| | | | | | |
|---|---|---|---|---|---|
| 2018 | 30086040 | Biomechanical Assessment of Locking Plate Fixation ofComminuted Proximal Olecranon Fractures | J Orthop Trauma | USA | B |
| 2018 | 30090679 | A MECHANISM FOR LIGAMENTUM TERES INJURIES IN FEMOROACETABULAR IMPINGEMENT: AN ANATOMICAL STUDY | Int J Sports Phys Ther | USA | A |
| 2018 | 30090835 | Comparison of a Distal Tibial Allograftand Scapular Spinal Autograft for PosteriorShoulder Instability With Glenoid Bone Loss | Orthop J Sports Med | USA | B |
| 2018 | 30092463 | Biomechanical Fixation Properties of the Cortical Bone Trajectory in the OsteoporoticLumbar Spine | World Neurosurg | China | A |
| 2018 | 30093233 | Distal triceps transosseous cruciate versus sutureanchor repair using equal constructs: abiomechanical comparison | J Shoulder Elbow Surg | USA | B |
| 2018 | 30096469 | Factors associated with proximal femur fracture determined in a large cadaveric cohort | Bone | USA | A |
| 2018 | 30109240 | Hyperflexion and Femoral InterferenceScrew Insertion in ACL Reconstruction | Orthop J Sports Med | USA | B |
| 2018 | 30114533 | A Novel Competing Risk Analysis Model to Determine the Role of Cervical Lordosis in Bony and Ligamentous Injuries | World Neurosurg | USA | C |
| 2018 | 30116763 | Influence of the Anterolateral Ligamenton Knee LaxityA Biomechanical Cadaveric Study Measuring KneeKinematics in 6 Degrees of Freedom Using DynamicRadiostereometric Analysis | Orthop J Sports Med | Denmark | A |
| 2018 | 30117192 | Improved biomechanics of two alternative kyphoplasty cementation methods limit vertebral recollapse | J Orthop Res | USA | C |
| 2018 | 30123736 | Comparison of anterior cruciate ligamentreconstruction methods between reverse“Y” plasty reconstruction and traditionalsingle-bundle techniquedA cadaveric study | J Orthop Translat | China | A |
| 2018 | 30128979 | Can tape–screw fxation of a quadrupled semitendinosus graft in a full-length tibial tunnel provide superior fxation compared with a doubled semitendinosus–gracilis held with an interference screw? A match | J Orthop Traumatol | Australia | A |
| 2018 | 30131112 | Mapping anisotropy improves QCT-based finite element estimation ofhip strength in pooled stance and side-fall load configurations | Med Eng Phys | Switzerland | A |
| 2018 | 30141241 | Preliminary biomechanical results of a novel pin configurationfor external fixation of vertical shear pelvic fractures | ANZ J Surg | New Zealand | A |
| 2018 | 30146132 | The impact of surgical alar (lower lateral) cartilage manipulation onlateral crural stiffness | J Plast Reconstr Aesthet Surg | Canada | A |
| 2018 | 30150641 | Quadruple Semitendinosus Graft Construct With Double Cortical Suspensory Fixation for Anterior Cruciate Ligament Reconstruction: A Biomechanical Study | Sci Rep | USA | A |
| 2018 | 30168784 | Biomechanical evaluation of the ProDisc-C stability following graded posterior cervical injury | J Neurosurg Spine | Canada | C |
| 2018 | 30168835 | Proximal Humeral Locking Plates: A Cadaveric Study of 5 Versus 7 Metaphyseal Locking Screws | Orthopedics | USA | C |
| 2018 | 30172764 | Integrity of a Single Superior Border Plate Repair in Mandibular Angle Fracture: ANovel Cadaveric Human Mandible Model | J Oral Maxillofac Surg | UK | B |
| 2018 | 30180063 | ACL Function in Bicruciate-RetainingTotal Knee Arthroplasty | J Bone Joint Surg Am | Japan | A |
| 2018 | 30188343 | Tibiofemoral Contact Mechanics With Horizontal Cleavage Tearsand Treatment of the Lateral Meniscus in the Human Knee: AnIn Vitro Cadaver Study | Clin Orthop Relat Res | USA | A |
| 2018 | 30189438 | Biomechanical Evaluation of Suture Anchor versusTransosseous Tunnel Patellar Tendon RepairTechniques | J Knee Surg | USA | C |
| 2018 | 30192220 | Biomechanical study of injectable hollow pedicle screws for PMMA augmentation in severely osteoporotic lumbar vertebrae: effect of PMMA distribution and volume on screw stability | J Neurosurg Spine | China | A |
| 2018 | 30195950 | Greater Tuberosity Decortication Decreases Load toFailure of All-Suture Anchor Constructs in RotatorCuff Repair | Arthroscopy | USA | B |
| 2018 | 30195952 | Comparison of Curved and Straight Anchor Insertionfor SLAP Repair: A Cadaveric Study | Arthroscopy | USA | B |
| 2018 | 30197151 | Quantified in vitro tibiofemoral contact during bodyweight back squats | J Biomech | USA | C |
| 2018 | 30200814 | The effect of unloader knee braces on medial meniscal strain | Prosthet Orthot Int | Canada | A |
| 2018 | 30201269 | Biomechanical Evaluation of Lumbar Lateral Interbody Fusion for theTreatment of Adjacent Segment Disease | Spine J | USA | C |
| 2018 | 30202715 | Biomechanics of L5/S1 in LongThoracolumbosacral Constructs:A Cadaveric Study | Global Spine J | USA | B |
| 2018 | 30210257 | SACRECTOMY ASSOCIATED WITH VERTEBRECTOMY: A NEW TECHNIQUE USING DOWEL GRAFTS FROM CADAVERS | Acta Ortop Bras | Brazil | A |
| 2018 | 30213679 | Attenuation of Proximal Junctional Kyphosis Using Sublaminar Polyester Tension Bands:A Biomechanical Study | World Neurosurg | USA | B |
| 2018 | 30217556 | Automated, accurate, and three-dimensional method for calculating sagittal slope of the tibial plateau | J Biomech | USA | C |
| 2018 | 30221084 | A novel experimental design for the measurement of metacarpal bone loading and deformation and fingertip force | PeerJ | Belgium | A |
| 2018 | 30222791 | Biomechanical Properties of the Lateral Patellofemoral Ligament: A Cadaveric Analysis | Orthopedics | USA | C |
| 2018 | 30225930 | The digital human forearm and hand | J Anat | Belgium | B |
| 2018 | 30229701 | Experimental assessment of biomechanical properties in human male elbow bone subjected to bending and compression loads | J Appl Biomater Funct Mater | India | A |
| 2018 | 30241733 | A Biomechanical Cadaver Comparison of Suture ButtonFixation to Plate Fixation for Pubic Symphysis Diastasis | Injury | USA | C |
| 2018 | 30247278 | Biomechanical Evaluation of Osteoporotic ProximalPeriprosthetic Femur Fractures With Proximal BicorticalFixation and Allograft Struts | J Orthop Trauma | USA | C |
| 2018 | 30265223 | The distribution of brachial plexus lesions after experimental traction: a cadaveric study | J Neurosurg Spine | Poland | C |
| 2018 | 30265639 | “Hockey wrist”: dorsal ulnotriquetral ligament injury | Can J Surg | Canada | A |
| 2018 | 30265819 | The Effect of Lateral Opening Wedge DistalFemoral Varus Osteotomy on TibiofemoralContact Mechanics Through Knee Flexion | Am J Sports Med | USA | B |
| 2018 | 30265820 | Effect of Increased Scapular InternalRotation on Glenohumeral ExternalRotation and Elbow Valgus Load in theLate Cocking Phase of Throwing Motion | Am J Sports Med | Japan | A |
| 2018 | 30267188 | Bicruciate lesion biomechanics, Part 2-treatment using a simultaneous tensioning protocol: ACL fixation first is better than PCL fixation first to restore tibiofemoral orientation | Knee Surg Sports Traumatol Arthrosc | Brazil | A |
| 2018 | 30267994 | Novel adhesives for distal radius fixation: A biomechanical analysis | J Mech Behav Biomed Mater | Canada | B |
| 2018 | 30269168 | Release of the medial collateral ligament is mandatory in medial open-wedge high tibial osteotomy | Knee Surg Sports Traumatol Arthrosc | Germany | A |
| 2018 | 30269184 | Radiological and biomechanical assessment of displaced greatertuberosity fractures | Int Orthop | Switzerland | A |
| 2018 | 30274689 | Periprosthetic bone quality affects the fixation of anatomic glenoids in total shoulder arthroplasty: in vitro study | J Shoulder Elbow Surg | Germany | A |
| 2018 | 30280088 | Anterior Fixation of Floating Facet Fractures in the CervicalSpine: A Prospective Case Series and BiomechanicalAnalysis | Int J Spine Surg | USA | C |
| 2018 | 30282096 | Onlay Reconstruction of the Posterior CruciateLigament: Biomechanical Comparison ofUnicortical and Bicortical Tibial Fixation | J Knee Surg | Brazil | C |
| 2018 | 30282101 | Effect of Femoral Antetorsion on Tibiofemoral Translation and Rotation in the Anterior Cruciate Ligament Deficient Knee | J Knee Surg | Germany | A |
| 2018 | 30285460 | Double-Layer Rotator Cuff RepairAnatomic Reconstruction of the Superior Capsuleand Rotator Cuff Improves Biomechanical Propertiesin Repairs of Delaminated Rotator Cuff Tears | Am J Sports Med | Austria | C |
| 2018 | 30286774 | Tibiofemoral joint contact area and stress after single-bundle anterior cruciate ligament reconstruction with transtibial versus anteromedial portal drilling techniques | J Orthop Surg Res | China | A |
| 2018 | 30291397 | High tibial osteotomy increases patellofemoral pressure if adverted proximal, while open-wedge HTO with distal biplanar osteotomy discharges the patellofemoral joint: different open-wedge high tibial osteo | Knee Surg Sports Traumatol Arthrosc | Germany | A |
| 2018 | 30292063 | Contact force between the tibial spine and medial femoralcondyle: A biomechanical study | Clin Biomech (Bristol, Avon) | USA | B |
| 2018 | 30292596 | Biomechanical Analysis of Single-, Double-, andTriple-Bundle Configurations for CoracoclavicularLigament Reconstruction Using Cortical FixationButtons With Suture Tapes: A Cadaveric Study | Arthroscopy | Korea | C |
| 2018 | 30292597 | Remplissage Using Interconnected Knotless Anchors:Superior Biomechanical Properties to a KnottedTechnique? | Arthroscopy | USA | A |
| 2018 | 30305126 | Effect of pedicle screw augmentation witha self-curing elastomeric material undercranio-caudal cyclic loading—a cadavericbiomechanical study | J Orthop Surg Res | Austria | B |
| 2018 | 30306219 | Screw tip augmentation leads to improved primary stabilityin the minimally invasive treatment of displaced intra-articular fracturesof the calcaneus: a biomechanical study | Int Orthop | Austria | C |
| 2018 | 30306240 | Repair of the entire superior acromioclavicular ligament complex best restores posterior translation and rotational stability | Knee Surg Sports Traumatol Arthrosc | USA | B |
| 2018 | 30306891 | The geometrical axis of the talocrural joint-Suggestions for a new measurement of the talocrural joint axis | Foot Ankle Surg | Germany | C |
| 2018 | 30306892 | Ankle morphometry based on computerized tomography | Foot Ankle Surg | Germany | C |
| 2018 | 30307738 | Vertical Extension of the T-CapsulotomyIncision in Hip Arthroscopic SurgeryDoes Not Affect the Force Requiredfor Hip Distraction | Am J Sports Med | USA | C |
| 2018 | 30309796 | The impact of harvest length and detachment of the interosseousmembrane on donor-site morbidity following free fibula flapsurgeryda biomechanical experimental study | J Craniomaxillofac Surg | Germany | A |
| 2018 | 30314696 | All arthroscopic remnant-preserving reconstruction of the lateralligaments of the ankle: A biomechanical study and clinical application | Biochem Biophys Res Commun | China | A |

| | | | | | |
|---|---|---|---|---|---|
| 2018 | 30316165 | Biomechanical effects of position and angle of insertion for all-sutureanchors in arthroscopic Bankart repair | Clin Biomech (Bristol, Avon) | Korea | C |
| 2018 | 30321928 | Biomechanical comparison of conventional versus modified techniquein distal chevron osteotomies of the first metatarsal: A cadaver study | Foot Ankle Surg | Germany | A |
| 2018 | 30321946 | Screw fixation of the syndesmosis alters joint contact characteristics in an axially loaded cadaveric model | Foot Ankle Surg | USA | B |
| 2018 | 30326686 | Role of Posterior Ligamentous Reinforcement in Proximal Junctional Kyphosis: A Cadaveric Biomechanical Study | Asian Spine J | USA | B |
| 2018 | 30334549 | Ex Vivo Evaluation of Hip Fracture Risk by Proximal Femur Geometry and Bone Mineral Density in Elderly Chinese Women | Med Sci Monit | China | A |
| 2018 | 30335764 | In vitro biomechanical evaluation of amonocoque plate-spacer construct forcervical open-door laminoplasty | PLoS One | Japan | A |
| 2018 | 30337267 | Effects of axial forearm instability on force transmission across the elbow | J Shoulder Elbow Surg | USA | C |
| 2018 | 30342380 | Suture-button fixation and anterior inferior tibiofibular ligamentaugmentation with suture-tape for syndesmosis injury: A biomechanicalcadaveric study | Clin Biomech (Bristol, Avon) | Japan | A |
| 2018 | 30345830 | Proximal Interphalangeal Arthrodesis of Lesser Toes Utilizing K-Wires Versus Expanding Implants: Comparative Biomechanical Cadaveric Study | Foot Ankle Int | USA | C |
| 2018 | 30349753 | Biomechanical Strength of Scaphoid Partial Unions | J Wrist Surg | USA | B |
| 2018 | 30364882 | In Vitro Biomechanical Evaluation of a Novel, MinimallyInvasive, Sacroiliac Joint Fixation Device | Int J Spine Surg | USA | C |
| 2018 | 30370439 | Kinematically aligned total knee arthroplasty reproduces native patellofemoral biomechanics during deep knee flexion | Knee Surg Sports Traumatol Arthrosc | Korea | B |
| 2018 | 30372476 | Biomechanical evaluation of hybrid doubleplate osteosynthesis using a locking plate andan inverted third tubular plate for thetreatment of proximal humeral fractures | PLoS One | Germany | A |
| 2018 | 30374032 | Pelvic orthosis efects on posterior pelvis kinematics An in-vitrobiomechanical study | Sci Rep | Germany | B |
| 2018 | 30377622 | Biomechanical Comparison of3 Syndesmosis Repair TechniquesWith Suture Button Implants | Orthop J Sports Med | USA | C |
| 2018 | 30382750 | Effects of Capsular ReconstructionWith an Iliotibial Band Allograft on Distractive Stability of the Hip Joint | Am J Sports Med | USA | C |
| 2018 | 30391767 | Total Disk Replacement Adjacent to a Multilevel Fusion in the Cervical Spine: ABiomechanical Motion Analysis | World Neurosurg | USA | C |
| 2018 | 30403369 | Biomechanical Comparison of UlnarCollateral Ligament ReconstructionWith the Docking TechniqueVersus Repair With Internal Bracing | Am J Sports Med | USA | C |
| 2018 | 30414794 | The biomechanical and morphological characteristics of the ligamentummucosum and its potential role in anterior knee pain | Knee | Canada | A |
| 2018 | 30419178 | Primary Stability of an AcromioclavicularJoint Repair Is Affected by the Typeof Additional Reconstruction of theAcromioclavicular Capsule | Am J Sports Med | Germany | B |
| 2018 | 30420164 | Robotic hip joint testing: Development and experimental protocols | Med Eng Phys | UK | A |
| 2018 | 30420195 | The Relationship Between Hemihamate GraftSize and Proximal Interphalangeal Joint Flexionfor Reconstruction of Fracture-Dislocations:A Biomechanical Study | J Hand Surg Am | USA | C |
| 2018 | 30447984 | A Biomechanical Study Comparing Minimally InvasiveAnterior Pelvic Ring Fixation Techniques to External Fixation | Injury | USA | B |
| 2018 | 30456909 | Medially-stabilized total knee arthroplasty does not alter knee laxity and balance in cadaveric knees | J Orthop Res | USA | C |
| 2018 | 30462596 | Biomechanical and clinical comparison of single lateral plate and double plating of comminuted supracondylar femoral fractures | Acta Orthop Belg | China | A |
| 2018 | 30465963 | S1 Pedicle Subtraction Osteotomy in Sagittal Balance Correction. A Feasibility Study onHuman Cadaveric Specimens | World Neurosurg | Spain | B |
| 2018 | 30473370 | Is Bone-Cement Augmentation of Screw-AnchorFixation Systems Superior in Unstable Femoral NeckFractures? A Biomechanical Cadaveric Study | Injury | Germany | A |
| 2018 | 30481240 | The PASTA Bridge – A Repair Technique for PartialArticular-Sided Rotator Cuff Tears: A BiomechanicalEvaluation of Construct Strength | Am J Orthop (Belle Mead NJ) | USA | C |
| 2018 | 30484851 | Use of an Additional Nonlocking Screw in Olecranon Fracture Osteosynthesis Changes Failure Mechanism | Orthopedics | USA | C |
| 2018 | 30495972 | Mechanical Properties and Microstructural Collagen Alignment of the Ulnar Collateral Ligament During Dynamic Loading | Am J Sports Med | USA | B |
| 2018 | 30497219 | Biomechanical assessment of proximal junctional semi-rigid fixation in long-segment thoracolumbar constructs | J Neurosurg Spine | USA | C |
| 2018 | 30509244 | Reattachment of the flexor and extensortendons at the epicondyle in elbowinstability: a biomechanical comparison oftechniques | BMC Musculoskelet Disord | Germany | B |
| 2018 | 30520449 | Economic Analysis of Anatomic Plating Versus Tubular Plating for the Treatmentof Fibula Fractures | Acta Bioeng Biomech | India | A |
| 2018 | 30522801 | The Subacromial Balloon Spacer Versus SuperiorCapsular Reconstruction in the Treatment ofIrreparable Rotator Cuff Tears: A BiomechanicalAssessment | Arthroscopy | Canada | B |
| 2018 | 30527633 | Biomechanical contributions of upper cervical ligamentous structures in TypeII odontoid fractures | J Biomech | USA | A |
| 2018 | 30527634 | The relationship between whole bone stiffness and strength is age and sex dependent | J Biomech | USA | B |
| 2018 | 30529505 | Spring ligament tear decreases static stability of the ankle joint | Clin Biomech (Bristol, Avon) | USA | C |
| 2018 | 30530064 | External loads associated with anterior cruciate ligament injuries increase the correlation between tibial slope and ligament strain during in vitro simulations of in vivo landings | Clin Biomech (Bristol, Avon) | USA | B |
| 2018 | 30554179 | Atlantoaxial fixation using C1 posterior arch screws: feasibility study, morphometric data, and biomechanical analysis | J Neurosurg Spine | USA | C |
| 2018 | 30554907 | Quadriceps augmentation of undersized hamstrings duringACL reconstruction | Knee | USA | C |
| 2018 | 30562831 | Osteochondral Allograft Transplantation: Identifyingthe Biomechanical Impact of Using Shorter Grafts andPulsatile Lavage on Graft Stability | J Knee Surg | USA | B |
| 2018 | 30569208 | Biomechanical evaluation of two minimalaccess interbody cage designs in acadaveric model | J Exp Orthop | The Netherlands | A |
| 2018 | 30579021 | Biomechanical Evaluation of Cervicothoracic Junction Fusion Constructs | World Neurosurg | USA | C |
| 2018 | 30579723 | Mechanical advantage of preserving the hamstring tibial insertion foranterior cruciate ligament reconstruction – A cadaver study | Orthop Traumatol Surg Res | France | B |
| 2018 | 30581129 | Cement augmentation of glenoid baseplate screws does not improveprimary stability in reversed shoulder arthroplasty: A cadaveric study | Orthop Traumatol Surg Res | Germany | A |
| 2018 | 30591261 | Reconstruction of the Superior Glenoid LabrumWith Biceps Tendon Autograft: A CadavericBiomechanical Study | Arthroscopy | USA | C |
| 2018 | 30651948 | Intercondylar Notch Impingement of the Anterior Cruciate Ligament: A Cadaveric In Vitro Study Using Robots | J Healthc Eng | USA | B |
| 2018 | 30675560 | A biomechanical comparison of subscapularis repair techniques intotal shoulder arthroplasty: lesser tuberosity osteotomy versussubscapularis peel | JSES Open Access | USA | A |
| 2018 | 30675596 | A Tensionable Method for Subscapularis Repair after ShoulderArthroplasty | JSES Open Access | USA | C |
| 2018 | 30984495 | Biomechanical Analysis of Cortical VersusPedicle Screw Fixation Stability in TLIF,PLIF, and XLIF Applications | Global Spine J | USA | C |
| 2018 | 30984559 | Asia-Pacific Journal of Sports Medicine, Arthroscopy,Rehabilitation and Technology | Asia Pac J Sports Med Arthrosc Rehabil Technol | Hong Kong | B |
| 2018 | 31019566 | Biomechanical testing of trans-humeralall-suture anchors for rotator cuff repair | Shoulder Elbow | Spain | A |
| 2018 | 31192049 | Scapholunate Ligament Internal Brace 360 Tenodesis(SLITT) Procedure: A Biomechanical Study | J Wrist Surg | USA | A |
| 2018 | 31316585 | The biomechanics of proximalhumeral fractures: Injury mechanismand cortical morphology | Shoulder Elbow | UK | A |
| 2018 | 31463455 | Kinematic assessment of an elastic-core cervical disc prosthesis in one and two-level constructs | JOR Spine | USA | C |
| 2018 | 31513521 | Biomechanics of the Injured Fibula Following Plate Fixation of a Concomitant Tibia Fracture To Fix or Not to Fix? | Bull Hosp Jt Dis (2013) | USA | B |
| 2018 | 32010228 | Long head of biceps tenodesis at thesuperior aspect of the biceps groove:A biomechanical comparison of inlayand onlay techniques | Shoulder Elbow | USA | B |
| 2019 | 29239252 | Single-Bundle vs Double-Bundle (Anatomical) Reconstruction of the Thumb Ulnar Collateral Ligament: Biomechanical Study | Hand (N Y) | USA | A |
| 2019 | 29409299 | Distal fibular malrotation and lateral ankle contact characteristics | Foot Ankle Surg | USA | C |
| 2019 | 29529625 | Femoral Component External Rotation AffectsKnee Biomechanics: A Computational Model ofPosterior-stabilized TKA | Clin Orthop Relat Res | USA | A |
| 2019 | 30075299 | Mechanism of formation of intravertebral clefts in osteoporoticvertebral compression fractures: an in vitro biomechanical study | Spine J | China | C |
| 2019 | 30172718 | Biomechanical Comparison of Intramedullary Beaming and PlantarPlating Methods for Stabilizing the Medial Column of the Foot: An InVitro Study | J Foot Ankle Surg | Canada | B |
| 2019 | 30180091 | Biomechanical Analysis of FixationDevices for Basicervical FemoralNeck Fractures | J Am Acad Orthop Surg | USA | C |
| 2019 | 30181056 | Pullout Strength of All-Suture Anchors:Effect of the Insertion and TractionAngledA Biomechanical Study | Arthroscopy | Korea | C |

| | | | | | |
|---|---|---|---|---|---|
| 2019 | 30234804 | Biomechanical Comparison of C1 LateralMass—C2 Short Pedicle Screw—C3 LateralMass Screw-Rod Construct Versus Goel-HarmsFixation for Atlantoaxial Instability | Spine (Phila Pa 1976) | USA | C |
| 2019 | 30419290 | Iliac screws may not be necessary in long-segment constructs with L5-S1 anterior lumbar interbody fusion: cadaveric study of stability and instrumentation strain | Spine J | USA | C |
| 2019 | 30499627 | Analysis of Differences in Laxities and Neutral Positions from Native after Kinematically Aligned TKA using Cruciate Retaining Implants | J Orthop Res | USA | C |
| 2019 | 30544146 | Disc Geometry Is an Accurate Predictor of LordoticCorrection in the Thoracolumbar Spine FollowingSchwab Grade 2 Osteotomy: A Cadaveric Study andBiomechanical Analysis of Disc Space ChangesFollo | Oper Neurosurg (Hagerstown) | USA | B |
| 2019 | 30562248 | Biomechanical Investigation of an Integrated 2-ScrewCephalomedullary Nail Versus a Sliding Hip Screw inUnstable Intertrochanteric Fractures | J Orthop Trauma | USA | C |
| 2019 | 30611149 | A comprehensive biomechanical analysis of sacral alar iliac fixation: an in vitro human cadaveric model | J Neurosurg Spine | USA | B |
| 2019 | 30611948 | Biomechanical Evaluation of an Anterior Upper Thoracic Plate Fixation System: AnIn Vitro Human Cadaveric Study | World Neurosurg | China | A |
| 2019 | 30612765 | Intra-articular Volume Reduction With ArthroscopicPlication for Capsular Laxity of the Hip: A CadavericComparison of Two Surgical Techniques | Arthroscopy | USA | B |
| 2019 | 30624953 | Biomechanical Comparisonof 3 Novel Repair Techniquesfor Radial Tears of the Medial Meniscus | Am J Sports Med | USA | B |
| 2019 | 30626537 | Effect of the humeral neck-shaft angle and glenosphere lateralization on stability of reverse shoulder arthroplasty: a cadaveric study | J Shoulder Elbow Surg | Germany | B |
| 2019 | 30633081 | Effect of Elbow Position on Load to Failure in OlecranonFracture Fixation: A Biomechanical Cadaveric Study | J Orthop Trauma | USA | C |
| 2019 | 30640515 | Tibial Slope and Its Effect on Forcein Anterior Cruciate Ligament GraftsAnterior Cruciate Ligament Force IncreasesLinearly as Posterior Tibial Slope Increases | Am J Sports Med | USA | B |
| 2019 | 30654770 | Different suture anchor fixation techniquesaffect contact properties in humeral greatertuberosity fracture: a biomechanical study | BMC Musculoskelet Disord | Taiwan | A |
| 2019 | 30658156 | Distal fibula osteotomies improve tibiotalar joint compression: Abiomechanical study in a cadaveric model | Clin Biomech (Bristol, Avon) | USA | B |
| 2019 | 30660211 | Biomechanics of Prophylactic Tethering for Proximal JunctionalKyphosis: Characterization of Spinous Process Tether Pretensioning andPull-Out Force | Spine Deform | USA | C |
| 2019 | 30660212 | Biomechanics of Prophylactic Tethering for Proximal JunctionalKyphosis: Comparison of Posterior Tether Looping Techniques | Spine Deform | USA | C |
| 2019 | 30660397 | Biomechanical Properties of a Novel MeshSuture in a Cadaveric Flexor TendonRepair Model | J Hand Surg Am | USA | C |
| 2019 | 30665370 | Accuracy of a non-invasive CT-basedmeasuring technique for cementpenetration depth in human tibial UKA | BMC Med Imaging | Germany | A |
| 2019 | 30676657 | Effects of tibiofemoral compression on ACL forces and knee kinematics under combined knee loads | J Orthop Res | USA | C |
| 2019 | 30681886 | Kinematics and Laxity of the Ankle Jointin Anatomic and Nonanatomic AnteriorTalofibular Ligament RepairA Biomechanical Cadaveric Study | Am J Sports Med | Japan | A |
| 2019 | 30684758 | Supplemental rods are needed to maximally reduce rod strain acrossthe lumbosacral junction with TLIF but not ALIF in long | Spine J | USA | B |
| 2019 | 30687890 | Slope-reducing tibial osteotomy decreases ACL-graft forces and anterior tibial translation under axial load | Knee Surg Sports Traumatol Arthrosc | Germany | A |
| 2019 | 30688685 | Ultrashort Time to Echo Magnetic Resonance Evaluation ofCalcium Pyrophosphate Crystal Deposition in Human Menisci | Invest Radiol | USA | B |
| 2019 | 30689955 | Biomechanical Comparison of UlnarCollateral Ligament ReconstructionWith a Modified Docking TechniqueWith and Without Suture Augmentation | Am J Sports Med | USA | B |
| 2019 | 30690511 | Sacral Endplate Penetrating Screw for LumbosacralFixation: A Cadaveric Biomechanical Study | Oper Neurosurg (Hagerstown) | USA | B |
| 2019 | 30693406 | Biomechanical effects of osteoplasty withor without Kirschner wire augmentation onlong bone diaphyses undergoing bendingstress: implications for percutaneousimaging-guided consolidation in cancerpatie | Eur Radiol Exp | France | A |
| 2019 | 30694348 | A fluoroscopic analysis of the length changes of the capsulo-osseous layer of the distal iliotibial band | Knee Surg Sports Traumatol Arthrosc | Canada | B |
| 2019 | 30703691 | Novel adhesives for sternal fixation and stabilization: A biomechanicalanalysis | Clin Biomech (Bristol, Avon) | Canada | A |
| 2019 | 30707238 | Comparison of Curved and Straight Anchor Insertion for Bankart Repair | Orthopedics | USA | B |
| 2019 | 30709642 | Strain distribution of the anterolateral ligament during internal rotation atdifferent knee flexion angles: A biomechanical study on human cadavers | Knee | China | A |
| 2019 | 30713066 | A biomechanical comparison of new techniquesfor distal clavicular fracture repair versuslocked plating | J Shoulder Elbow Surg | USA | B |
| 2019 | 30715594 | Education and repetition improve success rate and quantitative measures of the pivot shift test | Knee Surg Sports Traumatol Arthrosc | USA | A |
| 2019 | 30715752 | External Bone Size Is a Key Determinant ofStrength-Decline Trajectories of Aging Male Radii | J Bone Joint Surg Am | USA | B |
| 2019 | 30719479 | Mechanical and Microstructural Propertiesof Pediatric Anterior Cruciate Ligamentsand Autograft Tendons Usedfor Reconstruction | Orthop J Sports Med | USA | B |
| 2019 | 30720558 | Biomechanical Comparison of Tension Band Fixation ofPatella Transverse Fracture: Headless Screws Versus HeadedScrews | J Orthop Trauma | USA | C |
| 2019 | 30723030 | Role of the anconeus in the stability of a lateralligament and common extensor origin–deficientelbow: an in vitro biomechanical study | J Shoulder Elbow Surg | Canada | C |
| 2019 | 30727021 | Biomechanical Properties of BioabsorbableFixation for Osteochondral Shell Allografts | J Knee Surg | USA | B |
| 2019 | 30733036 | A Biomechanical Comparison of Single-, Double-,and Triple-Bundle Anterior Cruciate LigamentReconstructions Using a Hamstring Tendon Graft | Arthroscopy | Japan | A |
| 2019 | 30733037 | Single- Versus Double-Row Repair of Hip AbductorTears: A Biomechanical Matched Cadaver Study | Arthroscopy | USA | B |
| 2019 | 30738222 | Biomechanical testing of different posterior fusion devices onlumbar spinal range of motion | Clin Biomech (Bristol, Avon) | Germany | A |
| 2019 | 30744462 | Comparison of the biomechanics ofradial head prostheses with dynamicloading in the radiocapitellar joint | J Hand Surg Eur Vol | Korea | C |
| 2019 | 30747259 | Design and preliminary biomechanical analysis of a novel motion preservation device for lumbar spinal disease after vertebral corpectomy | Arch Orthop Trauma Surg | China | A |
| 2019 | 30762217 | Biomechanical comparison of the three techniques for arthroscopic suprapectoral biceps tenodesis: implant-free intraosseous tendon fxation with Cobra Guide, interference screw and suture anchor | Musculoskelet Surg | Slovenia | C |
| 2019 | 30765309 | Biomechanical properties of transosseous bony Bankart repair in acadaver model | Orthop Traumatol Surg Res | France | B |
| 2019 | 30770316 | Parameterization of proximal humerus locking plate impingement with in vitro, in silico, and in vivo techniques | J Shoulder Elbow Surg | USA | A |
| 2019 | 30783606 | Proximal Hamstring RepairA Biomechanical Analysis ofVariable Suture Anchor Constructs | Orthop J Sports Med | USA | B |
| 2019 | 30783803 | A new variant ligament of the atlantooccipital joint: the lateral oblique atlantooccipital ligament | Eur Spine J | USA | B |
| 2019 | 30784787 | Screws with larger core diameter and lower thread pitch increase thestability of locked plating in osteoporotic proximal humeral fractures | Clin Biomech (Bristol, Avon) | Germany | A |
| 2019 | 30793358 | In Vitro Injection of Osteoporotic Cadaveric Femurs with a Triphasic Calcium-Based Implant Confers Immediate Biomechanical Integrity | J Orthop Res | USA | C |
| 2019 | 30797975 | Superior biceps aponeurosis'-Morphological characteristics of the origin of the short head of the biceps brachii muscle | Ann Anat | Poland | A |
| 2019 | 30798770 | Biomechanical consequences of firstmetatarsaophalangeal joint arthrodesison flexor digitorum longus function:A cadaveric study | J Orthop Surg (Hong Kong) | Singapore | C |
| 2019 | 30799174 | Superior capsular reconstruction for irreparable supraspinatus tendontears using the long head of biceps: A biomechanical study oncadavers | Orthop Traumatol Surg Res | Singapore | C |
| 2019 | 30800688 | Effect of Lower Limb Alignmentin Medial Meniscus–Deficient Kneeson Tibiofemoral Contact Pressure | Orthop J Sports Med | Germany | B |
| 2019 | 30800692 | The Middle and Distal Aspects of the UlnarFootprint of the Medial Ulnar CollateralLigament of the Elbow Do Not ProvideSignificant Resistance to Valgus Stress | Orthop J Sports Med | USA | A |
| 2019 | 30804131 | Cadaveric Study on Retrograde Calcaneotibial Fixation of Unstable Ankle Fractures | In Vivo | Germany | C |
| 2019 | 30813805 | A Biomechanical Comparison of Fiberglass Casts and 3-Dimensional–Printed, OpenLatticed, Ventilated Casts | Hand (N Y) | USA | B |
| 2019 | 30815344 | A comparative biomechanical evaluation of different osteosynthesistechniques used for intracapsular condylar head fractures | J Oral Biol Craniofac Res | Ukraine | A |
| 2019 | 30822130 | Biomechanical Evaluation of Spring Ligament Augmentation With the FiberTape Device in a Cadaveric Flatfoot Model | Foot Ankle Int | USA | C |
| 2019 | 30845842 | Fowler Central Slip Tenotomy or Spiral Oblique Retinacular Ligament Reconstruction? A Cadaveric Biomechanical Study in Swan-Neck Deformity | Hand (N Y) | USA | A |
| 2019 | 30849746 | Cortical bone trajectory screws used to save failed traditional trajectory screws in the osteoporotic lumbar spine and vice versa: a human cadaveric biomechanical study | J Neurosurg Spine | China | A |
| 2019 | 30859610 | Non-uniform strain distribution in anterolateral capsule of knee: Implications for surgical repair | J Orthop Res | USA | A |
| 2019 | 30865838 | The Role of Anterior CapsularLaxity in Hip MicroinstabilityA Novel Biomechanical Model | Am J Sports Med | USA | B |
| 2019 | 30871405 | The effects of incremental ulnar styloidosteotomies on distal radioulnar jointstability: a biomechanical cadaveric study | J Hand Surg Eur Vol | USA | C |
| 2019 | 30876933 | Anatomic considerations in headaches associated with cervical sagittalimbalance: A cadaveric biomechanical study | J Clin Neurosci | USA | C |

| | | | | | |
|---|---|---|---|---|---|
| 2019 | 30876999 | Biomechanical Effects of an Oblique Lumbar PEEK Cage and Posterior Augmentation | World Neurosurg | USA | C |
| 2019 | 30880450 | Biomechanical Comparison of Low-Profile Contoured Locking Plate With Single Compression Screw to Fully Threaded Compression Screws for First MTP Fusion | Foot Ankle Int | USA | A |
| 2019 | 30880489 | Trapeziometacarpal Fusion With and Without Distal Scaphoid Excision: A Cadaveric Model Comparing Thumb Range of Motion | Hand (N Y) | USA | A |
| 2019 | 30888526 | Influence of knee position and examiner-induced motion on the kinematics of the pivot shift | J Exp Orthop | USA | A |
| 2019 | 30889432 | How does a novel knitted titanium nucleus prosthesis changethe kinematics of a cervical spine segment? A biomechanicalcadaveric study | Clin Biomech (Bristol, Avon) | Germany | C |
| 2019 | 30891387 | Anatomico-radiological Study of theBifurcate Ligament of the Foot withClinical Significance | Cureus | USA | C |
| 2019 | 30893221 | A New Fastener With Improved Bone-To-Implant InterfaceShows Superior Torque Stripping Resistance ComparedWith the Standard Buttress Screw | J Orthop Trauma | USA | A |
| 2019 | 30894097 | An experimental evaluation of fracturemovement in two alternative tibialfracture fixation models using avibrating platform | Proc Inst Mech Eng H | UK | A |
| 2019 | 30896980 | Tibial Slope and Its Effect on GraftForce in Posterior Cruciate LigamentReconstructions | Am J Sports Med | USA | B |
| 2019 | 30897004 | Biomechanical Comparison of VerticalMattress and Cross-stitch SutureTechniques and Single- and Double-RowConfigurations for the Treatmentof Bucket-Handle Medial Meniscal Tears | Am J Sports Med | USA | B |
| 2019 | 30901470 | Characterization of Protection Mechanisms to Blast Overpressurefor Personal Hearing Protection Devices – BiomechanicalMeasurement and Computational Modeling | Mil Med | USA | B |
| 2019 | 30902534 | A Superolaterally Placed Anchor for Subscapularis“Leading-Edge” Refixation: A Biomechanical Study | Arthroscopy | Germany | B |
| 2019 | 30902593 | A biomechanical cadaveric study of patellartendon allograft as an alternative graft materialfor superior capsule reconstruction | J Shoulder Elbow Surg | USA | B |
| 2019 | 30904752 | The stabilization effect of the conjoint tendon in reverse totalshoulder arthroplasty | Clin Biomech (Bristol, Avon) | Germany | A |
| 2019 | 30905000 | Laparoscopic pectopexy: a follow-up cyclic biomechanical analysis determining time to functional stability | Arch Gynecol Obstet | Germany | B |
| 2019 | 30909782 | Effects of humeral shortening on thethree-dimensional configuration of thebrachial plexus: a cadaveric study | J Hand Surg Eur Vol | Belgium | C |
| 2019 | 30917671 | Biomechanical Comparison of Suture-External Button Fixation vs Internal Suspension Fixation for Tendon Transfers of the Feet | Foot Ankle Int | USA | A |
| 2019 | 30917910 | In vitro determination of the passive knee flexion axis: Effects of axisalignment on coupled tibiofemoral motions | Med Eng Phys | USA | B |
| 2019 | 30923984 | New Findings Confirm Regional Internal Disc Strain Changes During Simulation of Repetitive Lifting Motions | Ann Biomed Eng | Australia | A |
| 2019 | 30926192 | A Biomechanical Study of the Role of theAnterolateral Ligament and the Deep Iliotibial Bandfor Control of a Simulated Pivot Shift WithComparison of Minimally Invasive Extra-articularAnterolateral Tendc | Arthroscopy | USA | B |
| 2019 | 30929480 | Hip capsule biomechanics after arthroplastyTHE EFFECT OF IMPLANT, APPROACH, AND SURGICAL REPAIR | Bone Joint J | UK | A |
| 2019 | 30937322 | Comparison of Graft Length Changes DuringKnee Motion Among 5 Different AnatomicSingle-Bundle Anterior Cruciate LigamentReconstruction Approaches | Orthop J Sports Med | Japan | A |
| 2019 | 30941252 | Carpal Kinematics following SequentialScapholunate Ligament Sectioning | J Wrist Surg | Canada | C |
| 2019 | 30954064 | Proximal radius fracture morphologyfollowing axial force impact: abiomechanical evaluation of fracturepatterns | BMC Musculoskelet Disord | Germany | A |
| 2019 | 30964697 | Dynamic Anterior Shoulder StabilizationWith the Long Head of the Biceps TendonA Biomechanical Study | Am J Sports Med | USA | B |
| 2019 | 30978679 | Tailoring selection of transforaminal interbody spacers based on biomechanical characteristics and surgical goals: evaluation of an expandable spacer | J Neurosurg Spine | USA | C |
| 2019 | 30987529 | Motion of the distal tibiofibularsyndesmosis under different loadingpatterns: A biomechanical study | J Orthop Surg (Hong Kong) | China | A |
| 2019 | 30989797 | Comparison of Less Invasive Stabilization SystemPlate and Retrograde Intramedullary Nail in theFixation of Femoral Supracondylar Fractures in theElderly: A Biomechanical Study | Orthop Surg | China | A |
| 2019 | 30989818 | Sex and Laterality Differences inMedullary Humerus Morphology | Anat Rec (Hoboken) | USA | B |
| 2019 | 30998643 | Cannulated Screw Prominence in Tension Band Wiring of PatellaFractures Increases Fracture Gapping: A Cadaver Study | Clin Orthop Relat Res | USA | A |
| 2019 | 31000387 | Biomechanical Analysis of Medial-Row All-SutureSuture Anchor Fixation for Rotator Cuff Repair in aPair-Matched Cadaveric Model | Arthroscopy | USA | B |
| 2019 | 31006644 | Kinematically aligned total knee arthroplasty reproduces more nativerollback and laxity than mechanically aligned total knee arthroplasty:A matched pair cadaveric study | Orthop Traumatol Surg Res | Korea | B |
| 2019 | 31018860 | Biomechanical investigation of dynamic hip screw and wire fxation on an unstable intertrochanteric fracture | Biomed Eng Online | Taiwan | C |
| 2019 | 31019985 | A Biomechanical Comparisonof the Arciero and LaPrade Reconstructionfor Posterolateral Corner Knee Injuries | Orthop J Sports Med | USA | B |
| 2019 | 31024965 | Acromioclavicular Joint StabilizationA Biomechanical Study of Bidirectional Stability and Strength | Orthop J Sports Med | Australia | A |
| 2019 | 31026649 | Near infrared spectroscopy enables quantitative evaluation of human cartilagebiomechanical properties during arthroscopy | Osteoarthritis Cartilage | Finland | A |
| 2019 | 31029520 | Biomechanical analysis of latissimus dorsitendon transfer with and without superiorcapsule reconstruction using dermal allograft | J Shoulder Elbow Surg | USA | B |
| 2019 | 31031148 | Posterolateral plate fixation with Pantalarlock1 is more stable than nailfixation in tibiotalocalcaneal arthrodesis in a biomechanical cadaverstudy | Foot Ankle Surg | Germany | C |
| 2019 | 31035887 | Benefits of Additional Procedures for Metacarpophalangeal Hyperextension on Simple Trapeziectomy in Thumb Basal Osteoarthritis: A Biomechanical Cadaver Study | J Hand Surg Asian Pac Vol | Switzerland | A |
| 2019 | 31042314 | Associations between intervertebral disc degeneration grading schemes and measures of disc function | J Orthop Res | UK | C |
| 2019 | 31042658 | The biomechanical effect of single-level laminectomy and posterior instrumentation on spinal stability in degenerative lumbar scoliosis: a human cadaveric study | Neurosurg Focus | The Netherlands | C |
| 2019 | 31051462 | Biomechanical evaluation of traditional posterior versus anterior spondylolisthesis reduction in a cadaveric grade I slip model | J Neurosurg Spine | USA | C |
| 2019 | 31053390 | Ulnar collateral ligament insufficiency affectscubital tunnel syndrome during throwingmotion: a cadaveric biomechanical study | J Shoulder Elbow Surg | Japan | B |
| 2019 | 31054437 | The use of a hydrogel implant in the repair of osteochondraldefects of the knee: A biomechanical evaluation of restoration ofnative contact pressures in cadaver knees | Clin Biomech (Bristol, Avon) | USA | B |
| 2019 | 31059851 | A Biomechanical Analysis of Lateral Interbody Construct and Supplemental Fixation inAdjacent-Segment Disease of the Lumbar Spine | World Neurosurg | USA | B |
| 2019 | 31059899 | Differences between human septal and alar cartilage with respect tobiomechanical features and biochemical composition | J Mech Behav Biomed Mater | Germany | A |
| 2019 | 31064313 | Biomechanical in vitro evaluation of aready-to-use calcium phosphatecement implanted to augmentintramedullary nail fixation of a three  part humeral head fracture model | Proc Inst Mech Eng H | Germany | C |
| 2019 | 31072716 | Biomechanical Analysis of All-Suture Suture AnchorFixation Compared With Conventional SutureAnchors and Interference Screws for BicepsTenodesis | Arthroscopy | USA | B |
| 2019 | 31075567 | Experimental investigation of cranial fracture initiation in blunt humanhead impacts | Forensic Sci Int | USA | C |
| 2019 | 31075766 | Biomechanical implications of unilateral facetectomy, unilateral facetectomy plus partial contralateral facetectomy, and complete bilateral facetectomy in minimally invasive transforaminal interbody fusion | J Neurosurg Spine | USA | C |
| 2019 | 31077171 | Initial stability of one-stage anteriordebridement and cage implantationcombined with anterior-lateral fixation by adual screw-rod construct in the treatmentof lumbosacral tuberculosis: a cadavericbiomechan | BMC Musculoskelet Disord | China | A |
| 2019 | 31078898 | The effect of torsional moments on the posterolateral rotatory stability of alateral ligament deficient elbow: An in vitro biomechanical investigation | Clin Biomech (Bristol, Avon) | Canada | C |
| 2019 | 31091129 | Utilization of Transtibial Centralization Suture Best Minimizes Extrusion and Restores Tibiofemoral Contact Mechanics for Anatomic Medial Meniscal Root Repairs in a Cadaveric Model | Am J Sports Med | USA | B |
| 2019 | 31101434 | Stem Length and Neck Resection on Fixation Strength of Press-Fit Radial Head Prosthesis: An In Vitro Model | J Hand Surg Am | USA | A |
| 2019 | 31125537 | Temporomandibular Joint Condyle-Disc Morphometric Sexual Dimorphisms Independent of Skull Scaling | J Oral Maxillofac Surg | USA | A |
| 2019 | 31135207 | Kinematic and Kinetic Comparison of Fresh Frozen andThiel-Embalmed Human Feet for Suitability forBiomechanical Educational and Research Settings | J Am Podiatr Med Assoc | Malta | A |
| 2019 | 31136372 | Ligamentous Lisfranc Injury: A Biomechanical Comparisonof Dorsal Plate Fixation and Transarticular Screws | J Orthop Trauma | USA | C |
| 2019 | 31150273 | Multiplanar Loading of the Knee and Its Influence on Anterior Cruciate Ligament and Medial Collateral Ligament Strain During Simulated Landings and Noncontact Tears | Am J Sports Med | USA | B |
| 2019 | 31151003 | Injury risk of interphalangeal and metacarpophalangeal joints under impact loading | J Mech Behav Biomed Mater | UK | A |
| 2019 | 31156021 | The effect of cup orientation onstability of trapeziometacarpal totaljoint arthroplasty: a biomechanicalcadaver study | J Hand Surg Eur Vol | Belgium | C |
| 2019 | 31169580 | Biomechanics of Biodegradable Subacromial Balloon Spacer for Irreparable Superior Rotator Cuff Tears: Study of a Cadaveric Model | J Bone Joint Surg Am | USA | C |
| 2019 | 31171375 | An In Vitro Study to Determine the Effect of Ulnar Shortening on Distal Forearm Loading During Wrist and Forearm Motion: Implications in the Treatment of Ulnocarpal Impaction | J Hand Surg Am | Canada | C |
| 2019 | 31179742 | Effect of Metacarpal Fixation Configuration on Strength and Fracture Gap Displacements in a Cadaveric Model of Externally Fixed Distal Radius Fractures | Hand (N Y) | USA | A |
| 2019 | 31183297 | Adjustable Rigid Interspinous Process Fixation: ABiomechanical Study of Segmental Lordosis andInterbody Loading in the Lumbar Spine | Cureus | USA | C |

| | | | | | |
|---|---|---|---|---|---|
| 2019 | 31183426 | Biomechanical evaluation ofperipheral nerves after crushinjuries | Heliyon | Singapore | B |
| 2019 | 31197751 | Biomechanical analysis of proximal tibia bone grafting and the effectof the size of osteotomy using a validated finite element model | Med Biol Eng Comput | Singapore | C |
| 2019 | 31200368 | A biomechanical investigation of the sacroiliac joint in the setting of lumbosacral fusion: impact of pelvic fixation versus sacroiliac joint fixation | J Neurosurg Spine | USA | C |
| 2019 | 31203433 | A biomechanical comparison between human calvarial boneand a skull simulant considering the role of attached periosteumand dura mater | Int J Legal Med | Germany | A |
| 2019 | 31203670 | Biomechanical Comparison of Achilles Tendon Pullout Strength Following Midline Tendon-Splitting and Endoscopic Approaches for Calcaneoplasty | Foot Ankle Int | USA | C |
| 2019 | 31204043 | Central screw use delays implant dislodgement in osteopenic bone butnot synthetic surrogates: A comparison of reverse total shoulder models | J Biomech | USA | C |
| 2019 | 31206794 | Condylar-Stabilized TKR May Not Fully Compensate for PCL-Deficiency: An In Vitro Cadaver Study | Orthop Res | Canada | C |
| 2019 | 31212212 | Influence of additional cement augmentation on endplate stability in circumferential stabilisation of osteoporotic spine fractures | Clin Biomech (Bristol, Avon) | Germany | A |
| 2019 | 31218236 | Is Arthroscopic TransosseousRotator Cuff Repair StrengthDependent on the Tunnel Angle? | Orthop J Sports Med | USA | B |
| 2019 | 31220025 | Tibiofemoral Kinematics During CompressiveLoading of the ACL-Intact and ACL-Sectioned Knee | J Bone Joint Surg Am | USA | C |
| 2019 | 31233339 | Biomechanical Assessment of a DistallyFixed Lateral Extra-articular AugmentationProcedure in the Treatment of AnterolateralRotational Laxity of the Knee | Am J Sports Med | Australia | A |
| 2019 | 31234032 | Biomechanical analysis of motion following sacroiliac jointfusion using lateral sacroiliac screws with or without lumbosacralinstrumented fusion | Clin Biomech (Bristol, Avon) | USA | C |
| 2019 | 31238779 | Extensor pollicis longus rerouting forthumb-in-palm deformity in cerebralpalsy: a biomechanical analysis | J Hand Surg Eur Vol | The Netherlands | C |
| 2019 | 31250225 | Suture anchor fxation strength in the Latarjet procedure: a biomechanical study in cadavers | Eur J Orthop Surg Traumatol | Turkey | A |
| 2019 | 31254026 | Glenoid retroversion is an important factor for humeral head centration and the biomechanics of posterior shoulder stability | Knee Surg Sports Traumatol Arthrosc | Germany | A |
| 2019 | 31256217 | Pressure changes in the Kager fat pad at the extremes of ankle motion suggest a potential role in Achilles tendinopathy | Knee Surg Sports Traumatol Arthrosc | UK | A |
| 2019 | 31256663 | A calcar collar is protective against early periprosthetic femoral fracture around cementless femoral components in primary total hip arthroplasty | Bone Joint J | UK | A |
| 2019 | 31260520 | Regional Variations ofHR-pQCT Morphological andBiomechanical Measurementsof Bone Segments and TheirAssociations With WholeDistal Radius and TibiaMechanical Properties | J Biomech Eng | USA | B |
| 2019 | 31260844 | The influence of selective arthrodesis on three-dimensional range of motionof hindfoot joint: A cadaveric study | Clin Biomech (Bristol, Avon) | China | A |
| 2019 | 31263532 | Novel simulations to determine the impact of superficial perineal structures on vaginal delivery | Interface Focus | USA | B |
| 2019 | 31270628 | Strain behavior of native and reconstructed medial patellofemoral ligaments during dynamic knee flexion - a cadaveric study | J Exp Orthop | Singapore | C |
| 2019 | 31272890 | The effect of the subacromial balloon spaceron humeral head translation in the treatmentof massive, irreparable rotator cuff tears:a biomechanical assessment | J Shoulder Elbow Surg | Canada | B |
| 2019 | 31274195 | Identification of the retrotalar pulley of the Flexor Hallucis Longus tendon | J Anat | Switzerland | A |
| 2019 | 31280279 | Influence of Deviated Centers of Rotation on Kinematics and Kinetics of a Lumbar Functional Spinal Unit: An In Vitro Study | Med Sci Monit | China | C |
| 2019 | 31284809 | A biomechanical comparison of themodified Bunnell pullout and Teointraosseous suture techniques forattachment of tendon to bone | J Hand Surg Eur Vol | Spain | A |
| 2019 | 31288211 | Biomechanical behaviour of human bile duct wall and impact of cadavericpreservation processes | J Mech Behav Biomed Mater | France | A |
| 2019 | 31295670 | Influence of relative injury risk profiles on anterior cruciateligament and medial collateral ligament strain during simulatedlanding leading to a noncontact injury event | Clin Biomech (Bristol, Avon) | USA | C |
| 2019 | 31309016 | The Future of Biomechanical SpineResearch: Conception and Design of aDynamic 3D Printed Cervical MyelographyPhantom | Cureus | USA | B |
| 2019 | 31309770 | Anatomic Considerations of Anterior Transarticular Screw Fixation for Atlantoaxial Instability | Asian Spine J | India | A |
| 2019 | 31311681 | Image-Based Comparison Between theBilateral Symmetry of the Distal RadiiThrough Established Measures | J Hand Surg Am | Canada | C |
| 2019 | 31311747 | Biomechanical analysis of conventional anchorrevision after all-suture anchor pullout: a humancadaveric shoulder model | J Shoulder Elbow Surg | Germany | A |
| 2019 | 31314890 | Characterization of Ankle Kinematics and Constraint Following Ligament Rupture in a Cadaveric Model | J Biomech Eng | USA | C |
| 2019 | 31314996 | Lateral Meniscal Graft Transplantation: Effect of Fixation Method on Joint Contact Mechanics During Simulated Gait | Am J Sports Med | USA | A |
| 2019 | 31318812 | A Novel Treatment for Anterior Shoulder InstabilityA Biomechanical Comparison Between a Patient-Specific Implantand the Latarjet Procedure | J Bone Joint Surg Am | The Netherlands | C |
| 2019 | 31322918 | Biomechanical Comparison of OnlayDistal Biceps Tendon RepairAll-Suture Anchors Versus Titanium Suture Anchors | Am J Sports Med | USA | B |
| 2019 | 31326631 | Material properties of human lumbar intervertebral discs across strain rates | Spine J | UK | A |
| 2019 | 31330318 | Investigation on distal femoral strength and reconstruction failure following curettageand cementation: In-vitro tests with finite element analyses | Comput Biol Med | Iran | A |
| 2019 | 31331831 | Assessment of proximal femurmicroarchitecture using ultra-high field MRIat 7 Tesla | Diagn Interv Imaging | France | C |
| 2019 | 31334432 | Biomechanics of an interlinked suture anchor rotator cuff repairin a human cadaveric model | JSES Open Access | USA | B |
| 2019 | 31335566 | Biomechanical Analysis of Instability in Rotational DistalFibula Fractures (OTA/AO 44-B1) With an Intact DeltoidLigament | J Orthop Trauma | USA | C |
| 2019 | 31345755 | Dual Nonlocked Plating as an Alternative to Locked Plating for ComminutedDistal Fibula Fractures: A Biomechanical Comparison Study | J Foot Ankle Surg | USA | B |
| 2019 | 31350928 | Secondary perforation risk in plate osteosynthesis of unstable proximal humerus fractures: a biomechanical investigation of the effect of screw length | J Orthop Res | Switzerland | B |
| 2019 | 31351903 | Biomechanical comparison of docking ulnarcollateral ligament reconstruction with andwithout an internal brace | J Shoulder Elbow Surg | USA | B |
| 2019 | 31352256 | Alterations in tibiotalar joint reaction force following syndesmotic injury arerestored with static syndesmotic fixation | Clin Biomech (Bristol, Avon) | USA | C |
| 2019 | 31358284 | The response of the pediatric head to impacts onto a rigid surface | J Biomech | USA | C |
| 2019 | 31359101 | The CFL fails before the ATFL immediately after combined ligament repair in a biomechanical cadaveric model | Knee Surg Sports Traumatol Arthrosc | USA | B |
| 2019 | 31372372 | MRI-derived bone porosity index correlates to bone composition andmechanical stiffness | Bone Rep | USA | C |
| 2019 | 31377827 | Varus alignment aggravates tibiofemoral contact pressure rise after sequential medial meniscus resection | Knee Surg Sports Traumatol Arthrosc | Germany | A |
| 2019 | 31381174 | Digital Tomosynthesis Based Digital Volume Correlation: A Clinically Viable Noninvasive Method for Direct Measurement of Intra-Vertebral Displacements Using Images of the Human Spine Under Physiol | Med Phys | USA | A |
| 2019 | 31381363 | Low-Profile Dual Small Plate FixationIs Biomechanically Similar to LargerSuperior or Anteroinferior Single PlateFixation of Midshaft Clavicle Fractures | Am J Sports Med | USA | B |
| 2019 | 31386563 | Establishing Safe Extra-articularParameters for Cortical ButtonDistal Fixation During Ulnar CollateralLigament Reconstruction | Am J Sports Med | USA | B |
| 2019 | 31395180 | Influence of Medial Meniscus Bucket-Handle Repairin Setting of Anterior Cruciate LigamentReconstruction on Tibiofemoral Contact Mechanics:A Biomechanical Study | Arthroscopy | USA | B |
| 2019 | 31395394 | The 6-O'clock Anchor Increases Labral RepairStrength in a Biomechanical Shoulder InstabilityModel | Arthroscopy | USA | C |
| 2019 | 31399010 | Reconstruction of the forearminterosseous membrane: a biomechanicalstudy of three different techniques | J Hand Surg Eur Vol | Greece | C |
| 2019 | 31410528 | Repair of the medial patellofemoral ligament with suture tape augmentation leads to similar primary contact pressures and joint kinematics like reconstruction with a tendon graft: a biomechanical compariso | Knee Surg Sports Traumatol Arthrosc | USA | B |
| 2019 | 31410883 | A Robotically Simulated Pivot Shift That Represents the Clinical Exam | J Orthop Res | USA | C |
| 2019 | 31412206 | Portal Placement and BiomechanicalPerformance of EndoscopicProximal Hamstring Repair | Am J Sports Med | USA | C |
| 2019 | 31414289 | Biomechanical in vitro comparison between anterior column realignment and pedicle subtraction osteotomy for severe sagittal imbalance correction | Eur Spine J | Italy | B |
| 2019 | 31420224 | Posterior shoulder tightness can be a risk factorof scapular malposition: a cadavericbiomechanical study | J Shoulder Elbow Surg | Japan | A |
| 2019 | 31420689 | Acromioclavicular joint reconstruction: an additional acromioclavicular cerclage does not improve horizontal stability in double coraco-clavicular tunnel technique | Knee Surg Sports Traumatol Arthrosc | Germany | A |
| 2019 | 31421939 | The Biomechanical Consequences ofTrapeziectomy and Partial Trapezoidectomyin the Treatment of Thumb Carpometacarpaland Scaphotrapeziotrapezoid Arthritis | J Hand Surg Am | USA | A |
| 2019 | 31427244 | Biomechanical comparison of anterolateral ligament anatomicalreconstruction with a semi-anatomical lateral extra-articular tenodesis. Acadaveric study | Knee | Spain | A |
| 2019 | 31431075 | Biomechanical Analysis of Thumb Ulnar Collateral Ligament Tear Kinematics | Hand (N Y) | USA | B |
| 2019 | 31436659 | Flexor Hallucis Longus Transfer ImprovesAchilles Tendon Load to Failure in Surgeryfor Non-Insertional Tendinopathy | J Bone Joint Surg Am | USA | B |

| | | | | | |
|---|---|---|---|---|---|
| 2019 | 31444834 | Side Variations of Anterior Cruciate Ligament Coronal Angles: Implications for ACL Reconstruction | Clin Anat | USA | A |
| 2019 | 31445401 | Exploring the pathological role of intervertebral disc and facet joint in thedevelopment of degenerative scoliosis by biomechanical methods | Clin Biomech (Bristol, Avon) | China | A |
| 2019 | 31449561 | Cadaveric biomechanical testing of torque -to - failure magnitude of Bilateral ApicalVertebral Derotation maneuver in the thoracicspine | PLoS One | Poland | A |
| 2019 | 31451321 | Approach to the Perpendicular Fixation ofa Scaphoid Waist Fracture—A ComputerAnalyzed Cadaver Model | J Hand Surg Am | Israel | C |
| 2019 | 31454255 | Bone-Plug Versus Soft Tissue Fixationof Medial Meniscal Allograft TransplantsA Biomechanical Study | Am J Sports Med | Brazil | B |
| 2019 | 31454261 | Posteromedial Ligament Repair of the KneeWith Suture Tape AugmentationA Biomechanical Study | Am J Sports Med | Germany | B |
| 2019 | 31461303 | The Effectiveness of a HingedElbow Orthosis in Medial CollateralLigament InjuriesAn In Vitro Biomechanical Study | Am J Sports Med | Canada | A |
| 2019 | 31462088 | Proximal and Distal Failure Site Analysis in Percutaneous Achilles Tendon Rupture Repair | Foot Ankle Int | Chile | C |
| 2019 | 31463604 | Biomechanical analysis of anterior pelvic ring fractures with intact peripelvic soft tissues: a cadaveric study | Eur J Trauma Emerg Surg | Germany | A |
| 2019 | 31463689 | Biomechanical evaluation of suture buttons versus cortical screws in the Latarjet–Bristow procedure: a fresh-frozen cadavers study | Arch Orthop Trauma Surg | Israel | C |
| 2019 | 31479817 | Mechanical testing setups affect spine segment fracture outcomes | J Mech Behav Biomed Mater | USA | A |
| 2019 | 31480097 | Biomechanical performance of an intramedullary Echidna pinfor fixation of comminuted mid-shaft clavicle fractures | ANZ J Surg | Australia | A |
| 2019 | 31489328 | Irreparable Rotator Cuff TearsA Biomechanical Comparison of SuperiorCapsuloligamentous Complex ReconstructionTechniques and an Interpositional Graft Technique | Orthop J Sports Med | Germany | C |
| 2019 | 31496035 | Mechanical Properties of the HumanTibial and Peroneal Nerves FollowingStretch with Histological Correlations | Anat Rec (Hoboken) | USA | B |
| 2019 | 31502697 | Anteroposterior Translational Malalignment of Ankle Arthrodesis Alters Foot Biomechanics in Cadaveric Gait Simulation | J Orthop Res | USA | C |
| 2019 | 31512228 | Quantification of the regional bioarchitecture in the human aorta | J Anat | Ireland | C |
| 2019 | 31518683 | Optimizing biomechanics of anterior column realignment forminimally invasive deformity correction | Spine J | USA | B |
| 2019 | 31522530 | Knot of Henry Variation and the Effect on Plantar Flexion Strength | Foot Ankle Int | USA | B |
| 2019 | 31526590 | Protocol development for synchrotron contrast-enhanced CT of human hip cartilage | Med Eng Phys | Canada | A |
| 2019 | 31547966 | Ankle joint contact loads and displacement in syndesmosis injuries repaired with Tightropes compared to screw fixation in a static model | Injury | USA | C |
| 2019 | 31552149 | Single-Level In Vitro Kinematic Comparisonof Novel Inline Cervical Interbody DevicesWith Intervertebral Screw, Anchor, or Blade | Global Spine J | USA | C |
| 2019 | 31559166 | Comparison of cartilage and bone morphological models of the ankle joint derived from different medical imaging technologies | Quant Imaging Med Surg | Italy | C |
| 2019 | 31559489 | Modifed suture-bridge technique for tibial avulsion fractures of the posterior cruciate ligament: a biomechanical comparison | Arch Orthop Trauma Surg | Germany | B |
| 2019 | 31560563 | Effect of Meniscal Ramp Lesion Repairon Knee Kinematics, Bony ContactForces, and In Situ Forces in the AnteriorCruciate Ligament | Am J Sports Med | USA | B |
| 2019 | 31581315 | Load–Deformation Properties of the Ligamentof the Head of Femur In Situ | Clin Anat | New Zealand | A |
| 2019 | 31594727 | Biomechanical analysis of anterior capsulereconstruction and latissimus dorsi transferfor irreparable subscapularis tears | J Shoulder Elbow Surg | USA | B |
| 2019 | 31606762 | Minimally invasive reconstruction technique for chronic Achilles tendon tears allows rapid return to walking and leads to good functional recovery | Knee Surg Sports Traumatol Arthrosc | Poland | A |
| 2019 | 31620486 | Biomechanical Evaluation of a ModifiedInternal Brace Construct for the Treatmentof Ulnar Collateral Ligament Injuries | Orthop J Sports Med | USA | B |
| 2019 | 31624904 | The novel arthroscopic subscapular sling procedure grants better stability than an arthroscopic Bankart repair in a cadaveric study | Knee Surg Sports Traumatol Arthrosc | Norway | A |
| 2019 | 31628296 | Analysis of adjacent-segment cervical kinematics: the role of construct length and the dorsal ligamentous complex | J Neurosurg Spine | USA | C |
| 2019 | 31629583 | Anterior Capsule Reconstruction Versus PectoralisMajor Transfer for Irreparable Subscapularis TearsInvolving the Anterior Capsule: A ComparativeBiomechanical Cadaveric Study | Arthroscopy | USA | B |
| 2019 | 31632997 | Different Suture Materials for ArthroscopicTranstibial Pull-out Repair of MedialMeniscal Posterior Root Tears | Orthop J Sports Med | USA | B |
| 2019 | 31634616 | Biomechanical Evaluation Comparing Zero-Profile Devices Versus Fixed Profile Systems in a Cervical Hybrid Decompression Model A Biomechanical In Vitro Study | Spine J | USA | B |
| 2019 | 31647881 | Anatomic and Biomechanical Evaluationof Ulnar Tunnel Position in Medial UlnarCollateral Ligament Reconstruction | Am J Sports Med | USA | B |
| 2019 | 31663008 | Biomechanical Comparison of SubpectoralBiceps Tenodesis Onlay Techniques | Orthop J Sports Med | USA | C |
| 2019 | 31667197 | Biomechanical Comparison of Quadricepsand 6-Strand Hamstring Tendon Graftsin Anterior Cruciate Ligament Reconstruction | Orthop J Sports Med | USA | B |
| 2019 | 31668412 | Study of intraarticular pressures in the elbow joints | J Biomech | Spain | C |
| 2019 | 31668957 | The Role of Far Cortical Endosteal Fixation for the Treatment of MedialMalleolus Fractures: A Biomechanical Study | J Foot Ankle Surg | USA | C |
| 2019 | 31669919 | Stabilizing effect of the rib cage on adjacent segment motion followingthoracolumbar posterior fixation of the human thoracic cadaveric spine: Abiomechanical study | Clin Biomech (Bristol, Avon) | USA | A |
| 2019 | 31670389 | Visualization of intervertebral disc degeneration in a cadaveric human lumbar spine using microcomputed tomography | J Anat | Austria | B |
| 2019 | 31678445 | Anterior Lumbar Interbody Fusion May Provide Superior Decompression of theForaminal Space Compared with Direct Foraminotomy: Biomechanical CadavericStudy | World Neurosurg | USA | A |
| 2019 | 31679831 | Comparison of three fixation techniques for arcuate fractures | Injury | USA | C |
| 2019 | 31686711 | Thick graft vs. double-bundle technique onposterior cruciate ligament reconstruction:experimental biomechanical study with cadavers | Rev Bras Ortop (Sao Paulo) | Brazil | A |
| 2019 | 31687789 | dGEMRIC and CECT Comparison of Cationic and Anionic ContrastAgents in Cadaveric Human Metacarpal Cartilage | J Orthop Res | USA | B |
| 2019 | 31689815 | Effect of soft tissue injury and ulnar angulation onradial head instability in a Bado type I Monteggiafracture model | Medicine (Baltimore) | Japan | A |
| 2019 | 31693747 | Biomechanical Effects of Fiber Patch Augmentation on Rotator Cuff Repairs | Orthopedics | USA | C |
| 2019 | 31702442 | Biomechanical dynamic comparison ofbiodegradable pins and titaniumscrews for operative stabilization ofdisplaced radial head fractures | Proc Inst Mech Eng H | Germany | A |
| 2019 | 31703961 | Biomechanical comparison of bone-screw–fasteners versus traditionallocked screws in plating female geriatric bone | Injury | USA | C |
| 2019 | 31706308 | Novel implant-free loop Tenodesis vs.simple Tenotomy of the long bicepstendon - a biomechanical investigation | BMC Musculoskelet Disord | Germany | B |
| 2019 | 31707056 | Fifth metacarpal instability and its effect on hamatometacarpal arthritis patterns – a cadaver study | Hand Surg Rehabil | Israel | C |
| 2019 | 31727379 | Kinematics of Thumb Ulnar Collateral LigamentRepair With Suture Tape Augmentation | J Hand Surg Am | USA | C |
| 2019 | 31728187 | Short stems have lower load at failure than double-wedged stems in a cadaveric cementless fracture model | Bone Joint Res | Germany | A |
| 2019 | 31733980 | The Effect of Surgical Treatments forTrapeziometacarpal Osteoarthritis onWrist Biomechanics: A Cadaver Study | J Hand Surg Am | UK | A |
| 2019 | 31735063 | Differential Regional Stiffening of Sclera by Collagen Cross-linking | Curr Eye Res | USA | B |
| 2019 | 31737220 | Original ArticleBiomechanical analysis of cervical range of motion and facet contact force after a novel artificial cervical disc replacement | Am J Transl Res | China | C |
| 2019 | 31740014 | Validation of a Custom Spine Biomechanics Simulator: A Case for Standardi zation | J Biomech | USA | C |
| 2019 | 31740983 | Selective bundle tensioning in double-bundle MPFL reconstruction to improve restoration of dynamic patellofemoral contact pressure | Knee Surg Sports Traumatol Arthrosc | Germany | B |
| 2019 | 31746476 | The Utility of Electrocautery for Suture Passage Through Bone: A Biomechanical Study | J Orthop Res | USA | C |
| 2019 | 31763343 | Reconstruction of the Medial UlnarCollateral Ligament of the ElbowBiomechanical Comparison of a Novel AnatomicTechnique to the Docking Technique | Orthop J Sports Med | USA | B |
| 2019 | 31765242 | Engagement of the Secondary Ligamentousand Meniscal Restraints Relativeto the Anterior Cruciate LigamentPredicts Anterior Knee Laxity | Am J Sports Med | USA | A |
| 2019 | 31767285 | Biomechanical properties in motion of lumbar spines with degenerative scoliosis | J Biomech | The Netherlands | B |
| 2019 | 31774856 | Conventional rotator cuff versus all-sutureanchors—A biomechanical study focusing onthe insertion angle in an unlimited cyclicmodel | PLoS One | Germany | A |
| 2019 | 31779645 | Instability of the proximal radioulnar jointin Monteggia fractures—an experimentalstudy | J Orthop Surg Res | Germany | A |
| 2019 | 31779665 | Biomechanical comparison of subscapularispeel and lesser tuberosity osteotomy fordouble-row subscapularis repair techniquein a cadaveric arthroplasty model | J Orthop Surg Res | USA | A |

| | | | | | |
|---|---|---|---|---|---|
| 2019 | 31786511 | The measured mechanical properties of osteoporotic trabecular bone decline with the increment of deformation volume during micro-indentation | J Mech Behav Biomed Mater | Hong Kong | C |
| 2019 | 31791721 | Isometric placement of the augmentation braid is not attained reliably in contemporary ACL suture repair | Knee | UK | A |
| 2019 | 31791890 | Biomechanical Effect of Superior CapsuleReconstruction Using a 3-mm and 6-mm ThickAcellular Dermal Allograft in a DynamicShoulder Model | Arthroscopy | Germany | B |
| 2019 | 31803788 | Biomechanical Comparison of the Long Headof the Biceps Tendon Versus Conjoint TendonTransfer in a Bone Loss ShoulderInstability Model | Orthop J Sports Med | USA | B |
| 2019 | 31805924 | Influence of Glenosphere and baseplateparameters on Glenoid bone strains inreverse shoulder Arthroplasty | BMC Musculoskelet Disord | Germany | B |
| 2019 | 31810381 | The Perimensical Capsule: Potential Supporting Structure Surrounding Meniscus | Cartilage | Korea | C |
| 2019 | 31813563 | Biomechanical Contribution of the Alar Ligaments to Upper Cervical Stabili ty | J Biomech | USA | A |
| 2019 | 31813701 | Anterior cruciate ligament repair versus reconstruction: Akinematic analysis | Knee | USA | B |
| 2019 | 31819848 | A Biomechanical Evaluation of aNext-Generation Integrated and ModularACDF Device Possessing Full-Plate,Half-Plate, and No-Profile Fixation Iterations | Global Spine J | USA | B |
| 2019 | 31830593 | Discovery of a New Ligament of the Lumbar Spine: The MidlineInterlaminar Ligament | Spine J | USA | C |
| 2019 | 31859037 | Acellular dermal matrix augmentation significantlyincreases ultimate load to failure of pectoralismajor tendon repair: a biomechanical study | J Shoulder Elbow Surg | USA | B |
| 2019 | 31862009 | Architecture of the Short External Rotator Muscles of the Hip | BMC Musculoskelet Disord | USA | B |
| 2019 | 31862293 | Biomechanics Following Isolated Posterolateral Corner Reconstruction Comparinga Fibular-Based Docking Technique with a Tibia and Fibular-Based AnatomicTechnique Show Either Technique is Acceptal | Arthroscopy | USA | C |
| 2019 | 31862605 | Biomechanical Analysis of the Humeral Head Coverage, Glenoid Inclination and Acromio-glenoidal Height as isolated Components of the Critical Shoulder Angle in a Dynamic Cadaveric Shoulder Model | Clin Biomech (Bristol, Avon) | Switzerland | C |
| 2019 | 31864824 | The Relationship Between the Tensile and the Torsional Properties of the Native Scapholunate Ligament and Carpal Kinematics | J Hand Surg Am | USA | C |
| 2019 | 31864842 | Biomechanical Analysis of the Novel Syndesmotic Plate Compared WithTraditional Screw and Suture Button Fixation | J Foot Ankle Surg | China | A |
| 2019 | 31870565 | A Biomechanical Evaluation of the DRUJ AfterDistal Oblique Bundle Reconstruction | J Hand Surg Am | USA | C |
| 2019 | 31875233 | The tibial cut infuences the patellofemoral knee kinematics and pressure distribution in total knee arthroplasty with constitutional varus alignment | Knee Surg Sports Traumatol Arthrosc | Germany | A |
| 2019 | 31879494 | Biomechanical properties of an intramedullary suture anchor fixation compared totension band wiring in osteoporotic olecranon fractures- A cadaveric study | J Orthop | USA | C |
| 2019 | 31881914 | Tape suture for stabilization of incompleteposterior pelvic ringfractures—biomechanical analysis of a newminimally invasive treatment forincomplete lateral compression pelvic ringfractures | J Orthop Surg Res | Germany | A |
| 2019 | 31890409 | Biomechanical Analysis of Stand-alone LateralLumbar Interbody Fusion for Lumbar AdjacentSegment Disease | Cureus | USA | C |
| 2019 | 31903404 | Contributions of the Capsule and Labrumto Hip Mechanics in the Context ofHip Microinstability | Orthop J Sports Med | USA | B |
| 2019 | 31908934 | A novel calcium phosphate–based nanocomposite for the augmentation ofcement-injectable cannulated pedicle screws fixation: A cadaver andbiomechanical study | J Orthop Translat | China | A |
| 2019 | 31930066 | Effect of different fusion types on kinematics of midfoot lateral column: a comparative biomechanical study | Ann Transl Med | China | B |
| 2019 | 31946939 | Statistical shape-kinematics models of the skeletal joints: Application tothe shoulder complex | Annu Int Conf IEEE Eng Med Biol Soc | South Africa | A |
| 2019 | 32010728 | Variation in ACL and MCL Strain Before Initial Contact Is Dependent on Injury Risk Level During Simulated Landings | Orthop J Sports Med | USA | B |
| 2019 | 32030212 | Biomechanical comparison of monocortical and bicortical plate fixation for rib fractures in a cadaveric model using a locking plate system | J Thorac Dis | Singapore | B |
| 2019 | 32039176 | Age-Related Trends in the Trabecular Micro-Architecture of the Medial Clavicle: Is It of Use in Forensic Science? | Front Bioeng Biotechnol | UK | A |
| 2019 | 32257610 | Distal Metaphyseal Osteotomy Allows for Greater UlnarShortening Compared to Diaphyseal Osteotomy for UlnarImpaction Syndrome: A Biomechanical Study | J Wrist Surg | USA | B |
| 2019 | 32266351 | The Biomechanical Effects of Limited LateralRetinacular and Capsular Release on Lateral PatellarTranslation at Various Flexion Angles inCadaveric Specimens | Arthrosc Sports Med Rehabil | USA | B |
| 2019 | 32788931 | An in-vitro biomechanical assessmentof humeral head migration followingirreparable rotator cuff tear andsubacromial balloon reconstruction | Shoulder Elbow | Canada | A |
| 2019 | 34394742 | Effect of incremental increase inradial neck height on coronoidand capitellar contact pressures | Shoulder Elbow | USA | A |
| 2019 | 34659473 | Distal biceps tendon repairvia new knotless endobuttonfixation: A biomechanical study | Shoulder Elbow | France | B |
| 2019 | 35097339 | Biomechanical Comparison of theInfluences of 2 Proximal MetatarsalOsteotomies on First Ray ArticularContact Characteristics | Foot Ankle Orthop | USA | C |
| 2019 | 35097356 | Biomechanical Comparison of FirstTarsometatarsal Arthrodesis ConstructsOver Prolonged Cyclic Testing | Foot Ankle Orthop | USA | B |
| 2020 | 30526465 | Changes in Scaphoid and Lunate Position and Loading at Two Wrist Pushup Positions | Curr Rheumatol Rev | USA | C |
| 2020 | 31131859 | Kinematics Following 3-Screw Integrated Interbody Spacers in the Lumbar Spine | Oper Neurosurg (Hagerstown) | USA | B |
| 2020 | 31436615 | Acetabulum Cup Stability in an Early Weight-Bearing Cadaveric Model of Geriatric Posterior Wall Fractures | J Orthop Trauma | USA | C |
| 2020 | 31489460 | Varus alignment increases medial meniscus extrusion and peak contact pressure: a biomechanical study | Knee Surg Sports Traumatol Arthrosc | Germany | B |
| 2020 | 31593056 | Mechanisms of Failure Following Simulated Repetitive Lifting: A Clinically Relevant Biomechanical Cadaveric Study | Spine (Phila Pa 1976) | Australia | A |
| 2020 | 31669612 | Development of a novel in vitro cadaveric model for analysis of biomechanics and surgical treatment of Bertolotti syndrome | Spine J | USA | A |
| 2020 | 31692085 | Articular cartilage degradation and aberrant subchondral bone remodeling in patients with osteoarthritis and osteoporosis | J Bone Miner Res | China | A |
| 2020 | 31768546 | Restabilization of the Occipitocervical Junction After a Complete Unilateral Condylectomy: A Biomechanical Comparison of Unilateral and Bilateral Fixation Techniques | Oper Neurosurg (Hagerstown) | USA | A |
| 2020 | 31770344 | Superior-segment bilateral facet violation in lumbar transpedicular fixation, Part III: A biomechanical study of severe violation | Spine (Phila Pa 1976) | China | A |
| 2020 | 31791889 | Superior Capsule Reconstruction With Subacromial Allograft Spacer: Biomechanical Cadaveric Study of Subacromial Contact Pressure and Superior Humeral Head Translation | Arthroscopy | USA | C |
| 2020 | 31815856 | Biomechanical Evaluation of Pediatric Anterior Cruciate Ligament (ACL) Reconstruction Techniques With and Without the Anterolateral Ligament (ALL) | J Pediatr Orthop | USA | C |
| 2020 | 31864562 | Reconstruction of the Acromioclavicular Ligament Complex Using Dermal Allograft: A Biomechanical Analysis | Arthroscopy | USA | B |
| 2020 | 31894884 | Biometry of Thenar Muscle Origins on the Flexor Retinaculum | Clin Anat | USA | C |
| 2020 | 31902546 | The Effect of Glenohumeral Fixation Angle on Deltoid Function During Superior Capsule Reconstruction: A Biomechanical Investigation | Arthroscopy | USA | B |
| 2020 | 31902670 | Development and validation of a robust patellar reference coordinate system for biomechanical and clinical studies | Knee | Belgium | C |
| 2020 | 31904607 | Metacarpal Position and Lunate Facet Screw Fixation in Dorsal Wrist-Spanning Bridge Plates for Intra-Articular Distal Radial Fracture | J Bone Joint Surg Am | USA | A |
| 2020 | 31914474 | Impact of Medial Meniscotibial Ligament Disruption Compared to Peripheral Medial Meniscal Tear on Knee Biomechanics | J Knee Surg | USA | A |
| 2020 | 31921936 | Biomechanical Evaluation of Proximal Hamstring Repair All-Suture Anchor Versus Titanium Suture Anchor | Orthop J Sports Med | USA | B |
| 2020 | 31925759 | Biomechanical cadaver study of proximal fixation in a minimally invasive bipolar construct | Spine Deform | France | A |
| 2020 | 31926271 | Calcium Phosphate Bone Void Filler Increases Threaded Suture Anchor Pullout Strength: A Biomechanical Study | Arthroscopy | USA | C |
| 2020 | 31930414 | Open Biceps Tenodesis: A Biomechanical Comparison of 6 Fixation Techniques | Orthopedics | USA | B |
| 2020 | 31935225 | The strain distribution in the lumbar anterior longitudinal ligament is affected by the loading condition and bony features: An in vitro full-field analysis | PLoS One | Italy | A |
| 2020 | 31941350 | Extensor Tendon Transfers for Treatment of Foot Drop in Charcot-Marie-Tooth Disease: A Biomechanical Evaluation | Foot Ankle Int | USA | C |
| 2020 | 31948331 | Intramedullary screw fixation for metacarpal shaft fractures: a biomechanical human cadaver study | J Hand Surg Eur Vol | Switzerland | A |
| 2020 | 31952043 | Biomechanical evaluation of two alternative techniques to the Goel-Harms technique for atlantoaxial fixation: C1 lateral mass–C2 bicortical translaminar screw fixation and C1 lateral mass–C2/3 transarticul | J Neurosurg Spine | China | C |
| 2020 | 31954599 | Biomechanical Analysis of Midfoot Instability After Bifurcate Ligament Injury and Ankle Brace Application: A Cadaveric Study | J Foot Ankle Surg | Japan | A |
| 2020 | 31954610 | Muscular architecture of the popliteus muscle and the basic science implications | Knee | USA | B |
| 2020 | 31954807 | Biomechanical response to distraction of hip capsular reconstruction with human acellular dermal patch graft | Arthroscopy | USA | A |
| 2020 | 31960897 | An Approach to Robotic Testing of the Wrist Using Three-Dimensional Imaging and a Hybrid Testing Methodology | J Biomech Eng | USA | C |

| | | | | | |
|---|---|---|---|---|---|
| 2020 | 31969048 | An anatomical and biomechanical assessment of the interosseous membrane of the cadaveric forearm | J Hand Surg Eur Vol | Singapore | C |
| 2020 | 31972745 | Superior-segment Bilateral Facet Violation in Lumbar Transpedicular Fixation, Part I | Spine (Phila Pa 1976) | China | B |
| 2020 | 31973991 | Biomechanical Evaluation of Humerus Fracture after Subpectoral Biceps Tenodesis with Interference Screw Versus Unicortical Button | Arthroscopy | USA | C |
| 2020 | 31977668 | A Biomechanical Study of Tuberosity-Based Locked Plate Fixation Compared with Standard Proximal Humeral Locking Plate Fixation for 3-Part Proximal Humeral Fractures | J Orthop Trauma | USA | C |
| 2020 | 31980202 | Anatomic, All-arthroscopic Reconstruction of Posterolateral Corner of the Knee: A Cadaveric Biomechanical Study | Arthroscopy | China | A |
| 2020 | 31980844 | ACL reconstruction combined with lateral monoloop tenodesis can restore intact knee laxity | Knee Surg Sports Traumatol Arthrosc | UK | A |
| 2020 | 31989597 | High-resolution imaging of the human incudostapedial joint using synchrotron-radiation phase-contrast imaging | J Microsc | Canada | A |
| 2020 | 31989687 | Off-axis loads cause failure of the distal radius at lower magnitudes than axial loads: A side-to-side experimental study | J Orthop Res | Canada | A |
| 2020 | 32008806 | New insights on the proximal femur biomechanics using Digital Image Correlation | J Biomech | Israel | C |
| 2020 | 32010731 | Biomechanical Comparison of Anterograde and Retrograde Lesser Trochanter Avulsion Repair | Orthop J Sports Med | USA | B |
| 2020 | 32014412 | Biomechanical evaluation of PCL reconstruction with suture augmentation | Knee | USA | B |
| 2020 | 32023127 | Biomechanical impact testing of synthetic versus human cadaveric tibias for predicting injury risk during pedestrian-vehicle collisions | Traffic Inj Prev | Canada | C |
| 2020 | 32024608 | A novel approach for optimal graft positioning and tensioning in anterior cruciate ligament reconstructive surgery based on the finite element modeling technique | Knee | The Netherlands | B |
| 2020 | 32024609 | Effect of bone density and cement morphology on biomechanical stability of tibial unicompartmental knee arthroplasty | Knee | Germany | A |
| 2020 | 32026440 | Increasing loads and diminishing returns: a biomechanical study of direct vertebral rotation | Spine Deform | USA | A |
| 2020 | 32032183 | Supplemental Fixation of Supracondylar Distal Femur Fractures: A Biomechanical Comparison of Dual-Plate and Plate-Nail Constructs | J Orthop Trauma | USA | C |
| 2020 | 32033807 | Biomechanical stability of sacroiliac screw osteosynthesis with and without cement augmentation | Injury | Germany | A |
| 2020 | 32035171 | Vertical and Rotational Stiffness of Coracoclavicular Ligament Reconstruction: A Biomechanical Study of 3 Different Techniques | Arthroscopy | USA | A |
| 2020 | 32044251 | Effect of ulnar angulation and soft tissue sectioning on radial head stability in anterior Monteggia injuries: an in vitro biomechanical study | J Shoulder Elbow Surg | Canada | B |
| 2020 | 32047832 | Anatomic Knot Suture Anchor Versus Knotless Suture Anchor Technique for Anterior Talofibular Ligament Repair A Biomechanical Comparison | Orthop J Sports Med | China | A |
| 2020 | 32057982 | Biomechanical Evaluation of Knotless and Knotted All-Suture Anchor Repair Constructs in Four Bankart Repair Configurations | Arthroscopy | USA | C |
| 2020 | 32060592 | The novel arthroscopic subscapular quadriceps tendon–bone sling procedure provides increased stability in shoulder cadavers with severe glenoid bone loss | Knee Surg Sports Traumatol Arthrosc | Norway | A |
| 2020 | 32063347 | Tendon With Z-Lengthening Technique and Construct Assessment: A Biomechanical Study for Use in Tendon Reconstructions | J Hand Surg Am | USA | C |
| 2020 | 32081633 | Synthetic coracoclavicular ligament vs. coracoclavicular suspensory construct for treatment of acromioclavicular dislocation: a biomechanical study | J Shoulder Elbow Surg | USA | B |
| 2020 | 32081635 | Effect of biceps rerouting technique to restore glenohumeral joint stability for large irreparable rotator cuff tears: a cadaveric biomechanical study | J Shoulder Elbow Surg | Korea | A |
| 2020 | 32085779 | Syndesmotic malreduction may decrease fixation stability: a biomechanical study | J Orthop Surg Res | China | A |
| 2020 | 32088987 | Median and Digital Nerve In Situ Tension in the Hand | Hand (N Y) | USA | A |
| 2020 | 32100567 | How Close Are the Volar Wrist Ligaments to the Distal Edge of the Pronator Quadratus? An Anatomical Study | Hand (N Y) | USA | B |
| 2020 | 32106851 | Investigating the biomechanical function of the plate-type external fixator in the treatment of tibial fractures: a biomechanical study | BMC Musculoskelet Disord | China | A |
| 2020 | 32108254 | Local osteo-enhancement of osteoporotic vertebra with a triphasic bone implant material increases strength—a biomechanical study | Arch Orthop Trauma Surg | Austria | C |
| 2020 | 32108521 | Optimal Position of the Suture Button Suspensionplasty (TightRope) for Thumb Basal Joint Arthritis | Hand (N Y) | USA | A |
| 2020 | 32130480 | Comparison of various tendon repair techniques in extansor zone 3 injuries: an experimental biomechanical cadaver study | Arch Orthop Trauma Surg | Turkey | A |
| 2020 | 32131794 | Ankle joint pressure change in varus malalignment of the tibia | BMC Musculoskelet Disord | China | A |
| 2020 | 32139286 | Biomechanical comparison of tension band wiring and plate fixation with locking screws in transverse olecranon fractures | J Shoulder Elbow Surg | Norway | A |
| 2020 | 32147337 | Cortical suture button fixation vs. bicortical screw fixation in the Latarjet procedure: a biomechanical comparison | J Shoulder Elbow Surg | USA | B |
| 2020 | 32156460 | A comparison of two designs of postoperative shoe for hallux valgus surgery: A biomechanical study in a cadaveric model | Foot Ankle Surg | Spain | A |
| 2020 | 32156663 | A novel mechanical parameter to quantify the microarchitecture effect on apparent modulus of trabecular bone: A computational analysis of ineffective bone mass | Bone | Hong Kong | C |
| 2020 | 32173084 | Is intramedullary screw fixation biomechanically superior to locking plate fixation and/or tension band wiring in transverse olecranon fractures? | Injury | USA | C |
| 2020 | 32173475 | Left Common Carotid Artery Biomechanical Properties in Individuals over 80 years: Women Have Stiffer Vessels | Ann Vasc Surg | Brazil | A |
| 2020 | 32174404 | Failure mechanisms of pedicle screws and cortical screws fixation under large displacement: A biomechanical and microstructural study based on a clinical case scenario | J Mech Behav Biomed Mater | Hong Kong | B |
| 2020 | 32178972 | Anterolateral ligament reconstruction as an augmented procedure for double-bundle anterior cruciate ligament reconstruction restores rotational stability: Quantitative evaluation of the pivot shift test using a[illegible] | Knee | Japan | A |
| 2020 | 32185569 | Automated Generation of Three-Dimensional Complex Muscle Geometries for Use in Personalised Musculoskeletal Models | Ann Biomed Eng | UK | C |
| 2020 | 32192993 | Biomechanical analysis of two insertion sites for the fixation of the sacroiliac joint via an oblique lateral approach | Clin Biomech (Bristol, Avon) | Canada | A |
| 2020 | 32195827 | Tuberosity Repair in Reverse Total Shoulder Arthroplasty for Fracture Using a Stem-based Double-row Repair: A Cadaveric Biomechanical Study | J Am Acad Orthop Surg | USA | C |
| 2020 | 32197036 | Mechanical Effects of Bone Substitute and Far-Cortical Locking Techniques in 2-Part Proximal Humerus Fracture Reconstruction: A Cadaveric Study | J Orthop Trauma | USA | C |
| 2020 | 32197808 | The moving valgus stress test produces more ulnar collateral ligament change in length during extension than during flexion: a biomechanical study | J Shoulder Elbow Surg | USA | B |
| 2020 | 32197829 | Biomechanical comparison of a 3.5 mm anterior locking plate to cannulated screws with anterior tension band wiring in comminuted patellar fractures | Injury | Germany | A |
| 2020 | 32205691 | Development and Validation of Finite Element Analysis Model (FEM) of Craniovertebral Junction | Spine (Phila Pa 1976) | India | A |
| 2020 | 32206353 | A Biomechanical Comparison of Two Techniques of Latarjet Procedure in Cadaveric Shoulders | Adv Orthop | UK | C |
| 2020 | 32220536 | Distalising tibial tubercle osteotomy decreases patellar tendon force — A treatment rationale for recalcitrant patellar tendinopathy | Knee | Australia | A |
| 2020 | 32236747 | Effects of a novel medial meniscus implant on the knee compartments: imaging and biomechanical aspects | Biomech Model Mechanobiol | Israel | A |
| 2020 | 32249406 | Biomechanics of prophylactic tethering for proximal junctional kyphosis: effects of cyclic loading on tether strength and failure properties | Spine Deform | USA | C |
| 2020 | 32251683 | Anterolateral Ligament Reconstruction and Modified Lemaire Lateral Extra-Articular Tenodesis Improve Knee Stability After Anterior Cruciate Ligament Reconstruction: A Biomechanical Study | Arthroscopy | Switzerland | B |
| 2020 | 32253152 | Dimensional Changes of the Tarsal Tunnel During Foot and Ankle Positions: Anatomical Study | J Foot Ankle Surg | Spain | A |
| 2020 | 32259645 | Using Interconnected Knotless Anchor for Suprapectoral Biceps Tenodesis Could Offer Improved Biomechanical Properties in a Cadaveric Model | Arthroscopy | Taiwan | B |
| 2020 | 32268403 | In Vitro Kinematic Analysis of Single Axis Radius Posterior-Substituting Total Knee Arthroplasty | J Knee Surg | USA | C |
| 2020 | 32276257 | Biomechanical analysis of occipitocervical stabilization techniques: emphasis on integrity of osseous structures at the occipital implantation sites | J Neurosurg Spine | USA | C |
| 2020 | 32276814 | Biomechanical Analysis of Zone 2 Flexor Tendon Repair With a Coupler Device Versus Locking Cruciate Core Suture | J Hand Surg Am | USA | C |
| 2020 | 32277264 | The superficial medial collateral ligament is the major restraint to anteromedial instability of the knee | Knee Surg Sports Traumatol Arthrosc | Austria | A |
| 2020 | 32278344 | Cement augmentation of an angular stable plate osteosynthesis for supracondylar femoral fractures - biomechanical investigation of a new fixation device | BMC Musculoskelet Disord | Germany | A |
| 2020 | 32278525 | Ultrasound echogenicity is associated with fatigue-induced failure in a cadaveric Achilles tendon model | J Biomech | USA | C |
| 2020 | 32279845 | Human lumbar spinal column injury criteria from vertical loading at the base: Applications to military environments | J Mech Behav Biomed Mater | USA | A |
| 2020 | 32280069 | Screw fixation for syndesmotic injury is stronger and provides more contact area of the joint surface than TightRope®: A biomechanical study | Technol Health Care | Germany | B |
| 2020 | 32286490 | Digitalization of the IOM: A comprehensive cadaveric study for obtaining three-dimensional models and morphological properties of the forearm’s interosseous membrane | Sci Rep | Switzerland | A |

| | | | | | |
|---|---|---|---|---|---|
| 2020 | 32306090 | Contact mechanics after mattress suture repair of medial meniscus vertical longitudinal tear: an in vitro study | Arch Orthop Trauma Surg | China | A |
| 2020 | 32306132 | The tibial cut in total knee arthroplasty influences the varus alignment, the femoral roll-back and the tibiofemoral rotation in patients with constitutional varus | Knee Surg Sports Traumatol Arthrosc | Germany | B |
| 2020 | 32309640 | Pullout force of minimally invasive surgical and open pedicle screws—a biomechanical cadaveric study | J Spine Surg | Australia | A |
| 2020 | 32309757 | Effects of Lumbosacral Arthrodesis on the Biomechanics of the Sacroiliac Joint | JB JS Open Access | USA | C |
| 2020 | 32310106 | Effect of sitting posture on pelvic injury risk under vertical loading | J Mech Behav Biomed Mater | Australia | A |
| 2020 | 32314105 | An MRI-compatible varus-valgus loading device for whole-knee joint functionality assessment based on compartmental compression: a proof-of-concept study | MAGMA | Germany | A |
| 2020 | 32321693 | The biomechanical impact of hip movement on iliofemoral venous anatomy and stenting for deep venous thrombosis | J Vasc Surg Venous Lymphat Disord | UK | C |
| 2020 | 32327339 | A Biomechanical Comparison of Modified Radioscapholunate Fusion Constructs for Radiocarpal Arthritis | J Hand Surg Am | USA | C |
| 2020 | 32333606 | Assessing the Biofidelity of In Vitro Biomechanical Testingof the Human Cervical Spine | J Orthop Res | USA | C |
| 2020 | 32335469 | Probe versus drill: A biomechanical evaluation of two different pedicle preparation techniques for pedicle screw fixation in human cadaveric osteoporotic spine | Clin Biomech (Bristol, Avon) | Germany | A |
| 2020 | 32338311 | Single-bundle MCL reconstruction with anatomic single-bundle ACL reconstruction does not restore knee kinematics | Knee Surg Sports Traumatol Arthrosc | USA | A |
| 2020 | 32347346 | Isolated MPTL reconstruction fails to restore lateral patellar stability when compared to MPFL reconstruction | Knee Surg Sports Traumatol Arthrosc | Brazil | A |
| 2020 | 32348038 | Comparative biomechanical testing of customized three-dimensional printing acetabular-wing plates for complex acetabular fractures | Adv Clin Exp Med | China | A |
| 2020 | 32353619 | Suture Tape Reduces Quadriceps Tendon Repair Gap Formation Compared to High Strength Suture: A Cadaveric Biomechanical Analysis | Arthroscopy | Canada | A |
| 2020 | 32355618 | Variations Among Human Lumbar Spine Segments and Their Relationships to In Vitro Biomechanics: A Retrospective Analysis of 281 Motion Segments From 85 Cadaveric Spines | Int J Spine Surg | USA | C |
| 2020 | 32360177 | A new intramedullary fixation method for distal biceps tendon ruptures: a biomechanical study | J Shoulder Elbow Surg | Belgium | A |
| 2020 | 32361929 | The quadriceps insertion of the medial patellofemoral complex demonstrates the greatest anisometry through flexion | Knee Surg Sports Traumatol Arthrosc | USA | B |
| 2020 | 32371284 | Analysis of the mechanical response of damaged human cervical spine ligaments | Clin Biomech (Bristol, Avon) | Slovenia | A |
| 2020 | 32372281 | The Popliteus Bypass provides superior biomechanical properties compared to the Larson technique in the reconstruction of combined posterolateral corner and posterior cruciate ligament injury | Knee Surg Sports Traumatol Arthrosc | Germany | A |
| 2020 | 32386522 | Strain pattern of each ligamentous band of the superficial deltoid ligament: a cadaver study | BMC Musculoskelet Disord | Spain | A |
| 2020 | 32395569 | Raman microspectroscopy demonstrates reduced mineralization of subchondral bone marrow lesions in knee osteoarthritis patients | Bone Rep | Australia | A |
| 2020 | 32396386 | Assessment of the Degree of Osteoarthritis in Aging Male and Female Femoral Condyles: A Cadaveric Study | Cartilage | USA | A |
| 2020 | 32417047 | Biomechanical comparison of biodegradable magnesium screws and titanium screws for operative stabilization of displaced capitellar fractures | J Shoulder Elbow Surg | Germany | B |
| 2020 | 32418057 | Anchor placement to glenoid rim during Bankart repair recreates contact area of anterior capsulolabral complex on glenoid better than onto articular surface | Eur J Orthop Surg Traumatol | USA | A |
| 2020 | 32422471 | Kinetic dysfunction of the wrist with chronic scapholunate dissociation. A cadaver study | Clin Biomech (Bristol, Avon) | Spain | C |
| 2020 | 32423548 | Asymmetric in-plane shear behavior of isolated cadaveric lumbar facet capsular ligaments: Implications for subject specific biomechanical models | J Biomech | USA | B |
| 2020 | 32424638 | Novel augmentation technique of percutaneous pedicle screw fixation using hydroxyapatite granules in the osteoporotic lumbar spine: a cadaveric biomechanical analysis | Eur Spine J | Japan | A |
| 2020 | 32434732 | Internal Bracing Augmentation for Scapholunate Interosseous Ligament Repair: A Cadaveric Biomechanical Study | J Hand Surg Am | Korea | C |
| 2020 | 32437222 | The strengths of one-, two-, and three-weave Pulvertaft tendon repairs | J Hand Surg Eur Vol | Singapore | C |
| 2020 | 32438009 | The role of transverse connectors in C1 -C2 fixation for atlantoaxial instability: is it necessary? - A biomechanical study | World Neurosurg | China | A |
| 2020 | 32438029 | Biomechanical Evaluation of an All-Inside Posterior Medial Meniscal Root Repair Technique via Suture Fixation to the Posterior Cruciate Ligament | Arthroscopy | USA | B |
| 2020 | 32442519 | OCCLUSION OF THE LUMBAR SPINE CANAL DURING HIGH-RATE AXIAL COMPRESSION | Spine J | Australia | A |
| 2020 | 32452267 | Headless Compression Screw Fixation for Proximal Phalanx Fractures: A Biomechanical Study | Hand (N Y) | USA | A |
| 2020 | 32453667 | Biomechanical Functional Elbow Restoration of Acute Ulnar Collateral Ligament Tears | Am J Sports Med | Germany | B |
| 2020 | 32461022 | A Cadaveric Study of Cam Type Femoroacetabular Impingement: Biomechanical Comparison of Contact Pressures Between Cam Deformity, Partial Femoral Osteoplasty, and Complete Femoral Osteoplasty | Arthroscopy | USA | B |
| 2020 | 32462522 | Four-strand hamstring graft is stiffer than a tripled semitendinosus graft in anterior cruciate ligament reconstruction: a cadaveric study | J Exp Orthop | The Netherlands | A |
| 2020 | 32462810 | Biomechanical Study on the Stress Distribution of the Knee Joint After Tibial Fracture Malunion with Residual Varus–Valgus Deformity | Orthop Surg | China | A |
| 2020 | 32474224 | Biomechanical contribution of spinal structures to stability of the lumbar spine – Novel biomechanical insights. | Spine J | Switzerland | A |
| 2020 | 32474717 | Biomechanical model of distal articular humeral fractures—influence of bone density on the fracture threshold | Int Orthop | France | C |
| 2020 | 32490406 | Biomechanical assessment of lateral ulnar collateral ligament repair and reconstruction with or without internal brace augmentation | JSES Int | USA | A |
| 2020 | 32495639 | Peritalar Kinematics With Combined Deltoid-Spring Ligament Reconstruction in Simulated Advanced Adult Acquired Flatfoot Deformity | Foot Ankle Int | USA | C |
| 2020 | 32499069 | Osteosynthesis of Phalangeal Fractures: Biomechanical Comparison of Kirschner Wires, Plates, and Compression Screws | J Hand Surg Am | Germany | C |
| 2020 | 32501222 | 3D full-field strain in bone-implant and bone-tooth constructs and their morphological influential factors | J Mech Behav Biomed Mater | USA | B |
| 2020 | 32504714 | Comparison of Suction Seal and Contact Pressures Between 270 Degree Labral Reconstruction, Labral Repair, and the Intact Labrum | Arthroscopy | USA | B |
| 2020 | 32504897 | Biomechanical comparison of anterior cruciate ligament repair with internal brace augmentation versus anterior cruciate ligament repair without augmentation | Clin Biomech (Bristol, Avon) | USA | B |
| 2020 | 32506962 | Biomechanical Analysis of Conventional Partially Threaded Screws Versus Headless Compression Screws in Proximal Fifth Metatarsal (Jones) Fracture Fixation | Foot Ankle Spec | USA | C |
| 2020 | 32517515 | Do "Anatomic" Distal Ulna Plating Systems Fit the Distal Ulna Without Causing Soft Tissue Impingement? | Hand (N Y) | UK | A |
| 2020 | 32525582 | Dual contrast in computed tomography allows earlier characterization of articular cartilage over single contrast | J Orthop Res | Finland | A |
| 2020 | 32543737 | Orientation anisotropy of quantitative MRI parameters in degenerated human articular cartilage | J Orthop Res | Finland | A |
| 2020 | 32545997 | Biomechanical Effect on Joint Stability of Including Deltoid Ligament Repair in an Ankle Fracture Soft Tissue Injury Model With Deltoid and Syndesmotic Disruption | Foot Ankle Int | USA | A |
| 2020 | 32551965 | Comparison of Patellofemoral Kinematics and Stability After Medial Patellofemoral Ligament and Medial Quadriceps Tendon–Femoral Ligament Reconstruction | Am J Sports Med | USA | B |
| 2020 | 32553424 | A cadaveric comparison of two methods for isolated talonavicular arthrodesis: Two-screws versus plate with integrated compression screw | Foot Ankle Surg | USA | C |
| 2020 | 32562881 | Biomechanical analysis of simultaneous distal and proximal radio-ulnar joint instability | Clin Biomech (Bristol, Avon) | Thailand | A |
| 2020 | 32565411 | Evaluation of anterior oblique ligament tension at the elbow joint angle—a cadaver study. | J Shoulder Elbow Surg | Japan | A |
| 2020 | 32574472 | Does Coronal Plane Malalignment of the Tibial Insert in Total Ankle Arthroplasty Alter Distal Foot Bone Mechanics? A Cadaveric Gait Study | Clin Orthop Relat Res | USA | B |
| 2020 | 32576207 | A novel protection liner to improve graft-tunnel interaction following anterior cruciate ligament reconstruction: a finite element analysis | J Orthop Surg Res | China | A |
| 2020 | 32585072 | Cortical and medullary morphology of the tibia | Anat Rec (Hoboken) | USA | B |
| 2020 | 32586389 | Does endoscopic sinus surgery alter the biomechanics of the orbit? | J Otolaryngol Head Neck Surg | Canada | A |
| 2020 | 32591261 | Biomechanical Comparison of Knotless All-Suture Anchors and Knotted All-Suture Anchors in Type II SLAP Lesions: A Cadaveric Study | Arthroscopy | USA | B |
| 2020 | 32600743 | The role of biceps loading and muscle activation on radial head stability in anterior Monteggia injuries: An in vitro biomechanical study | J Hand Ther | Canada | C |
| 2020 | 32605784 | A pilot biomechanical study comparing a novel, intramedullary Nail/Plate construct to standard Dual-Plate fixation of intra-articular C2.3 distal humerus fractures | Injury | USA | B |
| 2020 | 32619657 | Biodegradable Magnesium versus Polylactide Pins for Radial Head Fracture Stabilization: A Biomechanical Study | J Shoulder Elbow Surg | Germany | B |
| 2020 | 32621980 | The Influence of Reverse Arthroplasty Humeral Component Design Features on Scapular Spine Strain | J Shoulder Elbow Surg | Canada | B |
| 2020 | 32624266 | Sagittal split ramus osteotomy-related biomechanicalproperties | Br J Oral Maxillofac Surg | France | A |

| | | | | | |
|---|---|---|---|---|---|
| 2020 | 32632532 | Filtration Selection and Data Consilience: Distinguishing Signal from Artefact with Mechanical Impact Simulator Data | Ann Biomed Eng | USA | A |
| 2020 | 32638068 | The biomechanics of the supraspinatus-deficient shoulder treated with superior capsular reconstruction vs. reverse total shoulder arthroplasty—experimental study | Int Orthop | USA | C |
| 2020 | 32650692 | Fixation of distal tibia fracture through plating, nailing, and nailing with Poller screws: A comparative biomechanicalbased experimental and numerical investigation | Proc Inst Mech Eng H | Iran | A |
| 2020 | 32650813 | A comparison of EFECE systems with tension band wiring for patella fracture fixation in cadavers | J Orthop Surg Res | Turkey | A |
| 2020 | 32654121 | Anatomical relationship between the morphology of the styloid process of the ulna and the attachment of the radioulnar ligaments | J Anat | Japan | A |
| 2020 | 32669200 | Influence of implant design and parasagittal acromial morphology on acromial and scapular spine strain after reverse total shoulder arthroplasty: a cadaveric and computer-based biomechanical analysis | J Shoulder Elbow Surg | USA | A |
| 2020 | 32669201 | Biomechanical effects of superior capsular reconstruction in a rotator cuff–deficient shoulder: a cadaveric study | J Shoulder Elbow Surg | USA | B |
| 2020 | 32672067 | Comparison of External Torque to Axial Loading in Detecting 3-Dimensional Displacement of Syndesmotic Ankle Injuries | Foot Ankle Int | Germany | B |
| 2020 | 32676888 | An evaluation of the objectivity and reproducibility of shear wave elastography in estimating the post-mortem interval: a tissue biomechanical perspective | Int J Legal Med | Italy | A |
| 2020 | 32682448 | The T-pod is as stable as supraacetabular fixation using 1 or 2 Schanz screws in partially unstable pelvic fractures: a biomechanical study | Eur J Med Res | Germany | A |
| 2020 | 32685524 | Extension Mechanism of the Proximal Interphalangeal Joint of the Human Phalanx: A Cadaveric Biomechanical Study | Biomed Res Int | Korea | C |
| 2020 | 32690338 | Biomechanical Evaluation of Distal Radioulnar Joint Instability and Adams Procedure | J Hand Surg Am | USA | C |
| 2020 | 32691628 | Update on the Biomechanics of the Craniocervical Junction, Part II: Alar Ligament | Global Spine J | Germany | B |
| 2020 | 32692952 | Posterior Rotational and Translational Stability in Acromioclavicular Ligament Complex Reconstruction A Comparative Biomechanical Analysis in Cadaveric Specimens | Am J Sports Med | USA | B |
| 2020 | 32693617 | Linear Discriminant Analysis Successfully Predicts Knee Injury Outcome From Biomechanical Variables | Am J Sports Med | USA | C |
| 2020 | 32712527 | Biomechanical in vitro examination of a standardized low-volume tubular femoroplasty | Clin Biomech (Bristol, Avon) | Germany | C |
| 2020 | 32712685 | Function and strain of the anterolateral ligament part II: reconstruction | Knee Surg Sports Traumatol Arthrosc | Germany | A |
| 2020 | 32727593 | Biomechanical effects of stitches on the intra-articular mid-substance of quadruple hamstring-tendon grafts for anterior cruciate ligament reconstruction – a pilot comparative cadaveric study | Knee Surg Relat Res | France | B |
| 2020 | 32741565 | Mechanism and Patterns of Bone Loss in Patients with Anterior Shoulder Dislocation | J Shoulder Elbow Surg | Japan | A |
| 2020 | 32745705 | Downsizing effect of a modular radial head prosthesis on the lateral collateral ligament of the elbow: A cadaveric study | Clin Biomech (Bristol, Avon) | Japan | A |
| 2020 | 32753357 | Knee laxity in anterolateral complex injuries versus medial meniscus posterior horn injuries in anterior cruciate ligament injured knees: A cadaveric study | Orthop Traumatol Surg Res | Korea | B |
| 2020 | 32757034 | Improved femoral component rotation in total knee arthroplasty: an anatomical study with optimized gap balancing | Arch Orthop Trauma Surg | Germany | B |
| 2020 | 32762632 | The Hip Suction Seal, Part II The Effect of Rim Trimming, Chondrolabral Junction Separation, and Labral Repair/Refixation on Hip Distractive Stability | Am J Sports Med | USA | C |
| 2020 | 32762634 | The Hip Suction Seal, Part I The Role of Acetabular Labral Height on Hip Distractive Stability | Am J Sports Med | USA | C |
| 2020 | 32768654 | Influences of functional structures on the kinematic behavior of the cervical spine | Spine J | Germany | A |
| 2020 | 32775474 | Surgical Treatment of Meniscal Extrusion A Biomechanical Study on the Role of the Medial Meniscotibial Ligaments With Early Clinical Validation | Orthop J Sports Med | USA | B |
| 2020 | 32777399 | Comparison of Long Segmental Dorsal Stabilization with Complete Versus Restricted Pedicle Screw Cement Augmentation in Unstable Osteoporotic Midthoracic Vertebral Body Fractures: A Biomechanica | World Neurosurg | Germany | C |
| 2020 | 32778385 | Biomechanics of tensor fascia lata allograft for superior capsular reconstruction | J Shoulder Elbow Surg | USA | B |
| 2020 | 32781849 | Staple-Plate Plus Screw vs Screw Alone in Talonavicular Arthrodesis: A Cadaveric Biomechanical Study | Foot Ankle Int | USA | C |
| 2020 | 32787891 | The biomechanical effect of different posterior tibial slopes on the tibiofemoral joint after posterior-stabilized total knee arthroplasty | J Orthop Surg Res | China | A |
| 2020 | 32788041 | Comparing Internal Fixation Constructs for Scapular Spine Insufficiency Fractures following Reverse Shoulder Arthroplasty | J Shoulder Elbow Surg | Canada | B |
| 2020 | 32796462 | Pedicle Screw System May Not Control Severe Spinal Rotational Instability | Spine (Phila Pa 1976) | Japan | A |
| 2020 | 32800315 | Stability of internal versus external fixation in osteoporotic pelvic fractures –a biomechanical analysis | Injury | Germany | A |
| 2020 | 32807334 | The effect of coordinate system selection on wrist kinematics | J Biomech | Canada | C |
| 2020 | 32811693 | Suture Tape Augmentation for Scapholunate Ligament Repair: A Biomechanical Study | J Hand Surg Am | USA | C |
| 2020 | 32813568 | Medial Elbow Instability Resulting From Partial Tears of the Ulnar Collateral Ligament | Am J Sports Med | USA | B |
| 2020 | 32815840 | Biomechanical Stability of Volar Plate Only Versus Addition of Dorsal Ulnar Pin Plate: A Dorsal Ulnar Fragment, C-3–Type, Distal Radius, Cadaver Fracture Model | J Orthop Trauma | USA | B |
| 2020 | 32816417 | A Calcar Collar Is Protective Against Early Torsional/ Spiral Periprosthetic Femoral Fracture | J Bone Joint Surg Am | USA | C |
| 2020 | 32829037 | Conventional finite element models estimate the strength of metastatic human vertebrae despite alterations of the bone's tissue and structure | Bone | Switzerland | B |
| 2020 | 32829232 | Biomechanical effects of posterior pedicle screw-based instrumentation using titanium versus carbon fiber reinforced PEEK in an osteoporotic spine human cadaver model | Clin Biomech (Bristol, Avon) | Germany | A |
| 2020 | 32835387 | Biomechanical Assessment of a Novel Posterior Soft Tissue Repair Technique in Primary Total Hip Arthroplasty | Surg Technol Int | USA | C |
| 2020 | 32838808 | In vitro study of foot bone kinematics via a custom-made cadaveric gait simulator | J Orthop Surg Res | China | A |
| 2020 | 32858196 | Glenoid Baseplate Screw Fixation in Reverse Shoulder Arthroplasty: Does Locking Screw Position and Orientation Matter? | J Shoulder Elbow Surg | Canada | B |
| 2020 | 32862075 | Optimal configuration of a dual locking plate for femoral allograft or recycled autograft bone fixation: A finite element and biomechanical analysis | Clin Biomech (Bristol, Avon) | Thailand | A |
| 2020 | 32864384 | Differences in Failure Mode Between Simple and Mattress Suture Configuration in Arthroscopic Bankart Repairs A Cadaveric Study | Orthop J Sports Med | USA | B |
| 2020 | 32865631 | Rotational range of motion of elliptical and spherical heads in shoulder arthroplasty: a dynamic biomechanical evaluation | Arch Orthop Trauma Surg | USA | B |
| 2020 | 32867789 | Wrist movements induce torque and lever force in the scaphoid: an ex vivo study | J Orthop Surg Res | Austria | A |
| 2020 | 32867845 | How the clinical dosage of bone cement biomechanically affects adjacent vertebrae | J Orthop Surg Res | China | A |
| 2020 | 32871265 | Tensioning device increases biomechanical stability of tuberosity fixation technique with cerclage sutures in reverse shoulder arthroplasty for fracture | J Shoulder Elbow Surg | Germany | A |
| 2020 | 32875824 | Ankle Joint Pressure in Supination–External Rotation Injuries A Biomechanical Study in an Unrestrained Cadaver Model | Foot Ankle Spec | Switzerland | C |
| 2020 | 32875854 | Update on the Biomechanics of the Craniocervical Junction—Part I: Transverse Atlantal Ligament in the Elderly | Global Spine J | USA | B |
| 2020 | 32875868 | An In Vitro Biomechanical Evaluation of a Lateral Lumbar Interbody Fusion Device With Integrated Lateral Modular Plate Fixation | Global Spine J | USA | C |
| 2020 | 32875910 | Rescue Augmentation: Increased Stability in Augmentation After Initial Loosening of Pedicle Screws | Global Spine J | Germany | C |
| 2020 | 32881027 | THE KNEE PROSTHESIS CONSTRAINT DILEMMA: BIOMECHANICAL COMPARISON BETWEEN VARUS-VALGUS CONSTRAINED IMPLANTS AND ROTATING HINGE PROSTHESIS. A C. | J Orthop Res | Spain | A |
| 2020 | 32881582 | The Effect of Resection Size in the Treatment of Cam-Type Femoroacetabular Impingement in the Typical Patient With Hip Arthroscopy | Am J Sports Med | USA | B |
| 2020 | 32884007 | A cadaver-based biomechanical model of acetabulum reaming for surgical virtual reality training simulators | Sci Rep | Germany | A |
| 2020 | 32889558 | Partial meniscectomy does not affect the biomechanics of anterior cruciate ligament reconstructed knee with a lateral posterior meniscal root tear | Knee Surg Sports Traumatol Arthrosc | USA | A |
| 2020 | 32891838 | Biomechanical Analysis of an Interspinous Process Fixation Device with In Situ Shortening Capabilities: Does Spinous Process Compression Improve Segmental Stability | World Neurosurg | USA | C |
| 2020 | 32906084 | Effect of cervical contralateral lateral flexion on displacement and strain in the median nerve and flexor digitorum superficialis at the wrist during the ULNT1 – Cadaveric study | Musculoskelet Sci Pract | Spain | A |
| 2020 | 32927407 | Effect of cup medialization on primary stability of press-fit acetabular cups | Clin Biomech (Bristol, Avon) | Italy | A |
| 2020 | 32929557 | Stiffness and Strength Predictions From Finite Element Models of the Knee are Associated with Lower-Limb Fractures After Spinal Cord Injury | Ann Biomed Eng | Canada | A |
| 2020 | 32935574 | Biomechanical Stability of Primary and Revision Sacroiliac Joint Fusion Devices: A Cadaveric Study | Global Spine J | USA | B |
| 2020 | 32936405 | Influence of double rods and interbody cages on quasistatic range of motion of the spine after lumbopelvic instrumentation | Eur Spine J | France | C |
| 2020 | 32940897 | Occupant Restraint in Far-Side Impacts: Cadaveric and WorldSID Responses to a Far-Side Airbag | Ann Biomed Eng | USA | A |

| | | | | | |
|---|---|---|---|---|---|
| 2020 | 32949938 | Strain measurement of the deep layer of the supraspinatus tendon using fresh frozen cadaver: The influence of shoulder elevation | Clin Biomech (Bristol, Avon) | Japan | A |
| 2020 | 32957237 | Revision total knee arthroplasty using a novel 3D printed titanium augment: A biomechanical cadaveric study | J Mech Behav Biomed Mater | Canada | A |
| 2020 | 32957856 | The use of barbed sutures in the Pulvertaft weave: a biomechanical study | J Hand Surg Eur Vol | Singapore | C |
| 2020 | 32972397 | Biomechanical comparison between singlerow with triple-loaded suture anchor and suture-bridge double-row rotator cuff repair | BMC Musculoskelet Disord | China | A |
| 2020 | 32977915 | Vertebral stiffness measured via tomosynthesis-based digital volume correlation is strongly correlated with reference values from micro-CT-based DVC | Med Eng Phys | USA | A |
| 2020 | 32978021 | Biomechanical Study of Patellar Component Fixation with Varying Degrees of Bone Loss | J Arthroplasty | USA | C |
| 2020 | 32981339 | Biomechanical Comparison of Hook Plate vs Headless Compression Screw Fixation of Large Fifth Metatarsal Base Avulsion Fractures | Foot Ankle Int | USA | B |
| 2020 | 32986580 | Evaluation of Spinous Process Tethering at the Proximal End of Rigid Constructs: In Vitro Range of Motion and Intradiscal Pressure at Instrumented and Adjacent Levels | Int J Spine Surg | USA | C |
| 2020 | 32990809 | Radioulnar joint reconstruction: a novel surgical technique and biomechanical assessment for chronic instability | J Exp Orthop | Brazil | A |
| 2020 | 32992567 | Primary Stability of Revision Acetabular Reconstructions Using an Innovative Bone Graft Substitute: A Comparative Biomechanical Study on Cadaveric Pelvises | Materials (Basel) | Italy | A |
| 2020 | 33010434 | Ligamentous and Capsular restraints to Anterior Posterior and Superior 3 Inferior laxity of the Acromioclavicular joint – a biomechanical study | J Shoulder Elbow Surg | UK | A |
| 2020 | 33010439 | Biomechanical significance of the collateral ligaments in transolecranon 1 fracture2 dislocations | J Shoulder Elbow Surg | USA | B |
| 2020 | 33015212 | The Loop 'n' Tack Knot Biomechanical Analysis of a Novel Suture Technique for Proximal Biceps Tenodesis | Orthop J Sports Med | USA | B |
| 2020 | 33017168 | Lateral Extra-articular Tenodesis Reduces Anterior Cruciate Ligament Graft Force and Anterior Tibial Translation in Response to Applied Pivoting and Anterior Drawer Loads | Am J Sports Med | USA | A |
| 2020 | 33027123 | Functional Flexion Instability After RotatingPlatform Total Knee Arthroplasty | J Bone Joint Surg Am | USA | A |
| 2020 | 33030070 | Biomechanical Comparison of All-Soft Suture Anchor Single-Row vs Double-Row Bridging Construct for Insertional Achilles Tendinopathy | Foot Ankle Int | USA | C |
| 2020 | 33030768 | Biomechanical evaluation of total ankle arthroplasty. Part II: Influence of loading and fixation design on tibial bone–implant interaction | J Orthop Res | USA | C |
| 2020 | 33040208 | Posterior stabilized total knee arthroplasty reproduces natural joint laxity compared to normal in kinematically aligned total knee arthroplasty: a matched pair cadaveric study | Arch Orthop Trauma Surg | Korea | C |
| 2020 | 33043696 | Deep gluteal space anatomy and its relationship with deep gluteal pain syndromes | Hip Int | Portugal | A |
| 2020 | 33044605 | Deep deltoid ligament injury is related to rotational instability of the ankle joint: a biomechanical study | Knee Surg Sports Traumatol Arthrosc | Italy | A |
| 2020 | 33051075 | Influence of the Reamer-Irrigator-Aspirator diameter on femoral bone strength and amount of harvested bone graft –a biomechanical cadaveric study | Injury | Switzerland | C |
| 2020 | 33065214 | A Biomechanical Study of Pivot-Shift and Lachman Translations in Anterior Cruciate Ligament-Sectioned Knees, Anterior Cruciate Ligament-Reconstructed Knees, and Knees With Partial Anterior Cruciate | Arthroscopy | USA | B |
| 2020 | 33065664 | The Fibular Intramedullary Nail Versus Locking Plate and Lag Screw Fixation in the Management of Unstable Elderly Ankle Fractures: A Cadaveric Biomechanical Comparison | J Orthop Trauma | UK | B |
| 2020 | 33065667 | Biomechanical Testing of a Transmetatarsal Base Screw in Lisfranc Injuries | J Orthop Trauma | USA | C |
| 2020 | 33073501 | Histologic Observation and Significance of Sympathetic Nerve Fiber Distribution on Human Cervical Ligamentum Flavum | Orthop Surg | China | A |
| 2020 | 33082121 | Stabilization of the distal radioulnar joint by reconstructing the interosseous membrane's distal oblique bundle: Cadaver study | Orthop Traumatol Surg Res | France | C |
| 2020 | 33084006 | Menisco-fibular ligament — an overview: cadaveric dissection, clinical and magnetic resonance imaging diagnosis, arthroscopic visualisation and treatment | Folia Morphol (Warsz) | Poland | C |
| 2020 | 33088839 | Graft Tensioning in Superior Capsular Reconstruction Improves Glenohumeral Joint Kinematics in Massive Irreparable Rotator Cuff Tears A Biomechanical Study of the Influence of Superior Capsular Reco | Orthop J Sports Med | USA | B |
| 2020 | 33090018 | Biomechanical Comparison of a Novel Method of Tricortical Kirschner Wire Fixation of Distal Chevron Osteotomies of the First Metatarsal Versus Traditional Kirschner Wire and Versus Screw Fixation | Foot Ankle Spec | USA | B |
| 2020 | 33096230 | External bone size identifies different strength-decline trajectories for the male human femora | J Struct Biol | USA | A |
| 2020 | 33097200 | Double column acetabular fractures fixation using a novel dynamic anterior plate-screw system: A biomechanical analysis | Injury | China | A |
| 2020 | 33106035 | How Much Scaphoid Can be Safely Resected? A Biomechanical Analysis of the Effects of Distal Scaphoid Resection | Hand (N Y) | Canada | A |
| 2020 | 33110927 | Prospective Frontal Plane Angles Used to Predict ACL Strain and Identify Those at High Risk for Sports-Related ACL Injury | Orthop J Sports Med | USA | A |
| 2020 | 33111577 | Development of an In Vitro Swan Neck Deformity Biomechanical Model | Hand (N Y) | Canada | A |
| 2020 | 33122787 | Comprehensive biomechanical analysis of three clinically used fixation constructs for posterior malleolar fractures using cadaveric and finite element analysis | Sci Rep | China | A |
| 2020 | 33126215 | Ultra–high-molecular-weight polyethylene sublaminar tape as semirigid fixation or pedicle screw augmentation to prevent failure in long-segment spine surgery: an ex vivo biomechanical study | J Neurosurg Spine | The Netherlands | B |
| 2020 | 33132624 | Loading at the distal radius and ulna during active simulated dart throw motion | J Orthop | Canada | C |
| 2020 | 33135534 | Biomechanical evaluation of axial-loading simulated experiment in wrist fractures: a finite element analysis | J Int Med Res | China | A |
| 2020 | 33135907 | Comparison of Ligament Isometry and Patellofemoral Contact Pressures for Medial Patellofemoral Ligament Reconstruction Techniques in Skeletally Immature Patients | Am J Sports Med | USA | A |
| 2020 | 33146417 | Biomechanical Evaluation of Total Ankle Arthroplasty. Part I: Joint Loads during Simulated Level Walking | J Orthop Res | USA | C |
| 2020 | 33150191 | Biomechanics of Ankle Ligament Reconstruction A Cadaveric Study to Compare Stability of Reconstruction Techniques Using 1 or 2 Fibular Tunnels | Orthop J Sports Med | Switzerland | A |
| 2020 | 33157322 | Analysis of the influence of species, intervertebral disc height and Pfirrmann classification on failure load of an injured disc using a novel disc herniation model | Spine J | USA | C |
| 2020 | 33161779 | Biomechanical Comparison of 2 Common Techniques of Minimally Invasive Hallux Valgus Correction | Foot Ankle Int | USA | B |
| 2020 | 33168276 | Biomechanical Effects of Radioscapholunate Fusion With Distal Scaphoidectomy and Triquetrum Excision on Dart-Throwing and Wrist Circumduction Motions | J Hand Surg Am | Japan | C |
| 2020 | 33180193 | A biomechanical comparison of crossed and parallel rod configurations in atlantoaxial internal fixation | Eur Spine J | China | B |
| 2020 | 33203231 | Effect of Lateral Column Lengthening on Subtalar Motion in a Cadaveric Model | Foot Ankle Int | USA | C |
| 2020 | 33207146 | The Effect of Rotation on Intraoperative Fluoroscopic Evaluation of Hindfoot Alignment and How to Help Prevent Error | Bull Hosp Jt Dis (2013) | USA | C |
| 2020 | 33221933 | Medial meniscal extrusion greater than 4 mm reduces medial tibiofemoral compartment contact area: a biomechanical analysis of tibiofemoral contact area and pressures with varying amounts of meniscal ex | Knee Surg Sports Traumatol Arthrosc | USA | B |
| 2020 | 33222233 | Biomechanical evaluation of a modified proximal humeral locking plate application for distal extra-articular diaphyseal humeral fractures | J Orthop Res | Korea | C |
| 2020 | 33226482 | Importance of the epiphyseal ring in OLIF stand-alone surgery: a biomechanical study on cadaveric spines | Eur Spine J | China | A |
| 2020 | 33244907 | Biomechanical comparison of conventional double-row repair versus double row repair with the parachute configuration | Acta Ortop Mex | Mexico | C |
| 2020 | 33251560 | Radio-luno-triquetral bone-ligament transfer as an additional stabilizer in scapholunate-instability | Arch Orthop Trauma Surg | Switzerland | B |
| 2020 | 33251621 | Is load control necessary to produce physiological AP displacement and axial rotation in wear testing of TAR? | J Orthop Res | USA | B |
| 2020 | 33270464 | Effect of Slope and Varus Correction High Tibial Osteotomy in the ACL-Deficient and ACL-Reconstructed Knee on Kinematics and ACL Graft Force | Am J Sports Med | USA | A |
| 2020 | 33270745 | Comparison of the double loop knot stitch and Kessler stitch for Achilles tendon repair: A biomechanical cadaver study | PLoS One | Germany | A |
| 2020 | 33272074 | Hamstring autograft and anatomical footprint evaluation for anterior talofibular ligament reconstruction: Cadaveric study | J Orthop Surg (Hong Kong) | Turkey | A |
| 2020 | 33276162 | Biomechanical assessment of docking ulnar collateral ligament reconstruction after failed ulnar collateral ligament repair with suture augmentation | J Shoulder Elbow Surg | USA | B |
| 2020 | 33279324 | Biomechanical Comparison of Tendon Coaptation Methods With a Meshed Suture Construct | J Hand Surg Am | USA | A |
| 2020 | 33288803 | Post-operative fracture risk assessment following tumor curettage in the distal femur: a hybrid in vitro and in silico biomechanical approach | Sci Rep | Iran | A |
| 2020 | 33303299 | Effects of peripheral tears of the triangular fibrocartilage complex on distal radioulnar joint stability: A biomechanical study | J Orthop Sci | Japan | C |
| 2020 | 33303325 | The Effect of Forearm Position on Wrist Joint Biomechanics | J Hand Surg Am | Canada | C |
| 2020 | 33307149 | Superior Capsule Reconstruction Using Fascia Lata Allograft Compared With Double- and Single-Layer Dermal Allograft: A Biomechanical Study | Arthroscopy | USA | B |
| 2020 | 33310268 | Assessment of plantaris and peroneus tertius tendons as graft materials for ankle ligament reconstructions – A cadaveric biomechanical study | J Mech Behav Biomed Mater | New Zealand | B |
| 2020 | 33330732 | Biomechanics in an Incomplete Versus Complete Supraspinatus Tear A Cadaveric Study | Orthop J Sports Med | USA | B |

| | | | | | |
|---|---|---|---|---|---|
| 2020 | 33331664 | Biomechanical analysis of arthroscopically assisted latissimus dorsi transfer fixation for irreparable posterosuperior rotator cuff tears—Knotless versus knotted anchors | J Orthop Res | Switzerland | A |
| 2020 | 33344168 | Comminuted patellar fractures: The role of biplanar fixed angle plate constructs | J Orthop Translat | Switzerland | C |
| 2020 | 33345215 | Double-screw and quadruple-button fixation for the glenoid: Latarjet versus bone block applications | JSES Int | Canada | C |
| 2020 | 33345223 | All-suture anchor and unicortical button show comparable biomechanical properties for onlay subpectoral biceps tenodesis | JSES Int | USA | B |
| 2020 | 33345618 | Effect of Oblique Tendon Laceration on Core Suture Strength: A Biomechanical Evaluation | Hand (N Y) | USA | A |
| 2020 | 33349538 | The Lapidus Arthrodesis: Examining the Effect of the Metatarsal Base Transfixion Screw | J Foot Ankle Surg | USA | C |
| 2020 | 33357985 | Use of Thermoplastic Rings Following Venting of Flexor Tendon Pulleys: A Biomechanical Analysis | J Hand Surg Am | Canada | C |
| 2020 | 33358881 | The Ideal Insertion Site for the Flexor Digitorum Profundus Tendon in Jersey Finger Repair: A Biomechanical Analysis | J Hand Surg Am | USA | C |
| 2020 | 33359398 | Low-profile double plating of unstable osteoporotic olecranon fractures: a biomechanical comparative study | J Shoulder Elbow Surg | Germany | A |
| 2020 | 33359853 | Anterior Cable Reconstruction of the Superior Capsule Using Semitendinosus Allograft for Large Rotator Cuff Defects Limits Superior Migration and Subacromial Contact Without Inhibiting Range of Moti | Arthroscopy | USA | C |
| 2020 | 33424187 | Experimental and clinical analysis of the use of asymmetric vs symmetric polyethylene inserts in a mobile bearing total knee arthroplasty | J Orthop | Belgium | C |
| 2020 | 33426851 | Comparison of Biomechanical Properties of a Synthetic L3-S1 Spine Model and Cadaveric Human Samples | J Long Term Eff Med Implants | USA | C |
| 2020 | 33426852 | Biomechanical Comparison between Volar Plate Fixator and Nonbridge External Wrist Fixator | J Long Term Eff Med Implants | USA | C |
| 2020 | 33426853 | Cervical Spine Fusion: Biomechanics of a Three-Level Cadaver Model Comparing Anterior Plate versus Stand-Alone Cage | J Long Term Eff Med Implants | USA | B |
| 2020 | 33431315 | Comparison of Open Anterior Syndesmotic Repair Augmented With SutureTape and Trans-syndesmotic Screw Fixation: A Biomechanical Study | J Foot Ankle Surg | Korea | A |
| 2020 | 33482371 | Dermal allograft superior capsule reconstruction biomechanics and kinematics | J Shoulder Elbow Surg | USA | C |
| 2020 | 33485511 | Development of customized finite element models of medial column fixation using an intramedullary beam: A computational sensitivity analysis | Med Eng Phys | USA | C |
| 2020 | 33508758 | The moment arms and leverage of the human finger muscles | J Biomech | Australia | C |
| 2020 | 33579590 | Evaluation of A2 and A4 Hand Pulley Repair Using Tendon Graft Rings | J Hand Surg Am | USA | C |
| 2020 | 33614603 | Cyclic Damage Accumulation in the Femoral Constructs Made With Cephalomedullary Nails | Front Bioeng Biotechnol | USA | B |
| 2020 | 33681849 | Latissimus dorsi tendon transfer in reverse shoulder arthroplasty: transfer location affects strength | JSES Int | Canada | B |
| 2020 | 33715837 | A Novel Construct Incorporating C2 Unilateral Pedicle and Contralateral Translaminar Screws for Occipitocervical Internal Fixation: An In Vitro Biomechanical Study | World Neurosurg | China | A |
| 2020 | 33766437 | Carpal Motion in Chronic Geissler IV Scapholunate Interosseous Ligament Wrists | J Hand Surg Am | USA | C |
| 2020 | 33832979 | Lateral tenodesis procedures increase lateral compartment pressures more than anterolateral ligament reconstruction, when performed in combination with ACL reconstruction: a pilot biomechanical study | J ISAKOS | Australia | A |
| 2020 | 33832980 | Different anterolateral procedures have variable impact on knee kinematics and stability when performed in combination with anterior cruciate ligament reconstruction | J ISAKOS | Australia | A |
| 2020 | 33860030 | The Alveolar Ridge Splitting Technique on Maxillae: A Biomechanical Human Cadaveric Investigation | Biomed Res Int | Germany | C |
| 2020 | 34804210 | Reverse shoulder arthroplasty glenoid lateralization influences scapular spine strains | Shoulder Elbow | Canada | B |
| 2020 | 34804216 | Biomechanics of axial load transmission across the native human elbow | Shoulder Elbow | USA | B |
| 2020 | 35097361 | Biomechanical Evaluation With a Novel Cadaveric Model Using Supination and Pronation Testing of a Lisfranc Ligament Injury | Foot Ankle Orthop | Chile | C |
| 2020 | 35097388 | Effect of Lateral Sliding Calcaneus Osteotomy on Tarsal Tunnel Pressure | Foot Ankle Orthop | Switzerland | B |
| 2020 | 35097413 | Clinical Outcomes and Cadaveric Biomechanical Analysis of Endoscopic Percutaneous Achilles Tendon Rupture Repair With Absorbable Suture | Foot Ankle Orthop | USA | A |
| 2020 | 35097415 | Biomechanical Evaluation of a New Suture Button Technique for Reduction and Stabilization of the Distal Tibiofibular Syndesmosis | Foot Ankle Orthop | USA | A |
| 2020 | 35141586 | ICR in human cadaveric specimens: An essential parameter to consider in a new lumbar disc prosthesis design | N Am Spine Soc J | Spain | B |
| 2020 | 35154399 | Effect of human dermal allograft thickness on glenohumeral stability for superior capsular reconstruction in irreparable supraspinatus tears: A biomechanical analysis of the superior capsular reconstruction – | Shoulder Elbow | Australia | A |
| 2020 | 35236456 | The accuracy of femoral component rotational measurements using computed tomography—a cadaveric study | Arthroplasty | UK | B |
| 2020 | 35415484 | Measurement of the Material Properties of the Triangular Fibrocartilage Complex | J Hand Surg Glob Online | Japan | B |
| 2020 | 35415522 | The Effect of Flexor Digitorum Profundus Dynamic Tenodesis on the Distal Interphalangeal Joint: A Cadaver Study | J Hand Surg Glob Online | Japan | C |
| 2020 | 35415529 | Dorsal BoneeLigamenteBone Reconstruction of Chronic Lunotriquetral Instability: Biomechanical Testing | J Hand Surg Glob Online | Switzerland | B |
| 2020 | 36556953 | Guided-Motion Bicruciate-Stabilized Total Knee Arthroplasty Reproduces Native Medial Collateral Ligament Strain | Medicina (Kaunas) | Korea | C |
| 2020 | 31377827 | Varus alignment aggravates tibiofemoral contact pressure rise after sequential medial meniscus resection | Knee Surg Sports Traumatol Arthrosc | Germany | A |
| 2020 | 32339739 | Anatomic Relationship of Bony Structures in Pedicle-Rib Unit and its Significance | World Neurosurg | China | A |
| 2020 | 33010438 | Higher primary stability of tuberosity fixation in reverse fracture arthroplasty with 135° than with 155° humeral inclination | J Shoulder Elbow Surg | Germany | A |
| 2020 | 33263782 | Suturing methods in prolapse surgery: a biomechanical analysis | Int Urogynecol J | Germany | B |
| 2021 | 32189522 | Can Syndesmosis Screws Displace the Distal Fibula? | Foot Ankle Spec | USA | B |
| 2021 | 32453125 | Direct vertebral rotation significantly decreases the pullout strength of the pedicle screw: a biomechanical study in adult cadavers | J Pediatr Orthop B | Turkey | B |
| 2021 | 32568130 | Is the strain pattern of conventional stems negatively affected by a previously short stem THA? An experimental study in cadavaric bone | Technol Health Care | Germany | B |
| 2021 | 32590411 | An Isolated Transosseous Flexible Suture Frame in the Treatment of Patellar Tendon Rupture Provides Adequate Mechanical Resistance | J Am Acad Orthop Surg | Chile | A |
| 2021 | 32833697 | Do Fully Threaded Transiliac–Transsacral Screws Improve Mechanical Stability of Vertically Unstable Pelvic Fractures? | J Orthop Trauma | USA | C |
| 2021 | 33010438 | Higher primary stability of tuberosity fixation in reverse fracture arthroplasty with 135°than with 155° humeral inclination | J Shoulder Elbow Surg | Germany | A |
| 2021 | 33038176 | Comparison of the Biomechanical Stiffness of Titanium and Sonic Weld RX Osteofixation Systems for Monoblock Zygomaticomaxillary Complex Fractures | J Craniofac Surg | Japan | B |
| 2021 | 33165635 | Small lateral meniscus tears propagate over time in ACL intact and deficient knees | Knee Surg Sports Traumatol Arthrosc | USA | A |
| 2021 | 33252446 | Optimal Fixation of the Capitellar Fragment in Distal Humerus Fractures | J Orthop Trauma | USA | C |
| 2021 | 33263782 | Suturing methods in prolapse surgery: a biomechanical analysis | Int Urogynecol J | Germany | A |
| 2021 | 33315360 | Comprehensive Evaluation of Accessory Rod Position, Rod Material and Diameter, Use of Cross-connectors, and Anterior Column Support in a Pedicle Subtraction Osteotomy Model | Spine (Phila Pa 1976) | USA | C |
| 2021 | 33320296 | Multimodal control of neck muscles for vestibular mediated head oscillation damping during walking: a pilot study | Eur Arch Otorhinolaryngol | Germany | A |
| 2021 | 33337686 | Biomechanical Contributions of Spinal Structures with Different Degrees of Disc Degeneration | Spine (Phila Pa 1976) | Switzerland | A |
| 2021 | 33369973 | Altered Glenohumeral Biomechanics in ProximalHumeral Fracture Malunion | J Am Acad Orthop Surg | USA | C |
| 2021 | 33381993 | Microstructural and Mechanical Properties of the Anterolateral Ligament of the Knee | Am J Sports Med | USA | B |
| 2021 | 33386885 | Biomechanical evaluation of three patellar fixation techniques for MPFL reconstruction: Load to failure did not differ but interference screw stabilization was stiffer than suture anchor and suture-knot fixatio | Knee Surg Sports Traumatol Arthrosc | Greece | A |
| 2021 | 33388826 | Suture tape augmentation improves laxity of MCL repair in the ACL reconstructed knee | Knee Surg Sports Traumatol Arthrosc | USA | A |
| 2021 | 33393435 | The Anatomical and Biomechanical Superiority of Novel Posterior En Bloc Elevation Cervical Laminoplasty | Surg Innov | China | C |
| 2021 | 33395317 | Comparison of Different Fixation Techniques of the Long Head of the Biceps Tendon in Superior Capsule Reconstruction for Irreparable Posterosuperior Rotator Cuff Tears | Am J Sports Med | USA | B |
| 2021 | 33407705 | The impact of different artificial disc heights during total cervical disc replacement: an in vitro biomechanical study | J Orthop Surg Res | China | A |
| 2021 | 33412955 | Single Versus Dual Headless Compression Screw Fixation of Scaphoid Nonunions: A Biomechanical Comparison | Hand (N Y) | USA | A |

| | | | | | |
|---|---|---|---|---|---|
| 2021 | 33418091 | Superior capsule reconstruction using a single 6-mm-thick acellular dermal allograft for massive rotator cuff tears: a biomechanical cadaveric comparison to fascia lata allograft | J Shoulder Elbow Surg | USA | A |
| 2021 | 33423850 | Minimally Invasive Intramedullary Screw Versus Plate Fixation for Proximal Phalanx Fractures: A Biomechanical Study | J Hand Surg Am | USA | C |
| 2021 | 33430913 | A biomechanics study on ligamentous injury in anterior-posterior compression type II pelvic injury | J Orthop Surg Res | China | A |
| 2021 | 33441095 | Height and volume restoration in osteoporotic vertebral compression fractures: a biomechanical comparison of standard balloon kyphoplasty versus Tektona® in a cadaveric fracture model | BMC Musculoskelet Disord | Germany | A |
| 2021 | 33477002 | Insufficient lateral joint laxity after bicruciate-retaining total knee arthroplasty potentially influences kinematics during flexion: A biomechanical cadaveric study | Knee | Japan | A |
| 2021 | 33479838 | Acromioclavicular joint stabilization with a double cow-hitch technique compared to a double tight-rope: a biomechanical study | Arch Orthop Trauma Surg | Switzerland | A |
| 2021 | 33490294 | Mapping of the Inferior Glenohumeral Ligament for Suture Pullout Strength A Biomechanical Analysis | Orthop J Sports Med | Australia | A |
| 2021 | 33493681 | Biomechanical comparison of semirigid junctional fixation techniques to prevent proximal junctional failure after thoracolumbar adult spinal deformity correction | Spine J | The Netherlands | B |
| 2021 | 33510098 | Preparation of human decellularized peripheral nerve allograft using amphoteric detergent and nuclease | Neural Regen Res | Korea | B |
| 2021 | 33514363 | A novel 3D-printed locking cage for anterior atlantoaxial fixation and fusion: case report and in vitro biomechanical evaluation | BMC Musculoskelet Disord | China | A |
| 2021 | 33516127 | Development of a detailed human neck ffnite element model and injury risk curves under lateral impact | J Mech Behav Biomed Mater | Japan | C |
| 2021 | 33524791 | Dual-laser measurement of human stapes footplate motion under blast exposure | Hear Res | USA | A |
| 2021 | 33529777 | Biomechanical stability of simple coronal shear fracture fixation of the capitellum | J Shoulder Elbow Surg | Switzerland | A |
| 2021 | 33529942 | Overdrilling increases the risk of screw perforation in locked plating of complex proximal humeral fractures – A biomechanical cadaveric study | J Biomech | Switzerland | C |
| 2021 | 33544626 | A Biomechanical, Cadaveric Evaluation of Single- Versus Double-Row Repair Techniques on Stability of Bony Bankart Lesions | Am J Sports Med | USA | B |
| 2021 | 33545680 | Impact of dual-headed pedicle screws on the biomechanics of lumbosacral junction multirod constructs | J Neurosurg Spine | USA | C |
| 2021 | 33548768 | Material properties and application of biomechanical principles provide significant motion control performance in experimental ankle foot orthosis-footwear combination | Clin Biomech (Bristol, Avon) | USA | C |
| 2021 | 33549426 | Biomechanical Comparison of Tape Versus Suture in Simulated Achilles Tendon Midsubstance Rupture | J Foot Ankle Surg | USA | C |
| 2021 | 33553457 | Measurements of Tendon Movement Within the Bicipital Groove After Suprapectoral Intra-articular Biceps Tenodesis in a Cadaveric Model | Orthop J Sports Med | USA | B |
| 2021 | 33559576 | A Biomechanical Comparison of Locked Unicortical with Non-Locking Bicortical Screws for Metacarpal Shaft Fractures in a Randomized Human Cadaveric Model | J Hand Surg Asian Pac Vol | New Zealand | C |
| 2021 | 33560867 | Forces at the Anterior Meniscus Attachments Strongly Increase Under Dynamic Knee Joint Loading | Am J Sports Med | Germany | A |
| 2021 | 33571652 | Does dual plating clavicle fractures increase the risk of refracture after hardware removal? A biomechanical investigation | J Shoulder Elbow Surg | USA | B |
| 2021 | 33582598 | Bone cement augmentation of femoral nail head elements increases their cut-out resistance in poor bone quality– A biomechanical study | J Biomech | Belgium | B |
| 2021 | 33610165 | Three-dimensional analysis of anterior talofibular ligament strain patterns during cadaveric ankle motion using a miniaturized ligament performance probe | BMC Musculoskelet Disord | Japan | A |
| 2021 | 33614795 | Adjustable Cortical Fixation Device for Quadriceps Tendon Repair A Cadaveric Biomechanical Study | Orthop J Sports Med | USA | C |
| 2021 | 33614806 | Interbundle Impingement Pressure in Individualized and Nonindividualized Double-Bundle Anterior Cruciate Ligament Reconstruction A Cadaveric Study | Orthop J Sports Med | China | B |
| 2021 | 33615270 | Suture Tape Augmentation Has No Effect on Anterior Tibial Translation, Gap Formation, or Load to Failure of Anterior Cruciate Ligament Repair: A Biomechanical Pilot Study | Arthrosc Sports Med Rehabil | USA | B |
| 2021 | 33621646 | H-Plasty Repair Technique Improved Tibiofemoral Contact Mechanics After Repair for Adjacent Radial Tears of Posterior Lateral Meniscus Root: A Biomechanical Study | Arthroscopy | China | A |
| 2021 | 33623800 | The Role of the Acromioclavicular Ligament in Acromioclavicular Joint Stability A Cadaveric Biomechanical Study | Orthop J Sports Med | Thailand | A |
| 2021 | 33626366 | A Biomechanical Study of Flanged Intrascleral Haptic Fixation of Three-Piece Intraocular | Am J Ophthalmol | USA | B |
| 2021 | 33638741 | Removal of cement-augmented screws in distal femoral fractures and the effect of retained screws and cement on total knee arthroplasty: a biomechanical investigation | J Orthop Traumatol | Germany | C |
| 2021 | 33639560 | Viscoelastic and failure properties of two configurations of triple-folded hamstring tendons used for anterior cruciate ligament (ACL) reconstruction | Knee | USA | B |
| 2021 | 33656943 | Effect of Percentage of Femoral Anterior Cruciate Ligament Insertion Site Reconstructed With Hamstring Tendon on Knee Kinematics and Graft Force | Am J Sports Med | USA | A |
| 2021 | 33660678 | The Role of Vertebral Porosity and Implant Loading Mode on Bone-Tissue Stress in the Human Vertebral Body Following Lumbar Total Disc Arthroplasty | Spine (Phila Pa 1976) | USA | C |
| 2021 | 33675968 | Biomechanical evaluation of 2 techniques of repair after subscapularis peel for stemless shoulder arthroplasty | J Shoulder Elbow Surg | USA | B |
| 2021 | 33677624 | The Medial structures of the knee have a significant contribution to posteromedial rotational laxity control in the PCL-deficient knee | Knee Surg Sports Traumatol Arthrosc | Canada | B |
| 2021 | 33679021 | The effect of metaglene lateralization on joint mobility of reverse shoulder arthroplasty: A cadaveric biomechanical study | J Orthop | Germany | A |
| 2021 | 33679035 | The effect of four-corner fusion and proximal row carpectomy on uniplanar and multiplanar wrist motion: A biomechanical study | J Orthop | Canada | C |
| 2021 | 33689503 | Posterior Tibial Loading Results in Significant Increase of Peak Contact Pressure in the Patellofemoral Joint During Anterior Cruciate Ligament Reconstruction: A Cadaveric Study | Am J Sports Med | Hong Kong | A |
| 2021 | 33705893 | Double-Bundle Anterior Cruciate Ligament Reconstruction With Lateral Extra-Articular Tenodesis Is Effective in Restoring Knee Stability in a Chronic, Complex Anterior Cruciate LigamentInjured KneeM | Arthroscopy | Korea | B |
| 2021 | 33720794 | Arthroscopic triangular fibrocartilage complex repair: A biomechanical comparison of anchor and pull-out techniques in Atzei type II lesions | Sci Prog | Chile | A |
| 2021 | 33722233 | Cadaveric biomechanical analysis of multilevel lateral lumbar interbody fusion with and without supplemental instrumentation | BMC Musculoskelet Disord | China | A |
| 2021 | 33739308 | An Unrecognized Ligament and its Ossification in the Craniocervical Junction: Prevalence, Patient Characteristics, and Anatomic Evidence | Clin Orthop Relat Res | China | A |
| 2021 | 33745699 | Biomechanical Comparison of Fibertape Device Repair Techniques of Ligamentous Lisfranc Injury in a Cadaveric Model | Injury | USA | B |
| 2021 | 33748273 | Is Bridge Plating of Comminuted Humeral Shaft Fractures Advantageous When Using Compression Plates with Three versus Two Screws per Fragment? A Biomechanical Cadaveric Study | Biomed Res Int | Switzerland | C |
| 2021 | 33755035 | In-vitro 3D Analysis of Sacroiliac Joint Kinematics | Spine (Phila Pa 1976) | USA | C |
| 2021 | 33755800 | Biomechanics of a cemented short stem: a comparative in vitro study regarding primary stability and maximum fracture load | Arch Orthop Trauma Surg | Germany | A |
| 2021 | 33755927 | Biomechanical analysis of the number of implants for the immediate sacroiliac joint fixation | Spine Deform | Canada | A |
| 2021 | 33763498 | Interference Screw Versus Suture Anchors for Femoral Fixation in Medial Patellofemoral Ligament Reconstruction A Biomechanical Study | Orthop J Sports Med | USA | B |
| 2021 | 33765570 | The role of the transversal ligament on the atlantoaxial complex – Bending forces at C1/2 flexion limits in the elderly | Clin Biomech (Bristol, Avon) | Germany | A |
| 2021 | 33766480 | Effect of Ankle Motion and Tensile Stress at the Achilles Tendon on the Contact Pressure Between the Achilles Tendon and the Calcaneus | J Foot Ankle Surg | Japan | C |
| 2021 | 33770037 | Biomechanical Impact of a Zygoma Complex Fracture Using Human Cadaver | J Craniofac Surg | Canada | A |
| 2021 | 33773576 | Biomechanical study of Tile C3 pelvicfracture fixation using an anterior internalsystem combined with sacroiliac screws | J Orthop Surg Res | China | B |
| 2021 | 33774167 | Biomechanical comparison of zoned-conformity glenoid versus standard glenoid in total shoulder arthroplasty: impact on rotator cuff strain and glenohumeral translation | J Shoulder Elbow Surg | USA | B |
| 2021 | 33812034 | Pie-Crusting Capsulotomy Provides Similar Visualization With Increased Repair Stiffness Compared With a T-Capsulotomy: A Biomechanical Study | Arthroscopy | USA | B |
| 2021 | 33812094 | Percutaneous Reconstruction of the Medial Patello-Femoral Ligament Using Dynamic Gracilis Tendon Transfer | Orthop Traumatol Surg Res | France | B |
| 2021 | 33812917 | Incomplete Rotator Cable Did Not Cause Rotator Cuff Dysfunction in Case of Rotator Cuff Tear: A Biomechanical Study of the Relationship Between Rotator Cable Integrity and Rotator Cuff Function | Arthroscopy | China | B |
| 2021 | 33815952 | In vitro Biomechanical Analysis of ProximalPhalangeal Osteotomy Fixation | J Wrist Surg | Belgium | A |
| 2021 | 33819945 | Biomechanics of Circumferential Cervical Fixation Using Posterior Facet Cages: A Cadaveric Study | Neurospine | USA | C |
| 2021 | 33827991 | Dimensions of pes anserinus of the lower extremity, an anatomical study with its surgical implications | Anat Cell Biol | India | A |
| 2021 | 33836491 | Biomechanical comparison of arthroscopic and open lunate excisions in the cadaveric wrist | Clin Biomech (Bristol, Avon) | Thailand | A |
| 2021 | 33839898 | Glenoid concavity has a higher impact on shoulder stability than the size of a bony defect | Knee Surg Sports Traumatol Arthrosc | Germany | A |
| 2021 | 33845833 | Augmented cerclage wire improves the fixation strength of a two-screw construct for humerus split type greater tuberosity fracture: a biomechanical study | BMC Musculoskelet Disord | Taiwan | A |
| 2021 | 33853155 | Biomechanical Evaluation of Anterolateral Ligament Repair Augmented with Internal Brace | J Knee Surg | USA | B |

| | | | | | |
|---|---|---|---|---|---|
| 2021 | 33872064 | Intratendinous Strain Variations of the Supraspinatus Tendon Depending on Repair Technique A Biomechanical Analysis Regarding the Cause of Medial Cuff Failure | Am J Sports Med | Germany | A |
| 2021 | 33872461 | Tissue-engineering the larynx: Effect of decellularization on human laryngeal framework and the cricoarytenoid joint | J Biomed Mater Res B Appl Biomater | USA | B |
| 2021 | 33877380 | The contralateral knee is a good predictor for determining normal knee stability: a cadaveric study | Knee Surg Sports Traumatol Arthrosc | Belgium | A |
| 2021 | 33879133 | Standardized artificially created stable pertrochanteric femur fractures present more homogenous results compared to osteotomies for orthopaedic implant testing | BMC Musculoskelet Disord | Switzerland | A |
| 2021 | 33880251 | Double Tension Slide Technique as a Novel Repair for Distal Biceps Tendon Tear: A Biomechanical Evaluation | Cureus | USA | B |
| 2021 | 33884453 | Anatomical versus non-anatomical configuration of double coraco-clavicular tunnel technique in acromioclavicular joint reconstruction | Arch Orthop Trauma Surg | Germany | A |
| 2021 | 33889647 | Using the Remnant Anterior Cruciate Ligament to Improve Knee Stability Biomechanical Analysis Using a Cadaveric Model | Orthop J Sports Med | USA | B |
| 2021 | 33890514 | Do Coronal or Sagittal Plane Measurements Have the Highest Accuracy to Arthroscopically Diagnose Syndesmotic Instability? | Foot Ankle Int | USA | C |
| 2021 | 33892119 | A new approach to superior capsular reconstruction with hamstring allograft for irreparable posterosuperior rotator cuff tears: a dynamic biomechanical evaluation | J Shoulder Elbow Surg | USA | B |
| 2021 | 33895297 | Biomechanical analysis of progressive rotator cuff tendon tears on superior stability of the shoulder | J Shoulder Elbow Surg | USA | B |
| 2021 | 33895306 | Increased Load to Failure in Biceps Tenodesis With All-Suture Suture Anchor Compared With Interference Screw: A Cadaveric Biomechanical Study | Arthroscopy | USA | B |
| 2021 | 33895387 | Pull-out strength evaluation of cement augmented iliac screws inosteoporotic spino-pelvic fixation | Orthop Traumatol Surg Res | Germany | A |
| 2021 | 33900989 | The Transverse Process Trajectory Technique: AnAlternative for Thoracic Pedicle Screw Implantation—Radiographic and Biomechanical Analysis | Int J Spine Surg | USA | A |
| 2021 | 33901508 | A Biomechanical Comparison of High-Tensile Strength Tape Versus High-Tensile Strength Suture for Tendon Fixation Under Cyclic Loading | Arthroscopy | USA | A |
| 2021 | 33901629 | Intervertebral disc degeneration relates to biomechanical changes of spinal ligaments | Spine J | Switzerland | A |
| 2021 | 33915439 | Three-dimensional surface strain analyses of simulated defect and augmented spine segments: A biomechanical cadaveric study | J Mech Behav Biomed Mater | USA | A |
| 2021 | 33918435 | Comparison of Three Different Internal Brace Augmentation Techniques for Scapholunate Dissociation: A Cadaveric Biomechanical Study | J Clin Med | Korea | B |
| 2021 | 33928850 | The Mechanical Strength of Side-to-Side Tendon Repair Using a Modified Krackow Technique | J Hand Surg Asian Pac Vol | Japan | A |
| 2021 | 33931786 | A biomechanical comparison of steel screws versus PLLA and magnesium screws for the Latarjet procedure | Arch Orthop Trauma Surg | Germany | B |
| 2021 | 33932828 | Patellar resurfacing has minimal impact on in vitro tibiofemoral kinematics during deep knee flexion in total knee arthroplasty | Knee | USA | C |
| 2021 | 33934922 | The Biomechanical Effects of Simulated Radioscapholunate Fusion with Distal Scaphoidectomy, 4-Corner Fusion with Complete Scaphoidectomy, and Proximal Row Carpectomy Compared to the Native W | J Hand Surg Am | USA | B |
| 2021 | 33938948 | Carpal Arch Changes in Response to Thenar Muscle Loading | J Biomech Eng | USA | C |
| 2021 | 33945819 | Biomechanical Testing of Flanged Polypropylene Sutures in Scleral Fixation | Am J Ophthalmol | USA | B |
| 2021 | 33952236 | Comparison of three different screw trajectories in osteoporotic vertebrae: a biomechanical investigation | BMC Musculoskelet Disord | Germany | A |
| 2021 | 33952413 | Partial Trapeziotrapezoid Resection and Thumb Rangeof Movement After Trapeziometacarpal Joint Fusion—ABiomechanical Study | J Hand Surg Am | Japan | A |
| 2021 | 33954823 | haracterization of the mechanical properties of human parietal bones preserved in modifed larssen solution, formalin and as fresh frozen | Surg Radiol Anat | Turkey | A |
| 2021 | 33958253 | How Does Contact Length Impact Titanium Tapered Splined Stem Stability: A Biomechanical Matched Pair Cadaveric Study | J Arthroplasty | USA | C |
| 2021 | 33960859 | Anatomic and Biomechanical Properties of Flat Medial Patellofemoral Ligament Reconstruction Using an Adductor Magnus Tendon Graft A Human Cadaveric Study | Am J Sports Med | Germany | B |
| 2021 | 33960928 | Interlocked Pins Increase Strength by a LateralSpread of Load in Femoral Neck Fixation: a Cadaver Study | Acta Chir Orthop Traumatol Cech | Norway | A |
| 2021 | 33976433 | Lapidus arthrodesis in combination with arthrodesis of the first metatarsophalangeal joint-biomechanical cadaver study comparing two methods of fixation | Biomed Pap Med Fac Univ Palacky Olomouc Cze | Czech Republic | C |
| 2021 | 33985754 | Supplemental medial small fragment fixation adds stability to distal femur fixation: A biomechanical study | Injury | USA | A |
| 2021 | 33990212 | Does posterior configuration have similar strength as parallel configuration for treating comminuted distal humerus fractures? A cadaveric biomechanical study | BMC Musculoskelet Disord | Taiwan | A |
| 2021 | 33994041 | Differential movement of the median nerve and biceps brachii at the elbow in human cadavers | Clin Biomech (Bristol, Avon) | Spain | A |
| 2021 | 33994083 | Biomechanical Evaluation of a Cadaveric Flatfoot Model and Lateral Column Lengthening Technique | J Foot Ankle Surg | USA | B |
| 2021 | 33994707 | The effect of glenoid lateralization and glenosphere size in reverse shoulder arthroplasty on deltoid load: A biomechanical cadaveric study | J Orthop | Germany | A |
| 2021 | 33997064 | Biomechanical Comparison of an All-Inside Meniscal Repair Device Construct Versus Pullout Sutures for Arthroscopic Transtibial Repair of Posterior Medial Meniscus Root Tears A Matched-Pair Cadaveri | Orthop J Sports Med | Australia | A |
| 2021 | 33997066 | The Effect of Ulnar Collateral Ligament Repair With Internal Brace Augmentation on Articular Contact Mechanics A Cadaveric Study | Orthop J Sports Med | USA | B |
| 2021 | 34001410 | voiding Ring Avulsion Injuries WithSilicone Rings: A Biomechanical Study | J Hand Surg Am | USA | C |
| 2021 | 34001411 | The Effect of Forearm Shortening on Forearm Range of Motion | J Hand Surg Am | USA | A |
| 2021 | 34002642 | Comparison of simulated key pinchafter three surgical procedures fortrapeziometacarpal osteoarthritis:a cadaver study | J Hand Surg Eur Vol | Switzerland | A |
| 2021 | 34016426 | Screw-blade fixation systems for implant anchorage in the femoral head: Horizontal blade orientation provides superior stability | Injury | Germany | C |
| 2021 | 34022860 | Immersion in Raloxifene does not significantly improve bone toughness or screw pull-out strength in multiple in vitro models | BMC Musculoskelet Disord | USA | B |
| 2021 | 34026741 | Osteoarthritis-Related Degeneration Alters the Biomechanical Properties of Human Menisci Before the Articular Cartilage | Front Bioeng Biotechnol | Germany | A |
| 2021 | 34027441 | Biceps Box Configuration for Superior Capsule Reconstruction of the Glenohumeral Joint Decreases Superior Translation but Not to Native Levels in a Biomechanical Study | Arthrosc Sports Med Rehabil | USA | C |
| 2021 | 34027462 | Posterior Lateral Meniscal Root Tears Increase Strain on the Reconstructed Anterior Cruciate Ligament: A Cadaveric Study | Arthrosc Sports Med Rehabil | USA | C |
| 2021 | 34027565 | Evaluation of rotator cuff abduction moment arms for superior capsular reconstruction and reverse total shoulder arthroplasty | Int Orthop | USA | A |
| 2021 | 34052387 | Flat-Tunnel Technique With Independently Tensioned Bundles Better Restores Rotational Stability Than Round-Tunnel Technique in Anatomic Anterior Cruciate Ligament Reconstruction Using Hamstring | Arthroscopy | China | A |
| 2021 | 34052693 | Mechanics of cadaveric anterior cruciate ligament reconstructions during simulated jump landing tasks: Lessons learned from a pilot investigation | Clin Biomech (Bristol, Avon) | USA | A |
| 2021 | 34057540 | Biomechanical performance of bicortical versus pericortical bone trajectory (CBT) pedicle screws | Eur Spine J | Switzerland | A |
| 2021 | 34062224 | Use of the dual construct lowers rod strains in flexion-extension and lateral bending compared to two-rod and two-rod satellite constructs in a cadaveric spine corpectomy model | Spine J | USA | C |
| 2021 | 34078560 | Anatomic Description of the Distal and Intercuneiform Articulations: A Cadaveric Study | J Foot Ankle Surg | USA | C |
| 2021 | 34081548 | Superior Capsule Reconstruction With a 3 mm–Thick Dermal Allograft Partially Restores Glenohumeral Stability in Massive Posterosuperior Rotator Cuff Deficiency A Dynamic Robotic Shoulder Model | Am J Sports Med | USA | C |
| 2021 | 34087457 | Impact of Screw Diameter on Pedicle Screw Fatigue Strength—A Biomechanical Evaluation | World Neurosurg | Germany | C |
| 2021 | 34087499 | Biomechanical Evaluation of Intraosseous Distal Radioulnar Joint Prosthesis: A Prosthesis Designed Based on Sauv´e-Kapandji Procedure | Orthop Traumatol Surg Res | Iran | A |
| 2021 | 34087793 | Failure modes and effects analysis of mechanical thrombectomy for stroke discovered in human brains | J Neurosurg | USA | A |
| 2021 | 34101755 | Improving mandibular reconstruction by using topology optimization, patient specific design and additive manufacturing?—A biomechanical comparison against miniplates on human specimen | PLoS One | Germany | A |
| 2021 | 34105379 | Percutaneous Fixation of Unstable Proximal Phalanx Fractures: A Biomechanical Study | Hand (N Y) | USA | A |
| 2021 | 34110933 | Evaluation of the Intact Anterior Talofibular and Calcaneofibular Ligaments, Injuries, and Repairs With and Without Augmentation A Biomechanical Robotic Study | Am J Sports Med | USA | B |
| 2021 | 34115528 | Lateral Elbow Laxity Is Affected by the Integrity of the Radial Band of the Lateral Collateral Ligament Complex A Cadaveric Model With Sequential Releases and Varus Stress Simulating Everyday Activitie | Am J Sports Med | Italy | A |
| 2021 | 34125619 | Knee Joint Line Obliquity Causes Tibiofemoral Subluxation That Alters Contact Areas and Meniscal Loading | Am J Sports Med | UK | A |
| 2021 | 34126221 | Pullout Strength of All-Suture and Metallic Anchors in Repair of Lateral Collateral Ligament Injuries of the Elbow | Arthroscopy | USA | B |
| 2021 | 34136851 | Proximal and mid-thigh fascia lata graft constructs used forarthroscopic superior capsule reconstruction show equivalentbiomechanical properties: an in vitro human cadaver study | JSES Int | Portugal | A |
| 2021 | 34136869 | Role of the transverse ligament of the ulnar collateral ligament of the elbow: a biomechanical study | JSES Int | USA | A |
| 2021 | 34142252 | The effect of proximal tibiofibular joint dislocation on knee mechanics: reduction and fixation matters | Eur J Orthop Surg Traumatol | USA | C |

| | | | | | |
|---|---|---|---|---|---|
| 2021 | 34142573 | Biomechanical Evaluation of Achilles Tendon Midsubstance Repair The Effects of Anchor Angle and Position | Foot Ankle Spec | USA | B |
| 2021 | 34144523 | Biomechanical effects of a novel posteriorly placed sacroiliac joint fusion device integrated with traditional lumbopelvic long-construct instrumentation | J Neurosurg Spine | USA | C |
| 2021 | 34144524 | Adjacent-segment effects of lumbar cortical screw–rod fixation versus pedicle screw–rod fixation with and without interbody support | J Neurosurg Spine | USA | B |
| 2021 | 34154778 | Experimental testing and biomechanical CT analysis of Chinese cadaveric vertebrae with different modeling approaches | Med Eng Phys | China | A |
| 2021 | 34160368 | The Effect of Sacroiliac Fusion and Pelvic Fixation on Rod Strain in Thoracolumbar Fusion Constructs | Spine (Phila Pa 1976) | USA | C |
| 2021 | 34174676 | A biomechanically-guided planning and execution paradigm for osteoporotic hip augmentation: Experimental evaluation of the biomechanics and temperature-rise | Clin Biomech (Bristol, Avon) | USA | B |
| 2021 | 34179204 | No Difference in Ligamentous Strain or Knee Kinematics Between Rectangular or Cylindrical Femoral Tunnels During Anatomic ACL Reconstruction With a Bone–Patellar Tendon–Bone Graft | Orthop J Sports Med | Canada | A |
| 2021 | 34189968 | The Hallux Metatarsophalangeal Capsule: An Anatomic Study With Respect to Percutaneous Hallux Valgus Correction | Foot Ankle Int | USA | B |
| 2021 | 34191355 | Interaction of loading and ligament injuries in subtalar jointinstability quantified by 3D weightbearing computedtomography | J Orthop Res | Germany | B |
| 2021 | 34195657 | High-Strength Suture Tapes Are Biomechanically Stronger Than High-Strength Sutures Used in Rotator Cuff Repair | Arthrosc Sports Med Rehabil | Switzerland | A |
| 2021 | 34199917 | Seeing Beyond Morphology-Standardized Stress MRI to Assess Human Knee Joint Instability | Diagnostics (Basel) | Germany | A |
| 2021 | 34201685 | Variation of the Three-Dimensional Femoral J-Curve in the Native Knee | J Pers Med | Germany | B |
| 2021 | 34214860 | 3D printed clamps for fixation of spinal segments in biomechanical testing | J Biomech | Switzerland | A |
| 2021 | 34214986 | Biomechanics of open versus minimally invasive deformity correction: comparison of stability and rod strain between pedicle subtraction osteotomy and anterior column realignment | J Neurosurg Spine | USA | B |
| 2021 | 34216262 | Using a patella reduced technique while balancing a TKA results in restored physiological strain in the collateral ligaments: an ex vivo kinematic analysis | Arch Orthop Trauma Surg | Belgium | A |
| 2021 | 34236920 | Chondral Damage After Arthroscopic Repair Techniques for Acute Bony Bankart Lesions A Biomechanical Study | Am J Sports Med | USA | B |
| 2021 | 34247847 | Comparison of Lag Versus Nonlag Screw Fixation for Long Oblique Proximal Phalanx Fractures: A Biomechanical Study | J Hand Surg Am | USA | C |
| 2021 | 34253082 | Establishing a New PatientSpecific Implantation Technique for Total Ankle ReplacementAn In Vitro Study | Foot Ankle Spec | Germany | B |
| 2021 | 34268667 | Cervical endplate bone density distribution measured by CT osteoabsorptiometry and direct comparison with mechanical properties of the endplate | Eur Spine J | Japan | A |
| 2021 | 34269757 | Intersegmental Kinematics of the Upper Cervical Spine | Spine (Phila Pa 1976) | Spain | A |
| 2021 | 34274501 | Dual pitch screw design provides equivalent fixation to upsized screw diameter in revision pedicle screw instrumentation: a cadaveric biomechanical study | Spine J | USA | B |
| 2021 | 34278841 | Biomechanical comparison of a C1 posterior arch clamp with C1 lateral mass screws in constructs for C1-C2 fusion | Proc Inst Mech Eng H | Canada | A |
| 2021 | 34289763 | Biomechanical evaluation of an intramedullary clavicle screw in simple oblique and butterfly wedge fractures | Proc Inst Mech Eng H | USA | C |
| 2021 | 34292810 | Posterior Cruciate Ligament Reconstruction With Internal Brace Augmentation Reduces Posterior Tibial Translation Under Cyclic Loading | Orthopedics | USA | B |
| 2021 | 34293522 | Spinous Process Screw Fixation: A Salvage Technique in Subaxial Cervical Spinal Instrumentation | World Neurosurg | China | A |
| 2021 | 34298135 | Fixation Strength of Modified Iliac Screw Trajectory Compared to Traditional Iliac and S2 Alar-Iliac Trajectories: A Cadaveric Study | World Neurosurg | USA | C |
| 2021 | 34303567 | Casting Position for Distal Radius Fractures Changes Radiocarpal Joint Forces: A Cadaveric Study | J Hand Surg Am | USA | C |
| 2021 | 34311291 | Sexual dimorphisms in three-dimensional masticatory muscle attachment morphometry regulates temporomandibular joint mechanics | J Biomech | USA | A |
| 2021 | 34311318 | Examining the novel use of continuous compression implants in clavicle reconstruction: A biomechanical study | Clin Biomech (Bristol, Avon) | USA | C |
| 2021 | 34313138 | CT Osteoabsorptiometry Assessment of Subchondral Bone Density Predicts Intervertebral Implant Subsidence in a Human ACDF Cadaver Model | Global Spine J | USA | B |
| 2021 | 34313397 | Total ankle replacement along with subtalar jointarthrodesis: In-vitro and in-silico biomechanicalinvestigations | Int J Numer Method Biomed Eng | Iran | A |
| 2021 | 34325942 | Is the Dorsal Fiber-Splitting Approach to the Wrist Safe? A Kinematic Analysis and Introduction of the "Window" Approach | J Hand Surg Am | USA | C |
| 2021 | 34327694 | Biomechanical Analysis of Ideal Knee Flexion Angle for ACL Graft Tensioning UtilizingMultiple Femoral and Tibial Tunnel Locations | J Knee Surg | USA | C |
| 2021 | 34329702 | Bursal Acromial Reconstruction (BAR) Using an Acellular Dermal Allograft for Massive, Irreparable Posterosuperior Rotator Cuff Tears: A Dynamic Biomechanical Investigation | Arthroscopy | Germany | B |
| 2021 | 34330561 | Biomechanical Analysis of Abductor Pollicis Longus Lasso Suspensionplasty for Trapeziectomy | J Hand Surg Am | Australia | A |
| 2021 | 34332050 | Intramedullary Unicortical Button and All-Suture Anchors Provide Similar Maximum Strength for Onlay Distal Biceps Tendon Repair | Arthroscopy | USA | A |
| 2021 | 34347534 | Synthetic PVA Osteochondral Implants for the Knee Joint Mechanical Characteristics During Simulated Gait | Am J Sports Med | USA | A |
| 2021 | 34351312 | Do Metaphyseal Cones and Stems Provide Any Biomechanical Advantage for Moderate Contained Tibial Defects in Revision TKA? A Finite-Element Analysis Based on a Cadaver Model | Clin Orthop Relat Res | USA | A |
| 2021 | 34352334 | Biomechanical Assessment of a V-Shaped Semitendinosus Allograft Anterior Cable Reconstruction for Irreparable Rotator Cuff Tears | Arthroscopy | USA | C |
| 2021 | 34352453 | Development and multi-level validation of a computational model to predict traumatic aortic injury | Comput Biol Med | USA | C |
| 2021 | 34358641 | Anterolateral Structure Reconstruction Similarly Improves the Stability and Causes Less Overconstraint in Anterior Cruciate LigamentReconstructed Knees Compared with Modified Lemaire Lateral Extra-a | Arthroscopy | China | B |
| 2021 | 34365039 | High-resolution infrared microspectroscopic characterization 1 of cartilage cell microenvironment | Acta Biomater | Finland | A |
| 2021 | 34378429 | The Effect of the Mobile Subtalar Joint on Calcaneal and Supramalleolar Osteotomies | Foot Ankle Int | Switzerland | C |
| 2021 | 34380394 | The Impact of Scaphoid Malunion on Carpal Motion: An In-Vitro Analysis | J Hand Surg Asian Pac Vol | Canada | C |
| 2021 | 34380409 | In Vitro Comparison between the Pulvertaft Weave and the Modified Core Suture Pulvertaft Weave | J Hand Surg Asian Pac Vol | USA | C |
| 2021 | 34384476 | Evaluation of biomechanical properties onpartial and complete epitendinous suturein human cadaver flexor tendon repair | J Orthop Surg Res | Thailand | A |
| 2021 | 34391955 | Assessment of trapezial prosthetic cup migration: A biomechanical study | Hand Surg Rehabil | Switzerland | A |
| 2021 | 34392435 | Preliminary biomechanical cadaver studyinvestigating a new load-sharing kneeimplant | J Exp Orthop | New Zealand | B |
| 2021 | 34392764 | Biomechanical specimen assessment by low dose biplanar X-ray study of fusion constructions using a posterior lumbar cage with integrated anchors and posterior adjunctive fixators | Comput Methods Biomech Biomed Engin | France | C |
| 2021 | 34398639 | A ''Comma Sign''–Directed Subscapularis Repair in Anterosuperior Rotator Cuff Tears Yields Biomechanical Advantages in a Cadaveric Model | Am J Sports Med | Germany | A |
| 2021 | 34411677 | Biomechanical Analysis of Cadaveric Thoracic Aorta Zones: The Isthmus is the Weakest Region | Ann Vasc Surg | Brazil | A |
| 2021 | 34411682 | Lateral Release With Tibial Tuberosity Transfer Alters Patellofemoral Biomechanics Promoting Multidirectional Patellar Instability | Arthroscopy | USA | B |
| 2021 | 34423057 | Suture Anchor Versus Transosseous Tunnel Repair for Inferior Pole Patellar Fractures Treated With Partial Patellectomy and Tendon Advancement A Biomechanical Study | Orthop J Sports Med | USA | B |
| 2021 | 34424387 | Biomechanical stability of complex coronal plane fracture fixation of the capitellum | Arch Orthop Trauma Surg | Switzerland | A |
| 2021 | 34430562 | Biomechanical testing of three coracoclavicular ligament reconstruction techniques with a 3D printing navigation template for clavicle-coracoid drilling | Ann Transl Med | China | A |
| 2021 | 34430879 | Hamstring Graft Prepared With Suture Tape Is Effective in Anterior Cruciate Ligament Reconstruction: A Biomechanical Analysis | Arthrosc Sports Med Rehabil | Japan | A |
| 2021 | 34433855 | 3D geometry of femoral reaming for bone graft harvesting | Sci Rep | Switzerland | A |
| 2021 | 34441368 | Comprehensive Assessment of Medial Knee Joint Instability by Valgus Stress MRI | Diagnostics (Basel) | Germany | A |
| 2021 | 34441410 | In-Situ Cartilage Functionality Assessment Based on Advanced MRI Techniques and Precise Compartmental Knee Joint Loading through Varus and Valgus Stress | Diagnostics (Basel) | Germany | A |
| 2021 | 34475982 | Axial and para-axial loading response evaluation on human cadaver-harvested lumbar vertebral blocks: In vitro experiment with possible clinical implications for clinical practice | Exp Ther Med | Romania | B |
| 2021 | 34488294 | Scapular spine base fracture with long outside-in superior or posterior screws with reverse shoulder arthroplasty | Clin Shoulder Elb | Turkey | A |
| 2021 | 34490721 | Investigating the effects of flexor tendon shortening on active range of motion after finger tendon repair | Anat Rec (Hoboken) | USA | C |
| 2021 | 34491456 | Biomechanics of a calcar loading and a shortened tapered femoral stem: Comparative in-vitro testing of primary stability and strain distribution | J Exp Orthop | Germany | A |
| 2021 | 34494894 | Biomechanical Analysis of Segmental Medial Meniscal Transplantation in a Human Cadaveric Model | Am J Sports Med | USA | B |

| | | | | | |
|---|---|---|---|---|---|
| 2021 | 34517193 | The role of the brachialis muscle in elbow stability with collateral ligament injury: A biomechanical investigation | Clin Biomech (Bristol, Avon) | Germany | A |
| 2021 | 34523371 | Radial Head Lag A Possible Biomechanical Mechanism for Osteochondritis Dissecans of the Capitellum in Baseball Pitchers | Am J Sports Med | USA | A |
| 2021 | 34524034 | Ability of Medial Patellofemoral Ligament Reconstruction to Overcome Lateral Patellar Motion in the Presence of Trochlear Flattening A Cadaveric Biomechanical Study | Am J Sports Med | USA | A |
| 2021 | 34524037 | Arthroscopic Centralization for Lateral Meniscal Injuries Reduces Laxity in the Anterior Cruciate Ligament-Reconstructed Knee | Am J Sports Med | USA | A |
| 2021 | 34525448 | Biomechanical assessment of two types and two different locations of subtalar arthroereisis implants for flexible flatfoot: A cadaveric study | Clin Biomech (Bristol, Avon) | China | A |
| 2021 | 34534964 | Biomechanics of a laterally placed sacroiliac joint fusion device supplemental to S2 alar-iliac fixation in a long-segment adult spinal deformity construct: a cadaveric study of stability and strain distribution | J Neurosurg Spine | USA | C |
| 2021 | 34541829 | Biomechanical Effects of Lateral Inclination C1 and C2 Pedicle Screws on Atlantoaxial Fixation | Orthop Surg | China | C |
| 2021 | 34551927 | In Vitro Biomechanics of Human Cadaveric Cervical Spines With Mature Fusion | Int J Spine Surg | USA | B |
| 2021 | 34564041 | Validity of repeated-measures analyses of in vitro arthroplasty kinematics and kinetics | J Biomech | UK | A |
| 2021 | 34565554 | Mechanobiology of the arterial tissue from the aortic root to the diaphragm | Med Eng Phys | Iran | A |
| 2021 | 34568774 | Tissue Engineering the Pinna: Comparison and Characterization of Human Decellularized Auricular Biological Scaffolds | ACS Appl Bio Mater | USA | A |
| 2021 | 34573945 | Validation and Optimization of Proximal Femurs Microstructure Analysis Using High Field and Ultra-High Field MRI | Diagnostics (Basel) | France | A |
| 2021 | 34584688 | Biomechanical study of the abductor pollicis longus & extensor pollicis brevis after release and reconstruction of the first dorsal compartment of the wrist | Ann Med Surg (Lond) | Iran | A |
| 2021 | 34591124 | Full thickness quadriceps tendon grafts with bone had similar material properties to bone-patellar tendon-bone and a four-strand semitendinosus grafts: a biomechanical study | Knee Surg Sports Traumatol Arthrosc | USA | C |
| 2021 | 34598153 | Evaluating stability of the craniovertebral junction after unilateral C1 lateral mass resection: implications for the direct lateral approach | J Neurosurg Spine | USA | C |
| 2021 | 34601156 | Biomechanical comparison of tenorrhaphy constructs for tendon reconstructions and transfers | Hand Surg Rehabil | USA | A |
| 2021 | 34610695 | Influence of Lumbar Lordosis on Posterior Rod Strain in Long-Segment Construct During Biomechanical Loading: A Cadaveric Study | Neurospine | USA | B |
| 2021 | 34616854 | Suture Tape Augmentation of Screw Fixation Reduces Fragment Migration in Tibial Tubercle Osteotomy A Biomechanical Study | Orthop J Sports Med | Germany | B |
| 2021 | 34618704 | Does the Choice of Spinal Interbody Fusion Approach Significantly Affect Adjacent Segment Mobility? | Spine (Phila Pa 1976) | USA | B |
| 2021 | 34618789 | Atlantoaxial Non-Fusion Using Biomimetic Artificial Atlanto-Odontoid Joint | Spine (Phila Pa 1976) | China | A |
| 2021 | 34619461 | Does the neutral zone quantification method matter? Efficacy of evaluating neutral zone during destabilization and restabilization in human spine implant testing | J Biomech | Germany | A |
| 2021 | 34626887 | Biomechanical comparison of two medial patellofemoral ligament reconstruction techniques: Quadriceps tendon fixation versus single-tunnel patella fixation with gracilis autograft did not differ in load to fa | Knee | Greece | A |
| 2021 | 34631290 | The Critical Size of Ulnar Styloid Fragment for the DRUJ Stability | J Wrist Surg | Switzerland | A |
| 2021 | 34631293 | Should We Repair the Pronator Quadratus in a Distal Radius Fracture with an Ulnar Styloid Base Fracture? A Biomechanical Study | J Wrist Surg | Switzerland | C |
| 2021 | 34640187 | MDCT-Based Finite Element Analysis for the Prediction of Functional Spine Unit Strength—An In Vitro Study | Materials (Basel) | Germany | A |
| 2021 | 34671689 | Biomechanical Comparison of Capsular Repair, Capsular Shift, and Capsular Plication for Hip Capsular Closure Is a Single Repair Technique Best for All? | Orthop J Sports Med | USA | B |
| 2021 | 34673363 | Biomechanical study of Proximal humeral fracture fixation: Locking plate with medial support screw vs. locking plate with intramedullary fibular graft | Clin Biomech (Bristol, Avon) | Korea | C |
| 2021 | 34677691 | Novel quantification of the regional strain distribution in the anterior cruciate ligament in response to simulated loading using micro-CT imaging | J Exp Orthop | Canada | A |
| 2021 | 34690014 | Double-Screw Osteosynthesis in an Unstable Scaphoid Fracture Model: A Biomechanical Comparison of Two Screw Configurations | J Hand Surg Am | Australia | C |
| 2021 | 34699272 | Kinematic Analysis of Lateral Meniscal Oblique Radial Tears in the Anterior Cruciate Ligament–Deficient Knee | Am J Sports Med | USA | A |
| 2021 | 34699297 | Normalized vertebral-level specific range ofmotion corridors for female spines in rear impact | Traffic Inj Prev | USA | A |
| 2021 | 34702630 | Central Slip Reconstruction With aDistally Based Flexor DigitorumSuperficialis Slip: A Biomechanical Study | J Hand Surg Am | USA | C |
| 2021 | 34704144 | Biomechanical study on the stability and strain conduction of intertrochanteric fracture fixed with proximal femoral nail antirotation versus triangular supporting intramedullary nail | Int Orthop | China | B |
| 2021 | 34709535 | Improved accuracy and fewer outliers with a novel CT-free robotic THA system in matched-pair analysis with manual THA | J Robot Surg | USA | B |
| 2021 | 34712962 | A Weaving Rip-Stop Technique Leads to a Significantly Increased Load to Failure and Reduction in Suture-Tendon Cut-Through in a Biomechanical Model of Rotator Cuff Repair | Arthrosc Sports Med Rehabil | USA | B |
| 2021 | 34714699 | Kinematic Alterations in the Shoulder Complex in Rockwood V Acromioclavicular Injuries During Humerothoracic and Scapulothoracic Movements A Whole-Cadaver Study | Am J Sports Med | Belgium | A |
| 2021 | 34715276 | Addition of the Sartorius Tendon Improves Biomechanics of a Four-Strand Hamstring Anterior Cruciate Ligament Autograft | Arthroscopy | USA | A |
| 2021 | 34721751 | Apparatus for In Vivo Knee Laxity Assessment Using High-Speed Stereo Radiography | J Med Device | USA | C |
| 2021 | 34739600 | Comparison of fibula strut and calcium phosphate cement augmentation of the medial buttress in 2-part proximal humerus fractures reconstruction: a biomechanical study | Eur J Orthop Surg Traumatol | USA | A |
| 2021 | 34748055 | Biomechanical analysis of recently released cephalomedullary nails for trochanteric femoral fracture fixation in a human cadaveric model | Arch Orthop Trauma Surg | Switzerland | A |
| 2021 | 34761697 | Bi-unicondylar arthroplasty : a biomechanics and clinical outcomes study | Bone Joint Res | UK | A |
| 2021 | 34763101 | Optimizing Lumbar Pedicle Screw Trajectory Utilizing a 3D-Printed Drill Guide to Ensure Placement of Pedicle Screws Into Higher Density Bone May Improve Pedicle Screw Pullout Resistance | World Neurosurg | China | C |
| 2021 | 34767953 | Medial Patellofemoral Ligament Reconstruction Using Adductor-Transfer and Adductor-Sling at Nonanatomic Femoral Attachment Sites Leads to Unfavorable Graft-Length Change Patterns: A Descriptive | Arthroscopy | China | B |
| 2021 | 34773149 | The efect of posterior compression of the facet joints for initial stability and sagittal profle in the treatment of thoracolumbar fractures: a biomechanical study | Eur Spine J | Germany | A |
| 2021 | 34778473 | Ultrasonographic Measurement of Elbow Varus Laxity With a Sequential Injury Model of the Lateral Collateral Ligament-Capsular Complex | Orthop J Sports Med | USA | A |
| 2021 | 34778476 | Anterior Glenoid Reconstruction With Distal Tibial Allograft Biomechanical Impact of Fixation and Presence of a Retained Lateral Cortex | Orthop J Sports Med | USA | B |
| 2021 | 34782117 | Angular stable locking in a novel intramedullary nail improves construct stability in a distal tibia fracture model | Injury | Switzerland | C |
| 2021 | 34785299 | Human Dermal Allograft Superior Capsule Reconstruction With Graft Length Determined at Glenohumeral Abduction Angles of 20 and 40 Decreases Joint Translation and Subacromial Pressure Without C | Arthroscopy | USA | C |
| 2021 | 34794043 | Impact of periprosthetic femoral fracture fixation plating constructs on local stiffness, load transfer, and bone strains | J Mech Behav Biomed Mater | USA | C |
| 2021 | 34801659 | Increased Glenohumeral Joint Loads Due to a Supraspinatus Tear Can Be Reversed With Rotator Cuff Repair: A Biomechanical Investigation | Arthroscopy | Germany | B |
| 2021 | 34805106 | Cerebral Vasculature InfluencesBlast-Induced BiomechanicalResponses of Human Brain Tissue | Front Bioeng Biotechnol | USA | C |
| 2021 | 34808351 | Long head of biceps transfer to augment Bankart repair in chronic anterior shoulder instability with and without subcritical bone loss: a biomechanical study | J Shoulder Elbow Surg | USA | B |
| 2021 | 34808426 | Biomechanical analysis of the role of hinge support fixators on hinge stability in medial closing wedge distal femoral osteotomy | Clin Biomech (Bristol, Avon) | Japan | A |
| 2021 | 34821943 | Arthroscopic single anchor repair techniques for upper third subscapularis tears provide sufficient biomechanical stability | Knee Surg Sports Traumatol Arthrosc | Switzerland | A |
| 2021 | 34838644 | Dynamic Double-Sling Augmentation Prevents Anteroinferior Translation for Recurrent Anteroinferior Shoulder Dislocation With 20% Glenoid Bone Loss: A Cadaveric Biomechanical Study | Arthroscopy | China | B |
| 2021 | 34842976 | Tacks vs. sutures: a biomechanical analysis of sacral bony fxation methods for laparoscopic apical fxations in the porcine model | Arch Gynecol Obstet | Germany | A |
| 2021 | 34845538 | A biomechanical analysis of diferent meshes for reconstructions of the pelvic foor in the porcine model | Arch Gynecol Obstet | Germany | A |
| 2021 | 34868493 | Lag Screw Trajectory in Supination-External Rotation Fractures: Does the Direction of the Fibula Lag Screw Have an Effect? | Clin Orthop Surg | USA | A |
| 2021 | 34871896 | Structure-function relationships of TMJ lateral capsule-ligament complex | J Biomech | USA | A |
| 2021 | 34872702 | Standard versus customised locking plates for fixation of schatzker ii tibial plateau fractures | Injury | Singapore | A |
| 2021 | 34875498 | Anterior cruciate ligament (ACL) repair using cortical or anchor fixation with suture tape augmentation vs ACL reconstruction: A comparative biomechanical analysis | Knee | USA | B |
| 2021 | 34879318 | The biomechanical effect of injury and repair of the inferior glenohumeral ligament on glenohumeral stability: Contribution of the posterior band | Clin Biomech (Bristol, Avon) | France | C |
| 2021 | 34881102 | Histomorphology of the Subregions of the Scapholunate Interosseous Ligament and Its Enthesis | J Wrist Surg | UK | B |
| 2021 | 34881107 | Biomechanical Comparison of Modified AdamsBerger and DX technique in DRUJ Reconstruction | J Wrist Surg | Norway | C |

| | | | | | |
|---|---|---|---|---|---|
| 2021 | 34881391 | How relevant is lumbar bone mineral density for the stability of symphyseal implants? A biomechanical cadaver study | Eur J Trauma Emerg Surg | Germany | A |
| 2021 | 34889687 | Biomechanical Comparison of Anatomic Restoration of the Ulnar Footprint vs Traditional Ulnar Tunnels in Ulnar Collateral Ligament Reconstruction | Am J Sports Med | USA | A |
| 2021 | 34893040 | Anatomy and histomorphology of the flexor digitorum profundus enthesis: functional implications for tissue engineering and surgery | BMC Musculoskelet Disord | UK | A |
| 2021 | 34893059 | Is cement-augmented sacroiliac screw fixation with partially threaded screws superior to that with fully threaded screws concerning compression and pull-out force in fragility fractures of the sacrum? – a bio | BMC Musculoskelet Disord | Germany | A |
| 2021 | 34893697 | Restricted cement augmentation in unstable geriatric midthoracic fractures treated by long-segmental posterior stabilization leads to a comparable construct stability | Sci Rep | Germany | B |
| 2021 | 34896317 | Trapeziometacarpal joint loading during key pinch grip:A cadaver study | Hand Surg Rehabil | Switzerland | A |
| 2021 | 34916033 | Clinical and Biomechanical Effects of Femoral Neck Buttress Plate used for Vertical Femoral Neck Fractures | Injury | USA | C |
| 2021 | 34920006 | Combination of Surgical Techniques Restores Multidirectional Biomechanical Stability of Acromioclavicular Joint | Arthroscopy | USA | C |
| 2021 | 34920420 | Assessment of L5–S1 anterior lumbar interbody fusion stability in the setting of lengthening posterior instrumentation constructs: a cadaveric biomechanical study | J Neurosurg Spine | USA | C |
| 2021 | 34920874 | The pectineal ligament is a secondary stabilizer in anterior pelvic ring fractures - a biomechanical study | Injury | Germany | B |
| 2021 | 34931550 | The Addition of a Krackow Rip Stop Suture Augment After Achilles Tendon Debridement for Insertional Achilles Tendinopathy A Biomechanical Study | Foot Ankle Spec | USA | A |
| 2021 | 34949942 | A Cadaver-Based Biomechanical Evaluation of a Novel Posterior Approach to Sacroiliac Joint Fusion: Analysis of the Fixation and Center of the Instantaneous Axis of Rotation | Materials (Basel) | USA | A |
| 2021 | 34952510 | Biomechanical analysis of stand-alone lumbar interbody cages versus 360° constructs: an in vitro and finite element investigation | J Neurosurg Spine | USA | C |
| 2021 | 34955320 | The Effect of Flexor Digitorum Profundus Repair Position Relative to Camper Chiasm on Tendon Biomechanics | J Hand Surg Am | Canada | C |
| 2021 | 34955405 | The role of medial ligaments and tibialis posterior in stabilising the medial longitudinal foot arch: a cadaveric gait simulator study | Foot Ankle Surg | Belgium | A |
| 2021 | 34956486 | Study on biomechanical analysis of two-level cervical Mobi-C and arthrodesis | Am J Transl Res | China | A |
| 2021 | 34977626 | Clavicular-Sided Tears Were the Most Frequent Mode of Failure During Biomechanical Analysis of Acromioclavicular Ligament Complex Failure During Adduction of the Scapula | Arthrosc Sports Med Rehabil | USA | C |
| 2021 | 34977634 | The Glenolabral Articular Disruption Lesion Is a Biomechanical Risk Factor for Recurrent Shoulder Instability | Arthrosc Sports Med Rehabil | Germany | A |
| 2021 | 34977635 | Suture TapeeAugmented Posterior Cruciate Ligament Repair Should Be Tensioned and Fixed at Approximately 100[illegible] Knee Flexion to Prevent Loss of Full Flexion | Arthrosc Sports Med Rehabil | USA | C |
| 2021 | 35032901 | The diagnostic precision of computed tomography for traumatic cervical spine injury: An in vitro biomechanical investigation | Clin Biomech (Bristol, Avon) | Canada | C |
| 2021 | 35035737 | Gender differences in degenerative lumbar scoliosis spine flexibilities | Am J Transl Res | China | B |
| 2021 | 35097462 | First Tarsometatarsal Joint Loading After Sequential Correction of Hallux Valgus Using a Proximal Opening Wedge Metatarsal Osteotomy and Distal Soft Tissue Procedure | Foot Ankle Orthop | USA | B |
| 2021 | 35097471 | Biomechanical Analysis of Single Interference Screw vs Interference Screw With Cortical Button for Flexor Hallucis Longus Transfer | Foot Ankle Orthop | USA | B |
| 2021 | 35127667 | The Effects of Lateral MeniscusPosterior Root Tear and its Repair onKnee Stability of Internal Rotation andForward Shift: A BiomechanicalKinematics Cadaver Study | Front Bioeng Biotechnol | China | A |
| 2021 | 35141689 | Fixation of distal clavicle fractures with coracoclavicular instability:a comparative biomechanical study in human cadavers | JSES Int | Switzerland | A |
| 2021 | 35342527 | Mini-Blade Plate to Obtain Length Across Lateral Malleolus Fractures: Surgical Technique and Biomechanical Evaluation | Indian J Orthop | USA | B |
| 2021 | 35415536 | Prediction of Stress Distribution Applied to the TriangularFibrocartilage Complex: A Finite Element Analysis | J Hand Surg Glob Online | Japan | A |
| 2021 | 35415542 | Biomechanical Evaluation of Opponensplasty for Low Median Palsy: A Cadaver Study | J Hand Surg Glob Online | Japan | C |
| 2021 | 35415560 | Nitinol Staples for Olecranon Osteotomy Fixation, Juxtacortical versusInset, Effect on Biomechanical Stability | J Hand Surg Glob Online | USA | C |
| 2021 | 35676163 | Hybrid screw fixation for femoral neck fractures: Does it prevent mechanical failure? | Injury | USA | C |
| 2021 | 35845237 | Radiocarpal Contact Pressures Are Not Altered after Scapholunate Ligament Tears | J Wrist Surg | USA | B |
| 2021 | 35146033 | Biomechanical Comparison of UCL Repair Using Suspensory Fixation Versus UCL Reconstruction | Orthop J Sports Med | USA | B |
| 2022 | 32423544 | Effect of rotator cuff muscle activation on glenohumeral kinematics: A cadaveric study | J Biomech | USA | B |
| 2022 | 33813991 | Change of wrist motion before and after fixation of the distal radioulnar joint and resection of the distal ulna in a cadaveric model | Mod Rheumatol | Japan | C |
| 2022 | 34001800 | Muscular Forces Responsible for Proximal Humeral Deformity After Fracture | J Orthop Trauma | USA | C |
| 2022 | 34057108 | Automatic modelling of human musculoskeletal ligaments - Framework overview and model quality evaluation | Technol Health Care | Austria | C |
| 2022 | 34491235 | Deep-Freezing Temperatures During Irradiation Preserves the Compressive Strength of Human Cortical Bone Allografts: A Cadaver Study | Clin Orthop Relat Res | Malaysia | A |
| 2022 | 34494983 | Are Knotted or Knotless Techniques Better for Reconstruction of Full-Thickness Tears of the Superior Portion of the Subscapularis Tendon? A Study in Cadavers | Clin Orthop Relat Res | Germany | A |
| 2022 | 34495828 | An in vitro hand simulator for simultaneous control of hand and wrist movements | IEEE Trans Biomed Eng | UK | A |
| 2022 | 34635924 | In-Situ Fracture Tolerance of the Metatarsals During Quasi-Static Compressive Loading of the Human Foot | J Biomech Eng | Canada | A |
| 2022 | 34664322 | Relation between diameter of a lateral screw and pull-out strength in distal clavicle fracture in plates with different geometry: A cadaveric biomechanical study | J Orthop Res | Korea | C |
| 2022 | 34686638 | Is intrathoracic rib plate fixation advantageous over extrathoracic plating? A biomechanical cadaveric study | J Trauma Acute Care Surg | Switzerland | C |
| 2022 | 34817051 | In Vitro Simulation of Shoulder Motion Driven by Three-Dimensional Scapular and Humeral Kinematics | J Biomech Eng | USA | B |
| 2022 | 34951458 | Development and Multisite Assessment of a Novel Shoulder Motion Joint Simulator | J Biomech Eng | USA | C |
| 2022 | 34973090 | Standardized fracture creation in the distal humerus and the olecranon for surgical training and biomechanical testing | Arch Orthop Trauma Surg | Austria | B |
| 2022 | 34981456 | Motion capture evaluation of sagittal spino-pelvic biomechanics after lumbar spinal fusion | Spine Deform | USA | B |
| 2022 | 34983573 | Impacts of external rotators and the ischiofemoral ligament on preventing excessive internal hip rotation: a cadaveric study | J Orthop Surg Res | Japan | A |
| 2022 | 34986047 | Augmentation of Distal Biceps Tendon Ruptures With the Lacertus Fibrosus A Biomechanical Study in a Tendon-Deficient Model | Am J Sports Med | USA | B |
| 2022 | 34996038 | New anterior controllable antedisplacement and fusion surgery for cervical ossification of the posterior longitudinal ligament: a biomechanical study | J Neurosurg Spine | China | A |
| 2022 | 34996409 | Fixation of intraoperative proximal femoral fractures during THA using two versus three cerclage wires - a biomechanical study | BMC Musculoskelet Disord | Germany | A |
| 2022 | 34999263 | Optimizing Cervicothoracic Junction Biomechanics after C7 Pedicle Subtraction Osteotomy: A Cadaveric Study of Stability and Rod Strain | World Neurosurg | USA | C |
| 2022 | 35005047 | The Biomechanical Consequences of Arthroscopic Hip Capsulotomy and Repair in Positions at Risk for Dislocation | Orthop J Sports Med | USA | A |
| 2022 | 35014829 | Finite Element Analysis of a Bionate Ring-Shaped Customized Lumbar Disc Nucleus Prosthesis | ACS Appl Bio Mater | Spain | B |
| 2022 | 35031124 | Biomechanical Comparison of Dual and Posterior Locking Plates in an Ex Vivo Comminuted Olecranon Fracture Model | J Hand Surg Am | USA | B |
| 2022 | 35047647 | Does the Interval Slide Procedure Reduce Supraspinatus Tendon Repair Tension?: A Biomechanical Cadaveric Study | Orthop J Sports Med | Germany | A |
| 2022 | 35051540 | A biomechanical study on the effect of long head of biceps tenotomy on supraspinatus load and humeral head position during shoulder abduction | J Shoulder Elbow Surg | South Africa | A |
| 2022 | 35061084 | Biomechanical stability of short versus long proximal femoral nails in osteoporotic subtrochanteric A3 reverse-oblique femoral fractures: a cadaveric study | Arch Orthop Trauma Surg | Germany | A |
| 2022 | 35061644 | Contributions of Pubic Rami Fracture Morphology and Fixation to Pelvic Ring Stability in Type 1 Lateral Compression Injuries: A Biomechanical Cadaveric Study | J Orthop Trauma | USA | C |
| 2022 | 35061655 | Superficial Deltoid Ligament and Deep Deltoid Ligament Play Equally Important Roles in the Stability of Isolated Lateral Malleolus (OTA/AO 44-B1) Fractures: A Biomechanical Study | J Orthop Trauma | USA | C |
| 2022 | 35064826 | Locking suture repair versus ligament augmentation—a biomechanical study regarding the treatment of acute lateral collateral ligament injuries of the elbow | Arch Orthop Trauma Surg | Germany | A |
| 2022 | 35066121 | The role of the long head of the biceps tendon in posterior shoulder stabilization during forward flexion | J Shoulder Elbow Surg | USA | B |
| 2022 | 35078451 | Weightbearing after combined medial and lateral plate fixation of AO/OTA 41C2 bicondylar tibial plateau fractures: a biomechanical study | BMC Musculoskelet Disord | USA | A |
| 2022 | 35093798 | Biomechanical comparisons of three minimally invasive Achilles tendon percutaneous repair suture techniques | Clin Biomech (Bristol, Avon) | Germany | A |
| 2022 | 35099324 | Biomechanical Assessment of Knee Laxity After a Novel Posterolateral Corner Reconstruction Technique | Am J Sports Med | Canada | B |

| | | | | | |
|---|---|---|---|---|---|
| 2022 | 35102122 | Pedicle Subtraction Osteotomy Construct Optimization | Spine (Phila Pa 1976) | USA | C |
| 2022 | 35104628 | Synergistic effect of the anterolateral ligament and capsule injuries on the knee laxity in anterior cruciate ligament injured knees: A cadaveric study | Orthop Traumatol Surg Res | Korea | B |
| 2022 | 35106351 | Fracture Risk With Patella Resurfacing During Total Knee Arthroplasty After Anterior Cruciate Ligament Reconstruction Using BonePatella-Bone Autograft: A Biomechanical Analysis | Arthroplast Today | USA | B |
| 2022 | 35121348 | Biomechanical comparison of different fixation methods in metatarsal shaft fractures: A cadaver study | Clin Biomech (Bristol, Avon) | Germany | A |
| 2022 | 35144302 | Lateral Translation of the Patella in MPFC Reconstruction: A Biomechanical Study of Three Approaches | J Knee Surg | USA | B |
| 2022 | 35153076 | The Effect of Buttress Plating on Biomechanical Stability of Coronal Shear Fractures of the Capitellum: A Cadaveric Study | J Hand Surg Am | USA | C |
| 2022 | 35157963 | Medial Patellofemoral Complex Reconstruction Techniques Are Not Equivalent at Lower Flexion in the Setting of Patella Alta: A Biomechanical Comparison | Arthroscopy | USA | B |
| 2022 | 35166268 | Suture Augmentation Neutralizes Deforming Muscular Forces in a Simulated 2-Part Osteoporotic Proximal Humeral Fracture Model | J Orthop Trauma | USA | C |
| 2022 | 35167913 | A new all-suture tension band tape fixation technique for simple olecranon fractures versus conventional tension band wire fixation: a comparative biomechanics study | J Shoulder Elbow Surg | Australia | A |
| 2022 | 35176938 | Application of an indentation sensor for the arthroscopic measurement of articular cartilage stiffness | Proc Inst Mech Eng H | Japan | A |
| 2022 | 35181576 | Determinants of fracture type in the proximal femur: Biomechanical study of fresh frozen cadavers and finite element models | Bone | Japan | A |
| 2022 | 35186902 | Three-Dimensional Innate Mobility of the Human Foot on Coronally-Wedged Surfaces Using a Biplane X-Ray Fluoroscopy | Front Bioeng Biotechnol | Japan | A |
| 2022 | 35188812 | Comparison of a Novel Anatomic Technique and the Docking Technique for Medial Ulnar Collateral Ligament Reconstruction | Am J Sports Med | USA | A |
| 2022 | 35189269 | Anatomical study of all carpal and adjoining bones of the wrist using 3D CT reconstruction –Finding the ultimate biomechanical theory | Ann Anat | Germany | A |
| 2022 | 35189401 | Torque acting on biodegradable magnesium screws during intramedullary insertion into a metacarpal bone – a biomechanical study | Hand Surg Rehabil | Switzerland | A |
| 2022 | 35199184 | Medial talar resection: how much remains stable? | Eur J Trauma Emerg Surg | Switzerland | B |
| 2022 | 35221153 | Overhead arm positioning in the rehabilitation of elbow dislocations: An in vitro biomechanical study | J Hand Ther | Canada | C |
| 2022 | 35234131 | The load transfer effect of monopolar and bipolar radial head prosthesis designs on capitellar cartilage: A comparative biomechanical cadaver study | Acta Orthop Traumatol Turc | Turkey | A |
| 2022 | 35237919 | Optic Nerve Sheath Viscoelastic Properties: Re-Examination of Biomechanical Behavior and Clinical Implications | Neurocrit Care | USA | B |
| 2022 | 35238847 | Does the Nice Knot Offer Less Elongation Than the Modified Prusik Knot? An In Vitro Study in Cadaver Quadriceps Tendons | Clin Orthop Relat Res | Canada | A |
| 2022 | 35247574 | Focus on stability: biomechanical evaluation of external fixation technique versus locking plate osteosynthesis in 3-part proximal humeral fractures | J Shoulder Elbow Surg | Germany | A |
| 2022 | 35247577 | Biomechanical comparison of elbow stability constructs | J Shoulder Elbow Surg | USA | B |
| 2022 | 35254460 | Percutaneous fixation of intraarticular joint-depression calcaneal fractures with different screw configurations – a biomechanical human cadaveric analysis | Eur J Trauma Emerg Surg | Switzerland | C |
| 2022 | 35259657 | Use of digital imaging correlation techniques for full-field strain distribution analysis of implantable devices and tissue in spinal biomechanics research | J Biomech | USA | C |
| 2022 | 35265408 | Biomechanical Analysis of the Change of the Metatarsophalangeal Joint's Center of Rotation After Weil and Triple Weil Osteotomies: A Comparative Cadaveric Study | Adv Orthop | Greece | A |
| 2022 | 35265785 | Biomechanical Evaluation of Different Plate Configurations for Midshaft Clavicle Fracture Fixation Single Plating Compared with Dual Mini-Fragment Plating | JB JS Open Access | Canada | A |
| 2022 | 35280450 | Is there an increased risk for subtrochanteric stress fracture with the Femoral Neck System versus multiple cannulated screws fixation? | J Orthop | USA | B |
| 2022 | 35291241 | The Effects of Distal Pole Scaphoid Resection on Wrist Biomechanics | Arch Bone Jt Surg | USA | C |
| 2022 | 35293239 | Supination Adduction Vertical Medial Malleolar Fracture Fixation with Buttress Plating vs a Novel Screw-Only Construct: A Cadaveric Biomechanical Study | Foot Ankle Int | USA | C |
| 2022 | 35308050 | Patella Strength Characteristics in Cemented vs Press-fit Implants: A Biomechanical Analysis of Initial Stability | Arthroplast Today | USA | A |
| 2022 | 35309235 | Posterior Glenoid Osteotomy With Capsulolabral Repair Improves Resistance Forces in a Critical Glenoid Bone Loss Model | Orthop J Sports Med | USA | B |
| 2022 | 35321711 | Acromioclavicular joint instability on cross-body adduction view: the biomechanical effect of acromioclavicular and coracoclavicular ligaments sectioning | BMC Musculoskelet Disord | Japan | A |
| 2022 | 35323067 | Biomechanical Role of the Superior Capsule in a Rotator Cuff Sectioned and Repaired State A Sequential Sectioning Study | Am J Sports Med | USA | C |
| 2022 | 35337957 | Anterolateral Structure Reconstructions With Different Tibial Attachment Sites Similarly Improve Tibiofemoral Kinematics and Result in Different Graft Force in Treating Knee Anterolateral Instability | Arthroscopy | China | B |
| 2022 | 35340899 | Biomechanical Analysis of Coracoid Stability After Coracoplasty How Low Can You Go? | Orthop J Sports Med | Germany | A |
| 2022 | 35360977 | Residual stability of the distal radioulnar joint following ulnar styloid fracture: influence of the remnant distal radioulnar ligaments | J Hand Surg Eur Vol | Switzerland | B |
| 2022 | 35362777 | Biomechanics of hamstring tendon, quadriceps tendon, and bone–patellar tendon–bone grafts for anterior cruciate ligament reconstruction: a cadaveric study | Eur J Orthop Surg Traumatol | Canada | A |
| 2022 | 35364262 | Bankart Repair With Transferred Long Head of the Biceps Provides Better Biomechanical Effect Than Conjoined Tendon Transfer in Anterior Shoulder Instability With 20% Glenoid Defect | Arthroscopy | China | C |
| 2022 | 35364692 | Does augmentation increase the pull-out force of symphyseal screws? A biomechanical cadaver study | Eur J Trauma Emerg Surg | Germany | A |
| 2022 | 35369789 | Biomechanical Cadaveric Evaluation of the Role of Medial Column Instability in Hallux Valgus Deformity | Foot Ankle Int | Chile | C |
| 2022 | 35371655 | Validated Ossicular Measurements on High-Resolution Computed Tomography (CT) in Live and Cadaveric Temporal Bones | Cureus | India | A |
| 2022 | 35372302 | A Novel Adjustable EndoButton Fixation Assisted by 3D Printing Technology for Tibiofibular Syndesmosis Injury: A Biomechanical Study | Front Bioeng Biotechnol | China | A |
| 2022 | 35373636 | Lag screw osteosynthesis of simple olecranon fractures: A biomechanical comparative study | Proc Inst Mech Eng H | Germany | A |
| 2022 | 35379221 | Dynamic Tendon Grip (DTG™) novel knot array compared to traditional sutures for zone two flexor tendon injury – a biomechanical feasibility study | BMC Musculoskelet Disord | Israel | B |
| 2022 | 35381847 | Elongation of the brachial plexus after reverse shoulder arthroplasty: an anatomical study | Surg Radiol Anat | Spain | A |
| 2022 | 35386585 | Biomechanical Comparison of Krackow Repair and Percutaneous Achilles Repair System for Achilles Tendon Rupture Fixation: A Cadaveric and Finite Element Analysis Study | Foot Ankle Orthop | USA | B |
| 2022 | 35397657 | Biomechanical evaluation of interference screw fixation techniques for distal radioulnar ligament reconstruction: a cadaveric experimental study | Arch Orthop Trauma Surg | China | A |
| 2022 | 35398164 | Biomechanical comparison between various screw fixation angles for Latarjet procedure: a cadaveric biomechanical study | J Shoulder Elbow Surg | Taiwan | B |
| 2022 | 35399985 | Femur Strength is Similar Before and After Iatrogenic Fracture During Total Hip Arthroplasty: A Biomechanical Analysis | Arthroplast Today | USA | A |
| 2022 | 35400135 | Medial and Lateral Posterior Tibial Slope in the Skeletally Immature A Cadaveric Study | Orthop J Sports Med | USA | B |
| 2022 | 35400170 | Does tibial design modification improve implant stability for total knee arthroplasty? An experimental cadaver study | Bone Joint Res | Germany | A |
| 2022 | 35418662 | Biomechanical consequences of anterior root detachment of the lateral meniscus and its reinsertion | Sci Rep | Spain | A |
| 2022 | 35434767 | Cartilage decisively shapes the glenoid concavity and contributes significantly to shoulder stability | Knee Surg Sports Traumatol Arthrosc | Germany | B |
| 2022 | 35434856 | Biomechanical assessment of the first dorsal compartment of the wrist: A fresh cadaver study with relevance to de Quervain's disease | Clin Anat | Japan | A |
| 2022 | 35459614 | Biomechanical Comparison of Knotless Suture Anchor Versus Percutaneous End-to-End Technique for Mid-Substance Achilles Tendon Rupture Repair | J Foot Ankle Surg | USA | A |
| 2022 | 35464338 | Load Sharing in the Femur Using Strut Allografts: A Biomechanical Study | Arthroplast Today | USA | C |
| 2022 | 35466728 | Cadaveric Gait Simulation of the Effect of Subtalar Arthrodesis on Total Ankle Replacement Kinematics | Foot Ankle Int | USA | A |
| 2022 | 35467445 | Ankle joint pressure change before and after subtalar joint arthrodesis in varus and valgus malalignment of the tibia | J Orthop Surg (Hong Kong) | China | A |
| 2022 | 35482448 | Semitendinosus vs Gracilis Grafts With 1- vs 2-Tunnel Techniques for Coracoclavicular Ligament Reconstruction A Biomechanical Study | Am J Sports Med | USA | B |
| 2022 | 35485886 | Treatment of Medial Collateral Ligament Injury During Total Knee Arthroplasty With Internal Suture Brace Augmentation: A Cadaveric and Biomechanical Study | Orthopedics | USA | C |
| 2022 | 35489236 | Valgus fatigue and nonlinear damage accretion of the anterior bundle of the elbow medial collateral ligament (AMCL) | J Biomech | USA | A |
| 2022 | 35494267 | Endoscopic Gluteus Medius Repair Replicates Open, Knotless Repair With Similar Cyclic Loading Properties: A Cadaveric Study | Arthrosc Sports Med Rehabil | USA | C |
| 2022 | 35499547 | How to Optimize Pedicle Screw Parameters for the Thoracic Spine? A Biomechanical and Finite Element Method Study | Global Spine J | USA | B |
| 2022 | 35500811 | Biomechanical comparison of novel ulnar collateral ligament reconstruction with internal brace augmentation vs. modified docking technique | J Shoulder Elbow Surg | USA | B |

| | | | | | |
|---|---|---|---|---|---|
| 2022 | 35503457 | A Triple-Strand Anatomic Medial Collateral Ligament Reconstruction Restores Knee Stability More Completely Than a Double-Strand Reconstruction A Biomechanical Study In Vitro | Am J Sports Med | UK | A |
| 2022 | 35511430 | Medial Collateral Ligament Reconstruction for Anteromedial Instability of the Knee A Biomechanical Study In Vitro | Am J Sports Med | UK | A |
| 2022 | 35513813 | Biomechanical properties of a suture anchor system from human allogenic mineralized cortical bone matrix for rotator cuff repair | BMC Musculoskelet Disord | Austria | B |
| 2022 | 35532965 | Differences between Coracoclavicular, Acromioclavicular, or Combined Reconstruction Techniques on the Kinematics of the Shoulder Girdle | Am J Sports Med | Belgium | A |
| 2022 | 35534595 | Experimental validation of a voxel-based finite element model simulating femoroplasty of lytic lesions in the proximal femur | Sci Rep | Belgium | A |
| 2022 | 35535834 | Subtle segmental angle changes of single-level lumbar fusions and adjacent-level biomechanics: cadaveric study of optically measured disc strain | J Neurosurg Spine | USA | C |
| 2022 | 35576486 | Changes in the Tibial Strain After Proximal Fibular Osteotomy: A Biomechanical Cadaveric Study | Orthopedics | Malaysia | A |
| 2022 | 35578046 | Stapled fascial suture: ex vivo modeling and clinical implications | Surg Endosc | Italy | B |
| 2022 | 35579703 | Influence of rotator cuff preload on fracture configuration in proximal humerus fractures: a proof of concept for fracture simulation | Arch Orthop Trauma Surg | Germany | A |
| 2022 | 35583714 | Modified Lemaire tenodesis reduces anterior cruciate ligament graft forces during internal tibial torque loading | J Exp Orthop | Austria | A |
| 2022 | 35588289 | Biomechanical study of embracing and non-embracing rib plates | J Appl Biomater Funct Mater | China | A |
| 2022 | 35588587 | Malposition of components and Femorotibial mechanical Axis changes on pressure distribution in Total knee arthroplasty | Clin Biomech (Bristol, Avon) | China | C |
| 2022 | 35597534 | Biomechanical analysis of anterior stability after 15% glenoid bone loss: comparison of Bankart repair, dynamic anterior stabilization, dynamic anterior stabilization with Bankart repair, and Latarjet | J Shoulder Elbow Surg | USA | A |
| 2022 | 35597957 | A clinical and biomechanical comparison of INFIX plus single versus double sacroiliac screw fixation for unstable pelvic ring injury | J Orthop Surg Res | China | A |
| 2022 | 35598835 | Bicortical suspensory button fixation yields greater ultimate load to failure over unicortical all-suture anchor fixation in distal biceps brachii tendon repair | J Shoulder Elbow Surg | USA | B |
| 2022 | 35601735 | Biomechanical Testing of Scapular Spine Autograft for Anterior Glenoid Bone Augmentation | Orthop J Sports Med | USA | B |
| 2022 | 35603754 | Comparison of axial-rotational postoperative periprosthetic fracture of the femur in composite osteoporotic femur versus human cadaveric specimens: A validation study | Proc Inst Mech Eng H | UK | A |
| 2022 | 35604087 | A Biomechanical Comparison of the LaPrade Technique Versus a Novel Technique for Reconstruction of Medial-Sided Knee Injuries | Am J Sports Med | USA | B |
| 2022 | 35604117 | The Control of Anteromedial Rotatory Instability Is Improved With Combined Flat sMCL and Anteromedial Reconstruction | Am J Sports Med | Germany | C |
| 2022 | 35623287 | Region-specific associations among tissue-level mechanical properties, porosity, and composition in human male femora | J Biomech | USA | A |
| 2022 | 35635575 | The effect of long calcar screws on the primary stability of 3-part, varus impacted proximal humeral fractures compared to short calcar screws: a real fracture simulation study | Arch Orthop Trauma Surg | Germany | A |
| 2022 | 35643789 | Biomechanical Evaluation of 2 Common Approaches for Total Elbow Arthroplasty: Triceps Tongue Compared With Triceps Reflecting | J Hand Surg Am | USA | C |
| 2022 | 35647209 | Effect of Active and Passive Function of the Posterosuperior Rotator Cuff on Compensatory Muscle Loads in the Shoulder | Orthop J Sports Med | USA | B |
| 2022 | 35651480 | Infraspinatus Muscle Fiber Moment Arms During Abduction A Biomechanical Comparison of Values for Intact Rotator Cuff, Supraspinatus Tear, Superior Capsular Reconstruction, and Reverse Total Shoul | Orthop J Sports Med | USA | B |
| 2022 | 35660467 | Anatomical and radiological description of ligament insertions on the radial aspect of the scaphoid bone | Hand Surg Rehabil | Switzerland | A |
| 2022 | 35669699 | Kinematic and Radiographic Evaluation of Acromioclavicular Reconstruction with a Synthetic Ligament | Adv Orthop | USA | C |
| 2022 | 35671930 | Biomechanical comparison of lower trapezius and latissimus dorsi transfer for irreparable posterosuperior rotator cuff tears using a dynamic shoulder model | J Shoulder Elbow Surg | Germany | B |
| 2022 | 35671943 | Spondylophyte classification based on biomechanical effects on segmental stiffness | Spine J | Switzerland | A |
| 2022 | 35672888 | Biomechanical guidance can improve accuracy of reduction for intra-articular tibia plafond fractures and reduce joint contact stress | J Orthop Res | USA | C |
| 2022 | 35674265 | A Biomechanical Comparison of Gliding Resistance between Modified Lim Tsai and Asymmetric Tendon Repair Techniques in Zone II Flexor Tendon Repairs | J Hand Surg Asian Pac Vol | Singapore | B |
| 2022 | 35693457 | Effect of Sectioning of the Anterior Cruciate Ligament and Posterolateral Structures on Lateral Compartment Gapping A Randomized Biomechanical Study | Orthop J Sports Med | USA | B |
| 2022 | 35699755 | Discharging the medial knee compartment: comparison of pressure distribution and kinematic shifting after implantation of an extra-capsular absorber system (ATLAS) and open-wedge high tibial osteotomy | Arch Orthop Trauma Surg | Germany | A |
| 2022 | 35700368 | Impact of First Metatarsal Hyperpronation on First Ray Alignment: A Study in Cadavers | Clin Orthop Relat Res | USA | B |
| 2022 | 35701245 | Suture tension band fixation vs. metallic tension band wiring for patella fractures –A biomechanical study on 19 human cadaveric patellae | Injury | Denmark | A |
| 2022 | 35722810 | An Increased Allograft Width for Circumferential Labral Reconstruction Better Restores Distractive Stability of the Hip A Cadaveric Biomechanical Analysis | Am J Sports Med | USA | B |
| 2022 | 35722811 | Biomechanical Comparison of Ulnar Collateral Ligament Reconstruction With and Without Suture Augmentation | Am J Sports Med | USA | B |
| 2022 | 35730534 | Radiographic and Cadaveric Analysis of Minimally Invasive Bunionectomy Osteotomy Position—"MIS Bunion Sweet Spot" | Foot Ankle Spec | USA | B |
| 2022 | 35734768 | Ability to Retension Knotless Suture Anchors A Biomechanical Analysis of Simulated Bankart Lesions | Orthop J Sports Med | USA | B |
| 2022 | 35735256 | Cementless femoral stem revision in total hip arthroplasty: The periprosthetic clamshell fracture. A biomechanical investigation | J Orthop Res | Austria | C |
| 2022 | 35747626 | Autologous Fibrin Sealants Have Comparable Graft Fixation to an Allogeneic Sealant in a Biomechanical Cadaveric Model of Chondral Defect | Arthrosc Sports Med Rehabil | USA | A |
| 2022 | 35747631 | Biomechanical Comparison of Three Suspensory Techniques for all Soft Tissue Central Quadriceps Tendon Graft Fixation | Arthrosc Sports Med Rehabil | Canada | A |
| 2022 | 35753638 | Height restoration and sustainability using bilateral vertebral augmentation systems for vertebral compression fractures: a cadaveric study | Spine J | USA | A |
| 2022 | 35757238 | Biomechanical Evaluation of 4 Suture Techniques for Hip Capsular Closure | Orthop J Sports Med | USA | B |
| 2022 | 35759107 | Biomechanical comparison of a novel tensioned cable construct versus tension band wiring for transverse patella fracture fixation | Eur J Orthop Surg Traumatol | USA | C |
| 2022 | 35772603 | Concomitant Meniscotibial Ligament Reconstruction Decreases Meniscal Extrusion Following Medial Meniscus Allograft Transplantation: A Cadaveric Analysis | Arthroscopy | USA | B |
| 2022 | 35776928 | Pull-out Strength of Suture Anchor and Torque of Buddy Anchor for an Osteoporotic Humeral Head in Rotator Cuff Repair Parallel Versus Divergent Insertion | Am J Sports Med | Korea | B |
| 2022 | 35777678 | Modified Lemaire Lateral Extra-articular Tenodesis With the Iliotibial Band Strip Fixed on the Femoral Cortical Surface Reduces Laxity and Causes Less Overconstraint in the Anterolateral Lesioned Knee: . | Arthroscopy | China | B |
| 2022 | 35778231 | All-Suture Anchor Repair of the Flexor Digitorum Profundus Insertion: A Biomechanical Comparison of 2 Suturing Techniques | J Hand Surg Am | USA | C |
| 2022 | 35782774 | A Biomechanical Analysis of Shoulder Muscle Excursions During Abduction, After the Treatment of Massive Irreparable Rotator Cuff Tears Using Superior Capsular Reconstruction (SCR), Bursal Acromial | J Shoulder Elb Arthroplast | USA | B |
| 2022 | 35787211 | Primary Fixation and Cyclic Performance of Single-Stitch All-Inside and Inside-Out Meniscal Devices for Repairing Vertical Longitudinal Meniscal Tears | Am J Sports Med | Germany | B |
| 2022 | 35803782 | Fracture Fixation Strength in Metacarpal Plating Versus Intramedullary Nailing Using a 3-Point Bending Model: A Cadaveric, Biomechanical Study | J Hand Surg Am | USA | C |
| 2022 | 35810043 | Comminuted intraarticular calcaneal fractures: Multiplanar VA locked plating and interlocked nailing incorporate longitudinal strut and provide superior stability –a biomechanical cadaveric study | Injury | Switzerland | C |
| 2022 | 35815624 | Conduit/Wrap Repairs to Digital Nerves Provide Residual Strength After Peak Loading | Hand (N Y) | USA | A |
| 2022 | 35817317 | Divergent mechanical properties of older human male femora reveal unique combinations of morphological and compositional traits contributing to low strength | Bone | USA | B |
| 2022 | 35831608 | Tape suture constructs for instabilities of the pubic symphysis: is the idea of motion preservation a suitable treatment option? A cadaver study | Arch Orthop Trauma Surg | Germany | A |
| 2022 | 35834709 | In Situ mechanical effects of a specific neurodynamic mobilization of the superficial fibular nerve: a cadaveric study | J Athl Train | Canada | A |
| 2022 | 35834895 | In vitro method to quantify and visualize mechanical wear in human meniscus subjected to joint loading | J Mech Behav Biomed Mater | USA | C |
| 2022 | 35837435 | C1-C2 arthroplasty for craniovertebral junction instability: A preliminary proof of concept in human cadavers | J Craniovertebr Junction Spine | India | C |
| 2022 | 35843762 | Optimizing Volar Tilt Restoration and Plate Position in Distal Radius Fractures | J Hand Surg Am | USA | C |
| 2022 | 35844971 | A Novel Calcium Phosphate–Based Nanocomposite for Augmentation of Cortical Bone Trajectory Screw Fixation | Int J Nanomedicine | China | A |
| 2022 | 35850519 | Biomechanical Testing of a Novel Device for Sutureless Nerve Repair | Tissue Eng Part C Methods | USA | B |
| 2022 | 35868900 | Articular Involvement with Retrograde Headless Compression Screw Fixation of the Metacarpal | J Hand Surg Am | USA | B |
| 2022 | 35869276 | Evaluation of the contact surface between vertebral endplate and 3D printed patient-specific cage vs commercial cage | Sci Rep | Canada | A |
| 2022 | 35870957 | A Biomechanical Analysis of the H-Taping Method Used by Rock Climbers as Prophylactic or Stabilizing Fixation of Partial A2 Pulley Tears | J Hand Surg Am | USA | C |

| | | | | | |
|---|---|---|---|---|---|
| 2022 | 35871315 | Biomechanical effect of fibular osteotomy on the knee joint in high tibial osteotomy: A cadaveric study | Biomed Mater Eng | Japan | A |
| 2022 | 35871853 | Biomechanical comparison of the new cross-locking intramedullary nail with tension band wiring for transverse olecranon fractures | Injury | China | B |
| 2022 | 35872128 | Influence of Spinal Deformity Construct Design on Adjacent-Segment Biomechanics | World Neurosurg | USA | C |
| 2022 | 35875489 | Two Cannulated Screws Provide Sufficient Biomechanical Strength for Prophylactic Fixation in Adult Patients With an Aggressive Benign Femoral Neck Lesion | Front Bioeng Biotechnol | China | B |
| 2022 | 35895089 | Rotator cuff tear reaching the superior half portion of the humeral head causes shoulder abduction malfunction | Knee Surg Sports Traumatol Arthrosc | China | B |
| 2022 | 35901707 | Biomechanical assessment of the effect of sublaminar band tensioning on lumbar motion | J Neurosurg Spine | USA | B |
| 2022 | 35907280 | First detection of primary cilia in injured human anterior cruciate ligament: A pilot study with pathophysiological reflections | Pathol Res Pract | Germany | A |
| 2022 | 35908472 | Grip strength after hamate hook excision and reconstruction surgery: A biomechanical cadaveric study | J Biomech | Thailand | A |
| 2022 | 35923441 | Biomechanical investigation of the hybrid modified cortical bone screw–pedicle screw fixation technique: Finiteelement analysis | Front Surg | China | A |
| 2022 | 35944289 | Validation of dynamic biplane radiography and three-dimensional model-based tracking for evaluation of dynamic thumb kinematics | J Biomech | USA | A |
| 2022 | 35946420 | Simultaneous Measurement of Patellofemoral Joint Kinematics and Contact Mechanics in Intact Knees: A Cadaveric Study | Orthop Surg | China | A |
| 2022 | 35947455 | Biomechanical Strength of Screw Versus Suture Button Fixation in the Latarjet Procedure: A Cadaver Study | Orthopedics | USA | A |
| 2022 | 35951242 | Surgical techniques in restoration lumbar lordosis: a biomechanical human cadaveric study | Spine Deform | The Netherlands | B |
| 2022 | 35958291 | Ultrasound-Guided Anterior Talofibular Ligament Repair With Augmentation Can Restore Ankle Kinematics: A Cadaveric Biomechanical Study | Orthop J Sports Med | USA | A |
| 2022 | 35964832 | Biomechanical considerations of the posterior surgical approach to the lumbar spine | Spine J | Switzerland | A |
| 2022 | 35971598 | Biomechanical investigation of arm position on deforming muscular forces in proximal humerus fractures | Clin Shoulder Elb | USA | B |
| 2022 | 35982831 | Biomechanical Comparison of a Novel Multiplanar, Perpendicular Whipstitch With the Krackow Stitch and Standard Commercial Whipstitch | Orthop J Sports Med | USA | A |
| 2022 | 35988793 | Superior Capsule Reconstruction With Fascia Lata Allograft Has Initial Stiffness and Ultimate Load Comparable to the Native Shoulder Superior Capsule: A Cadaveric Biomechanical Study | Arthroscopy | USA | C |
| 2022 | 35993448 | Biomechanical Comparison of Fiber Tape Device Versus Transarticular Screws for Ligamentous Lisfranc Injury in a Cadaveric Model | Am J Sports Med | USA | B |
| 2022 | 35996543 | Biomechanical Characterization of Unilateral and Bilateral Posterior Lumbar Interbody Fusion Constructs | Biomed Res Int | China | C |
| 2022 | 36007772 | C1 Lateral Mass Screw Placement Through Endonasal Corridor for Purpose of O-C1 Fusion: Morphometric Analysis in Cadaveric Specimens | World Neurosurg | USA | C |
| 2022 | 36007955 | Mechanical and Geometric Analysis of Fenestration Design for Polymethylmethacrylate-Augmented Pedicle Screw Fixation | Int J Spine Surg | Australia | A |
| 2022 | 36018371 | Open reduction internal fixation of rib fractures: a biomechanical comparison between the RibLoc U Plus ® system and anterior plate in rib implants | Eur J Trauma Emerg Surg | USA | C |
| 2022 | 36028376 | Biomechanical analysis of dual versus lateral locked plating in elderly bicondylar tibial plateau fractures: Does medial comminution matter? | Injury | USA | B |
| 2022 | 36029265 | Adding sacral anchors through an S1 alar screw and multirod construct as a strategy for lumbosacral junction augmentation: an in vitro comparison to S1 pedicle screws alone with sacroiliac fixation | J Neurosurg Spine | China | C |
| 2022 | 36033186 | Anterior to Posterior Bone Plug Suture Tunnels Provide Optimal Biomechanics for Bone[illegible]Patellar Tendon[illegible]Bone Anterior Cruciate Ligament Graft | Arthrosc Sports Med Rehabil | USA | B |
| 2022 | 36036524 | Two Orthogonal Nitinol Staples and Combined Nitinol Staple-Screw Constructs for a First Metatarsophalangeal Joint Arthrodesis: A Biomechanical Cadaver Study | Foot Ankle Int | USA | B |
| 2022 | 36046758 | The effect of different insert design congruencies on the kinematics of a mobile bearing TKA: A cadaveric study | J Orthop | Belgium | A |
| 2022 | 36049770 | Effect of Poly-L-Lactic Acid Mesh Augmentation on Cyclic Gap Formation in Transosseous Patellar Tendon Repair: A Biomechanical Study | J Knee Surg | USA | B |
| 2022 | 36050989 | Tracheal reconstruction: mini-review and anatomical study on the use of antero lateral thigh fascial flap for heterotopical transplantation | Indian J Thorac Cardiovasc Surg | Italy | B |
| 2022 | 36054510 | Effects of Chemical Sterilization and Gamma Irradiation on the Biochemical and Biomechanical Properties of Human Tendon Allografts In Vitro Study | Orthop Surg | China | B |
| 2022 | 36057488 | Biomechanical comparison of tuberosity-based proximal humeral locking plate compared to standard proximal humeral locking plate in varus cantilever bending | Injury | USA | C |
| 2022 | 36061438 | Biomechanical comparative analysis of effects of dynamic and rigid fusion on lumbar motion with different sagittal parameters: An in vitro study | Front Bioeng Biotechnol | China | A |
| 2022 | 36071256 | An anatomically shaped medial meniscus prosthesis is able to partially restore the contact mechanics of the meniscectomized knee joint | J Exp Orthop | The Netherlands | A |
| 2022 | 36071928 | An In vitro study of biomechanical comparison between titanium 4-hole 3D plates and titanium conventional 4-hole miniplates for internal fixation of mandibular angle fractures | J Oral Biol Craniofac Res | India | A |
| 2022 | 36079163 | Biomechanical Study of Arthroscopic All-Inside Anterior Talofibular Ligament Suture Augmentation Repair, Plus Suture Augmentation Repair and Anterior Tibiofibular Ligament's Distal Fascicle Transfer | J Clin Med | China | A |
| 2022 | 36083835 | Biomechanics of Transforaminal Endoscopic Approaches | Spine (Phila Pa 1976) | Switzerland | A |
| 2022 | 36089992 | Biomechanical analysis of stability of joint depression calcaneal fractures: Fixation with locking compression plate alone compared with addition of supplemental oblique screw | J Clin Orthop Trauma | USA | C |
| 2022 | 36104991 | Biomechanics comparison between endobutton fixation and syndesmotic screw fixation for syndesmotic injury ankle fracture; a finite element analysis and cadaveric validation study | J Orthop | Thailand | B |
| 2022 | 36108881 | Biomechanical evaluation of subscapularis peel repairs augmented with the long head of the biceps tendon for anatomic total shoulder arthroplasty | J Shoulder Elbow Surg | USA | B |
| 2022 | 36113193 | Relooking at double-bundle versus single-bundle anterior cruciate ligament reconstruction: A biomechanical model to evaluate which can confer better rotatory stability | Clin Biomech (Bristol, Avon) | Singapore | B |
| 2022 | 36119121 | Effect of Anterior Horn Tears of the Lateral Meniscus on Knee Stability | Orthop J Sports Med | Japan | A |
| 2022 | 36119124 | Comparison of Treatment Methods for Syndesmotic Injuries With Posterior Tibiofibular Ligament Ruptures A Cadaveric Biomechanical Study | Orthop J Sports Med | Japan | A |
| 2022 | 36163679 | Patient-Specific Drill Guide Template for Pedicle Screw Insertion into the Atlantoaxial Cervical Spine Using Stereolithographic Modeling: An In Vitro Study | Asian Spine J | Philippines | A |
| 2022 | 36167289 | The role of the lateral part of the distal triceps and the anconeus in varus stability of the elbow: a biomechanical study | J Shoulder Elbow Surg | USA | A |
| 2022 | 36167986 | Biomechanical comparison of screw vs. cerclage refixation in orthogeriatric lesser trochanteric fractures: a cadaveric study | Eur J Trauma Emerg Surg | Germany | A |
| 2022 | 36182593 | A biomechanical comparison of superior ramus plating versus intramedullary screw fixation for unstable lateral compression pelvic ring injuries | Injury | USA | C |
| 2022 | 36183919 | Shoulder Anteroinferior Glenoid Labrum Reconstruction With the Long Head of the Biceps Tendon Restores Glenohumeral Stability: A Cadaveric Biomechanical Study | Arthroscopy | USA | C |
| 2022 | 36185347 | Displacement of the Metatarsal Sesamoids in Relation to First Metatarsophalangeal Joint Extension | Foot Ankle Orthop | USA | B |
| 2022 | 36186708 | No Difference in Knee Kinematics Between Anterior Cruciate Ligament–First and Posterior Cruciate Ligament–First Fixation During Single-Stage Multiligament Knee Reconstruction | Orthop J Sports Med | USA | A |
| 2022 | 36186709 | The Role of the Trapezius in Stabilization of the Acromioclavicular Joint A Biomechanical Evaluation | Orthop J Sports Med | USA | B |
| 2022 | 36190171 | Morphometric and Biomechanical Comparison of the Scapular Spine and Coracoid Graft in Anterior Shoulder Instability | Am J Sports Med | Taiwan | B |
| 2022 | 36191732 | Medial Meniscus Posterior Root Repair Restores Contact Pressure and Contact Area to Its Native State Even After Opening-Wedge High Tibial Osteotomy: A Cadaveric Biomechanical Study | Arthroscopy | Korea | B |
| 2022 | 36195356 | Accuracy and precision of the measurement of liner orientation of dual mobility cup total hip arthroplasty using ultrasound imaging | Med Eng Phys | France | B |
| 2022 | 36195367 | Mechanical testing and biomechanical CT analysis to assess vertebral flexion strength of Chinese cadavers | Med Eng Phys | China | A |
| 2022 | 36197491 | Screw placement in arthroscopically assisted osteosynthesis of radial head fractures using a reference k-wire in the radiocapitellar joint: a cadaveric study | Arch Orthop Trauma Surg | Germany | A |
| 2022 | 36203305 | Biomechanical Effects of Proximal Polyetheretherketone Rod Extension on the Upper Instrumented and Adjacent Levels in a Human Long-Segment Construct: A Cadaveric Model | Neurospine | USA | B |
| 2022 | 36204515 | Biomechanical comparison of continuous compression implants versus tension band fixation for transverse olecranon fractures | J Orthop | USA | A |
| 2022 | 36205307 | Negative Ulnar Variance Lessens DRUJ Instability After DRUJ Disruption: A Biomechanical Analysis | Hand (N Y) | USA | B |
| 2022 | 36206982 | Functional biomechanical comparison of Latarjet vs. distal tibial osteochondral allograft for anterior glenoid defect reconstruction | J Shoulder Elbow Surg | USA | A |
| 2022 | 36207344 | Dynamic pressure analysis of novel interpositional knee spacer implants in 3D-printed human knee models | Sci Rep | Germany | A |
| 2022 | 36215262 | Mechanisms of cervical spine injury and coupling response with initial head rotated posture - implications for AIS coding | Traffic Inj Prev | USA | A |
| 2022 | 36228418 | Biomechanical comparison of two InternalBrace™ reinforced thumb ulnar collateral ligament reconstruction techniques | Clin Biomech (Bristol, Avon) | Norway | B |
| 2022 | 36229309 | Effect of Radiocapitellar Joint Over/Under Stuffing on Elbow Joint Contact Pressure | J Hand Surg Am | USA | A |

| | | | | | |
|---|---|---|---|---|---|
| 2022 | 36237220 | Relationship between the progression of posterosuperior rotator cuff tear size and shoulder abduction function: A cadaveric study via dynamic shoulder simulator | Front Bioeng Biotechnol | China | B |
| 2022 | 36239404 | Kinematic Analysis of Sequential PartialMidfoot Arthrodesis in Simulated Gait Cadaver Model | Foot Ankle Int | USA | A |
| 2022 | 36243298 | Subject-specific computational modeling of acromioclavicular and coracoclavicular ligaments | J Shoulder Elbow Surg | USA | B |
| 2022 | 36252309 | ACL transection results in a posterior shift and increased velocity of contact on the medial tibial plateau | J Biomech | USA | A |
| 2022 | 36252785 | Impact of radiocapitellar interposition arthroplasty on ulnohumeral joint biomechanics | J Shoulder Elbow Surg | USA | B |
| 2022 | 36259252 | Biomechanical Comparison of Subsidence Between Patient-Specific and Non-Patient-Specific Lumbar Interbody Fusion Cages | Global Spine J | Canada | A |
| 2022 | 36259688 | The Foot and Ankle Kinematics of a Simulated Progressive Collapsing Foot Deformity During Stance Phase: A Cadaveric Study | Foot Ankle Int | USA | A |
| 2022 | 36283563 | Stability, Deformity and Fixation of the Floating Shoulder: A Cadaveric Biomechanical Study | J Shoulder Elbow Surg | USA | A |
| 2022 | 36283564 | Evaluation of Using Electrocautery Devices for Suture Passage through Greater Tuberosity: A Biomechanical Study | J Shoulder Elbow Surg | USA | A |
| 2022 | 36289037 | The consequences of a thoracic outlet syndrome's entrapment model on the biomechanics of the ulnar nerve - Cadaveric study | J Hand Ther | France | C |
| 2022 | 36290532 | Restoration of the Joint Line Configuration Reproduces Native Mid-Flexion Biomechanics after Total Knee Arthroplasty: A Matched-Pair Cadaveric Study | Bioengineering (Basel) | Korea | A |
| 2022 | 36295579 | Impact of Capsulotomy on Hip Biomechanics during Arthroscopy | Medicina (Kaunas) | Korea | A |
| 2022 | 36305761 | Biomechanical Analysis of Posterior Open-Wedge Osteotomy and Glenoid Concavity Reconstruction Using an Implant-Free, J-Shaped Iliac Crest Bone Graft | Am J Sports Med | Switzerland | A |
| 2022 | 36307286 | The Evaluation of a Flexor Digitorum Profundus-to-Volar Plate Zone I Repair Versus Button Repair: An In vitro Biomechanics Study | J Hand Surg Am | USA | C |
| 2022 | 36312697 | Three Medial All Suture Anchors Improves Contact Force Compared to Two Hard Body Anchors in a Biomechanical Two-Tendon Rotator Cuff Tear Model | Arthrosc Sports Med Rehabil | USA | B |
| 2022 | 36312720 | Five-Strand Hamstring Grafts are Biomechanically Comparable to Four-Strand Grafts and Offer Greater Diameter for Anterior Cruciate Ligament Reconstruction | Arthrosc Sports Med Rehabil | USA | B |
| 2022 | 36313006 | Cadaveric Biomechanical Evaluation of Capsular Constraint and Microinstability After Hip Capsulotomy and Repair | Orthop J Sports Med | Canada | A |
| 2022 | 36321602 | Biomechanical Comparison of a Novel 3-Screw Fixation vs Conventional 2-Screw Fixation of Calcaneal Tuberosity Avulsion Fractures | Foot Ankle Int | Singapore | A |
| 2022 | 36328339 | Biomechanical Comparison of 3Medial Patellofemoral Complex Reconstruction Techniques Shows Medial Overconstraint but no Significant Difference in Patella Lateralization and Contact Pressure | Arthroscopy | USA | A |
| 2022 | 36331129 | A Transosseous Suture as an Alternative to Suture Anchor on Anterior-Avulsion Greater Tuberosity Fragment Fixation in Neer Three-Part Proximal Humeral Fracture: A Biomechanical Study | Orthop Surg | China | B |
| 2022 | 36333241 | First Carpometacarpal Joint Motion and Proximal Migration of the First Metacarpal After Tensioning of a Suture Device Suspensionplasty Compared With Trapeziectomy: A Biomechanical Cadaver Study | J Hand Surg Am | USA | C |
| 2022 | 36343768 | Superior Capsule Reconstruction Using Acellular Dermal Allograft Secured at 45° of Glenohumeral Abduction Improves the Superior Stability of the Glenohumeral Joint in Irreparable Massive Posterosuper | Arthroscopy | USA | B |
| 2022 | 36348287 | Reproducibility and repeatability of a semi-automated pipeline to quantify trapeziometacarpal joint angles using dynamic computed tomography | BMC Med Imaging | Canada | A |
| 2022 | 36353428 | Effect of glenosphere lateralization with and without coracoacromial ligament transection on acromial and scapular spine strain in reverse shoulder arthroplasty | JSES Int | USA | B |
| 2022 | 36389621 | The Role of the Medial Meniscus in Anterior Knee Stability | Orthop J Sports Med | Japan | A |
| 2022 | 36395965 | Long Head of the Biceps Autograft Performs Biomechanically Similar to Human Dermal Allograft for Superior Capsule Reconstruction after Rotator Cuff Tear | Arthroscopy | USA | B |
| 2022 | 36402286 | Anatomy of the biceps brachii osseous footprint: study of 100 radii and literature review | Hand Surg Rehabil | France | A |
| 2022 | 36403528 | The loads developed by epicondylar and epitrochlear muscles across the elbow joint. A dynamic simulated model | J Biomech | Spain | C |
| 2022 | 36405545 | Intersurgeon Consistency of Ulnar Collateral Ligament Repair With Internal Brace A Biomechanical Analysis | Orthop J Sports Med | USA | B |
| 2022 | 36412519 | Biomechanical Effect of Differential Tensioning on Suture-Augmented Ulnar Collateral Ligament Reconstruction of the Elbow | Am J Sports Med | USA | C |
| 2022 | 36412536 | Biomechanical Efficacy of Tape Cerclage as an Augment or Stand-alone for Coracoclavicular Ligament Reconstruction | Am J Sports Med | USA | B |
| 2022 | 36419474 | Small Chondral Defects Affect Tibiofemoral Contact Area and Stress Should a Lower Threshold Be Used for Intervention? | Orthop J Sports Med | USA | B |
| 2022 | 36419477 | Comparing the Anatomical Landmarks Versus the Coracoid-Based Landmarks Techniques for Coracoclavicular Stabilization After High-Grade Acromioclavicular Injury A Biomechanical Study | Orthop J Sports Med | Thailand | A |
| 2022 | 36422175 | Impact of Anterior Malposition and Bone Cement Augmentation on the Fixation Strength of Cephalic Intramedullary Nail Head Elements | Medicina (Kaunas) | Switzerland | A |
| 2022 | 36425092 | The anterior head of deltoid in relation to anterior clavicle plating: how much are we releasing? | OTA Int | USA | C |
| 2022 | 36425884 | Biomechanical tests and finite element analyses of pelvic stability using bilateral single iliac screws with different channels in lumbo-iliac fixation | Front Surg | China | A |
| 2022 | 36439368 | Role of the posterior deep deltoid ligament in ankle fracture stability: A biomechanical cadaver study | World J Orthop | UK | A |
| 2022 | 36451236 | Cerclage performance analysis – a biomechanical comparison of different techniques and materials | BMC Musculoskelet Disord | Germany | A |
| 2022 | 36453729 | Anteromedialization Tibial Tubercle Osteotomy Improves Patellar Contact Forces A Cadaveric Model of Patellofemoral Dysplasia | Am J Sports Med | USA | B |
| 2022 | 36461826 | Anterior atlanto-occipital transarticular screw fixation: a biomechanical comparison with posterior fixation techniques | J Neurosurg Spine | China | C |
| 2022 | 36463220 | Transosseous suture versus suture anchor fixation for inferior pole fractures of the patella in osteoporotic bone: a biomechanical study | Eur J Med Res | Germany | A |
| 2022 | 36493692 | How to improve the biomechanical stability of endosteal augmentation for proximal humerus fracture with osteopenia? A cadaveric study | Clin Biomech (Bristol, Avon) | China | A |
| 2022 | 36508022 | The ideal site of cement application in cement augmented sacroiliac screw fixation: the biomechanical perspective | Eur J Trauma Emerg Surg | Switzerland | B |
| 2022 | 36512058 | The simulation of terrible triad injuries in fresh-frozen human cadaveric specimens with intact soft tissue envelope | Arch Orthop Trauma Surg | Germany | A |
| 2022 | 36517927 | Intratendinous pressure changes in the Achilles tendon during stretching and eccentric loading: Implications for Achilles tendinopathy | Scand J Med Sci Sports | Belgium | A |
| 2022 | 36518934 | Biomechanical Analysis of Three Different Reconstruction Techniques for Scapholunate Instability: A Cadaveric Study | Clin Orthop Surg | Korea | C |
| 2022 | 36529382 | Biomechanical Comparison of Combined Latissimus Dorsi and Teres Major Tendon Transfer versus Latissimus Dorsi Tendon Transfer in Shoulders with Irreparable Anterosuperior Rotator Cuff Tears | J Shoulder Elbow Surg | USA | B |
| 2022 | 36532146 | Measurement of the Balance Stability Angle to Predict the Stability Ratio in Patients With Recurrent Anterior Shoulder Dislocation A Novel Computed Tomography–Based Protocol | Orthop J Sports Med | China | A |
| 2022 | 36535559 | Biomechanical analysis of plating techniques for unstable lateral clavicle fractures with coracoclavicular ligament disruption (Neer Type-IIB) | J Shoulder Elbow Surg | Australia | A |
| 2022 | 36541011 | Anatomic and Biomechanical Study of Thumb Carpometacarpal Dislocations: A Laboratory Study | Hand (N Y) | USA | A |
| 2022 | 36545379 | A Biomechanical Comparison of 2 Over-the-Top Anterior Cruciate Ligament Reconstruction Techniques A Cadaveric Study Using a Robotic Simulator | Orthop J Sports Med | Japan | A |
| 2022 | 36550296 | Development and preclinical evaluation of a cable-clamp fixation device for a disrupted pubic symphysis | Commun Med (Lond) | Germany | B |
| 2022 | 36570209 | Bionate Lumbar Disc Nucleus Prosthesis: Biomechanical Studies in Cadaveric Human Spines | ACS Omega | Spain | B |
| 2022 | 36579044 | Transtibial Repair of Lateral Meniscus Posterior Root Tears Improves Contact Biomechanics in Pediatric Cadavers | Arthrosc Sports Med Rehabil | USA | B |
| 2022 | 36579049 | All-Suture Suspensory Button Has Similar Biomechanical Performance to Metal Suspensory Button for Onlay Subpectoral Biceps Tenodesis | Arthrosc Sports Med Rehabil | USA | A |
| 2022 | 36579055 | Single- and Double-Loaded All-Suture Anchor Repairs of Anteroinferior Labral Tears Are Biomechanically Similar in a Cadaveric Shoulder Model | Arthrosc Sports Med Rehabil | USA | A |
| 2022 | 36583232 | Biomechanical Comparison of Unilateral and Bilateral Pedicle Screw Fixation after Multilevel Lumbar Lateral Interbody Fusion | Global Spine J | USA | C |
| 2022 | 36584578 | Is the rod necessary? Biomechanical comparison of static knee spacers during axial loading | Clin Biomech (Bristol, Avon) | Germany | A |
| 2022 | 36587893 | A novel pedicle screw design with variable thread geometry: Biomechanical cadaveric study with finite element analysis | World Neurosurg | India | C |
| 2022 | 36637310 | Cisternal, Falciform, and Optic Canal Decompression Influencing Optic Nerve Biomechanics: A Microsurgical Anatomic Study | Oper Neurosurg (Hagerstown) | USA | B |
