# Supplementary material for: Global trends and insights in ethical statements regarding the utilization of human cadaveric tissues for biomechanical research from 2017 to 2022: a bibliometric analysis
Source: Int J Surg. 2024 Jul 24;111(1):1503–7. doi: 10.1097/JS9.0000000000001986 (PMC11745705; doi:10.1097/JS9.0000000000001986)

**Figure S1: Trends in percentage of grade B at the regional level between 2017 and 2022.**

**
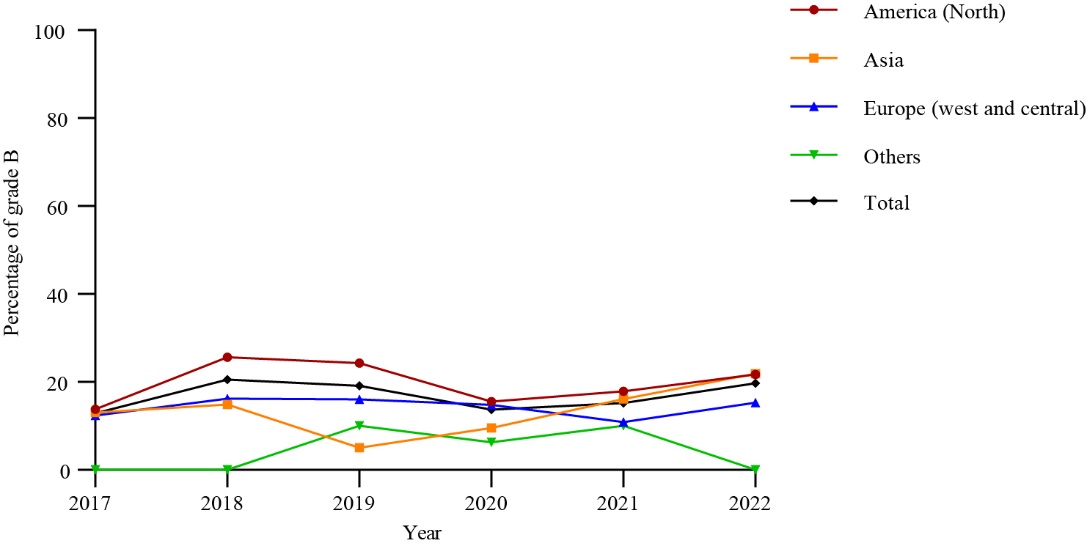
**

**Figure S2: Trends in percentage of grade C at the regional level between 2017 and 2022.**

**
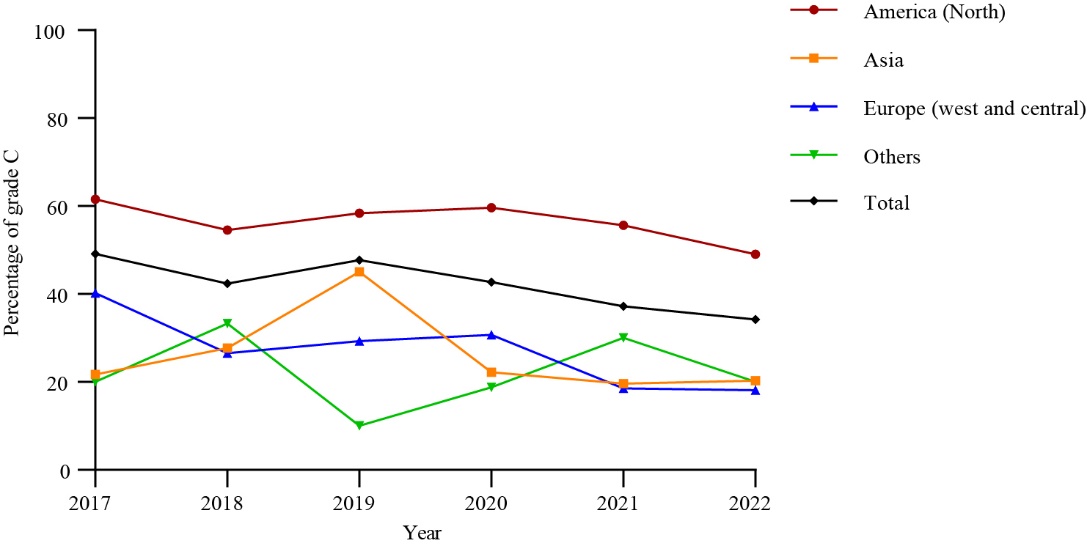
**

**Figure S3: Plot of percentage of grade B of individual country or regions (published more than 5 articles) by Human Development Index (HDI).**

**
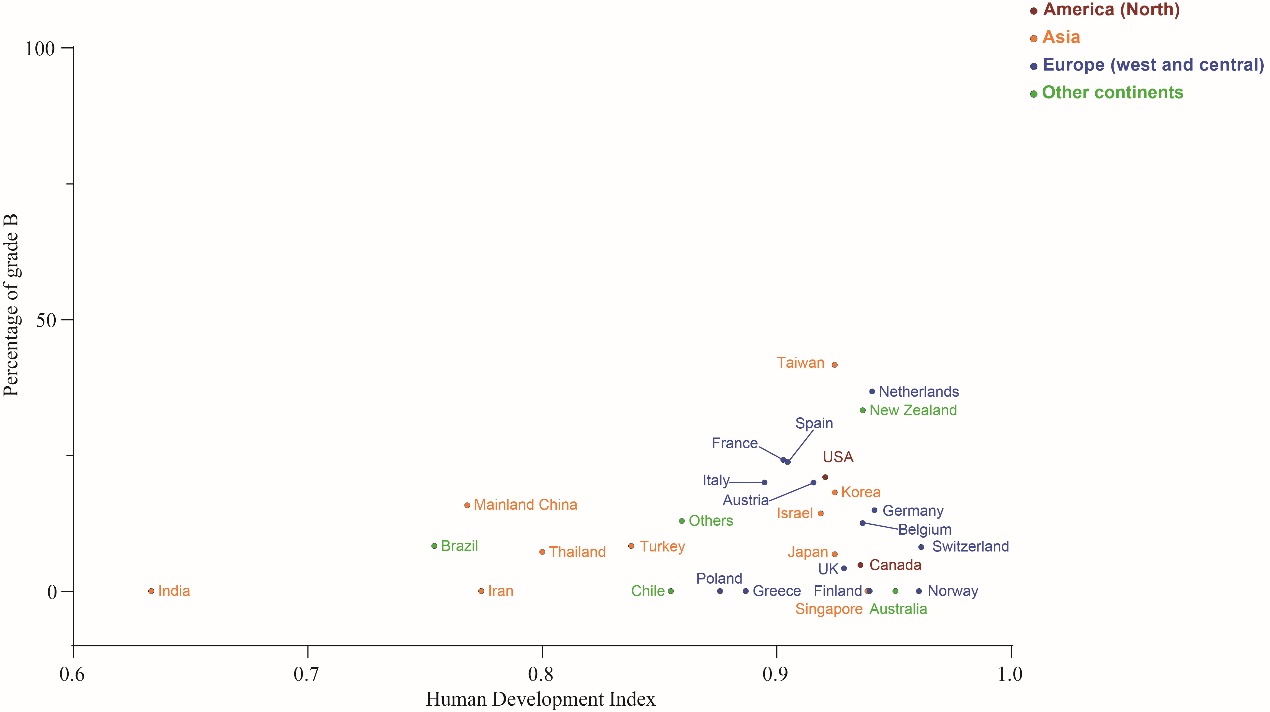
**

**Figure S4: Plot of percentage of grade C of individual country or regions (published more than 5 articles) by Human Development Index (HDI).**

**
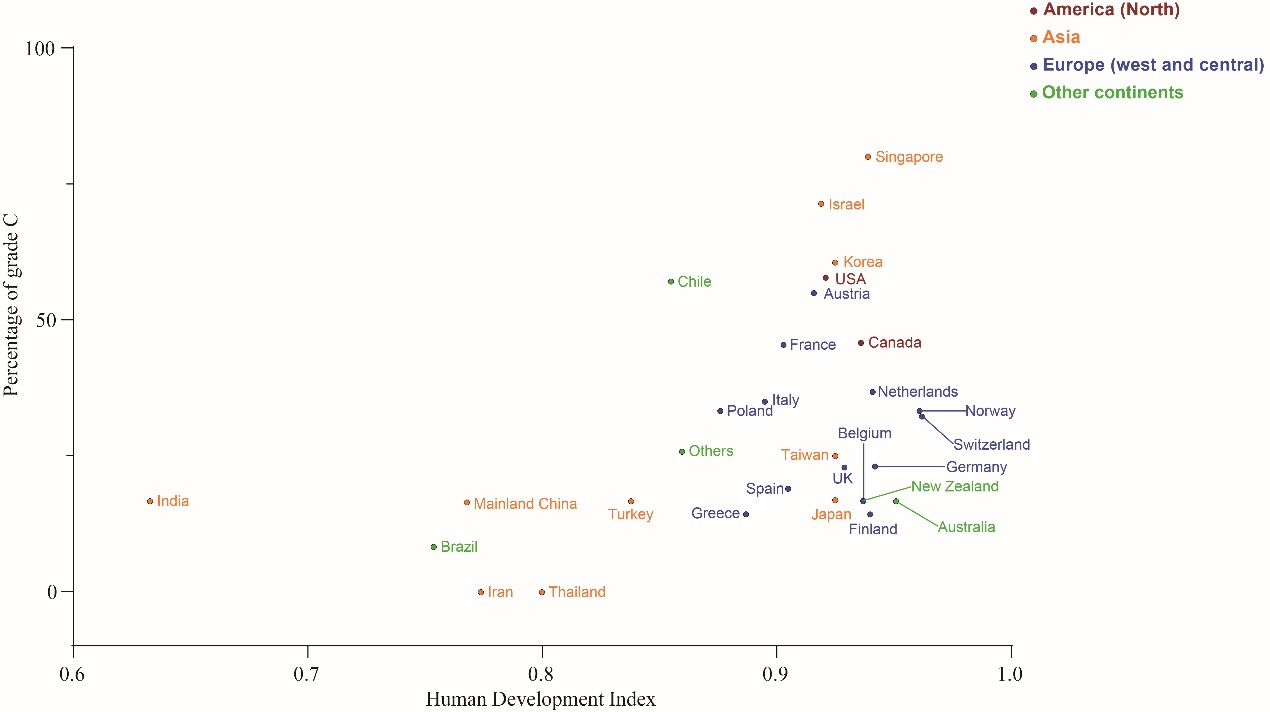
**

**Figure S5: Plot of percentage of grade B of individual country or regions (published more than 5 articles) by Gross Domestic Product (GDP) per capita in US$.**

**
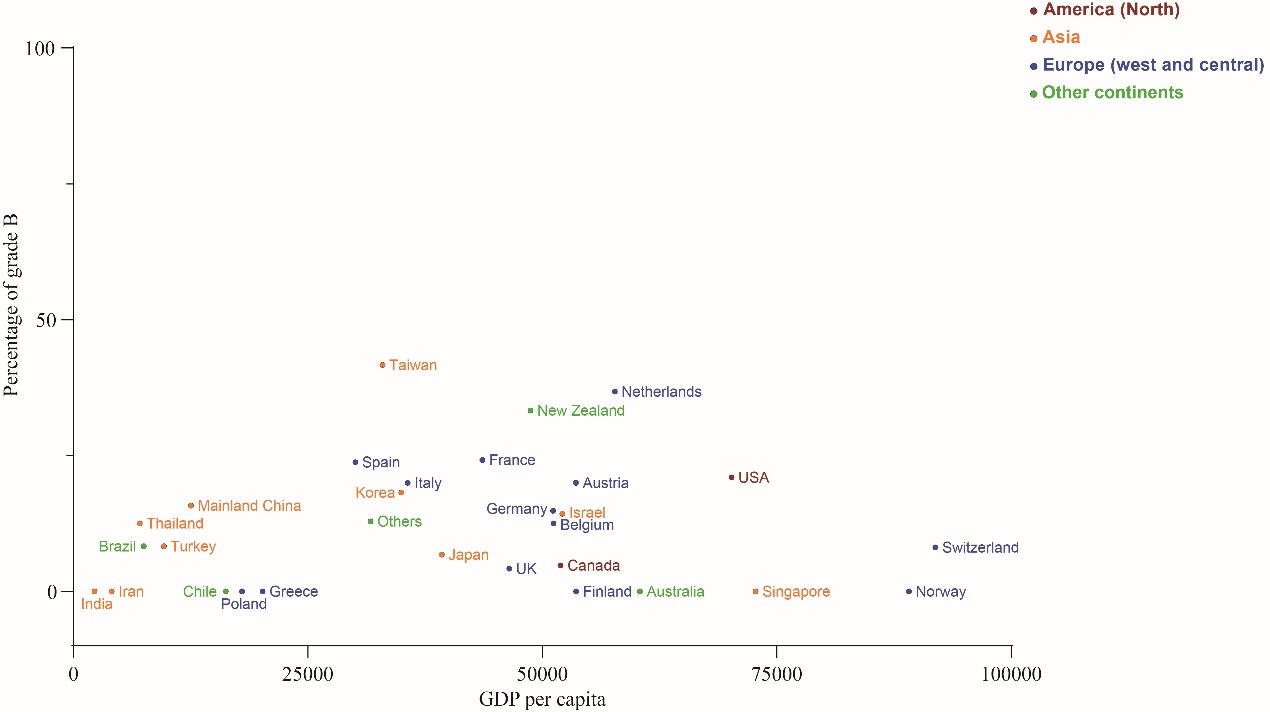
**

**Figure S6: Plot of percentage of grade C of individual country or regions (published more than 5 articles) by Gross Domestic Product (GDP) per capita in US$.**

**
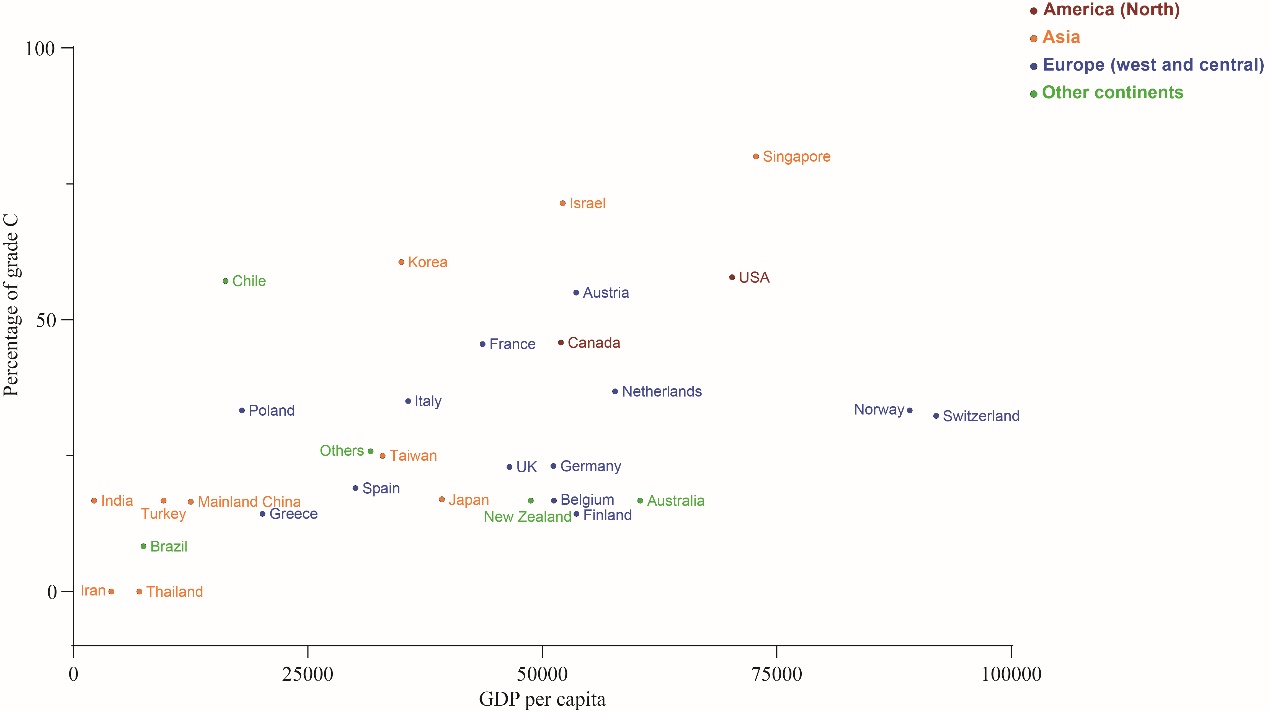
**

**Figure S7: Percentage of grade A across the US from 2017 to 2022.**


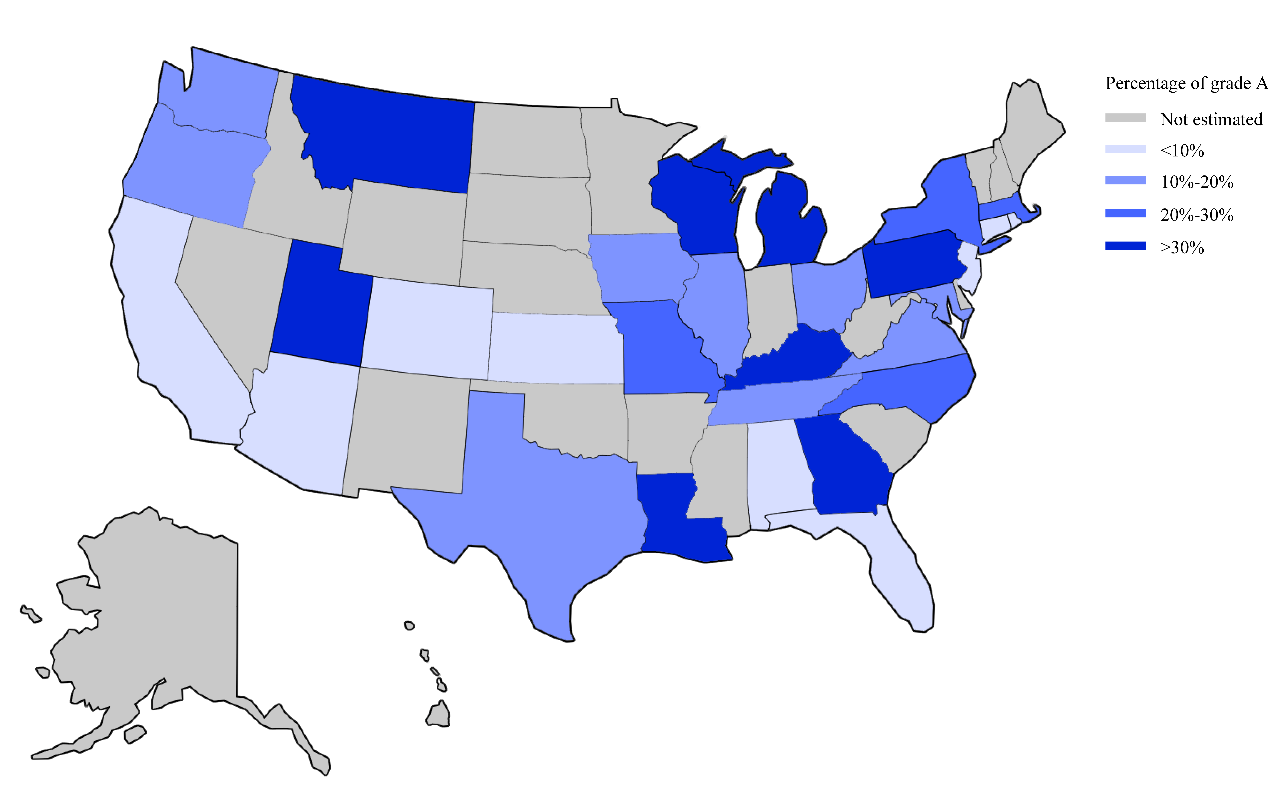

Supplement: Supplementary file 2 [file js9-111-1503-s002.docx]
